# Sleep disorders in rare genetic syndromes: a meta-analysis of prevalence and profile

**DOI:** 10.1186/s13229-021-00426-w

**Published:** 2021-02-25

**Authors:** Georgie Agar, Chloe Brown, Daniel Sutherland, Sean Coulborn, Chris Oliver, Caroline Richards

**Affiliations:** 1 School of Psychology, University of Birmingham, Birmingham, B15 2TT UK; 2 South West Yorkshire Partnership NHS Foundation Trust, Wakefield, WF1 3SP UK

**Keywords:** Meta-analysis, Sleep disorders, Intellectual disability, Genetic syndromes, Prevalence, Sleep profile

## Abstract

**Background:**

Sleep disorders are common in people with intellectual disability (ID) and autism, with growing evidence of diverse sleep profiles across ID associated genetic syndromes. Documenting the prevalence and profile of specific sleep disorders in syndromes will quantify syndrome-driven ‘risk’, inform prognosis and enhance understanding of aetiology of sleep disorders.

**Method:**

Following PRISMA guidelines for meta-analysis, we searched Ovid PsycINFO, Ovid MEDLINE, Ovid Embase, Web of Science and PubMed Central with use of syndrome-specific keywords and 60 sleep-related search terms. We screened and extracted papers that reported sleep disorder prevalence data for five or more individuals within a genetic syndrome, and applied quality criteria to produce a quality-effects prevalence model of six types of sleep disorder across nineteen syndromes. Relative risk estimates were calculated for the prevalence of each sleep disorder in each syndrome.

**Results:**

Two hundred and seventy three papers were identified, generating 463 prevalence estimates for Angelman, CHARGE, Cornelia de Lange, Down, fragile X, Prader–Willi, Rett, Smith–Magenis and Williams syndromes, mucopolysaccharidoses (MPS disorders), neurofibromatosis and tuberous sclerosis complex. Prevalence estimates were higher in genetic syndromes than published equivalents for typically developing individuals, with few exceptions. Between-syndrome differences for some disorders were evident; sleep-disordered breathing was most prevalent in MPS disorders (72–77%), while excessive daytime sleepiness was highest in Smith–Magenis syndrome (60%). Conversely, insomnia, which was reported at a higher rate than TD estimates in all syndromes except fragile X, was not associated with specific genetic risk. This suggests insomnia could emerge because of the individual’s environment or associated developmental delay, rather than any specific genetic syndromes.

**Limitations:**

Due to the broad scope of the meta-analysis, only syndromes previously identified as reporting preliminary sleep research were included. Other syndromes may also experience elevated prevalence rates of specific types of sleep disorder. Only English language papers were included.

**Conclusions:**

Differing prevalence rates between types of sleep disorder suggest differing causal mechanisms, such as cranio-facial morphology in Down and Prader–Willi syndromes and the build-up of mucopolysaccharides in MPS disorders. Priorities for clinical assessment and intervention for sleep disorders are discussed.

## Background

The prevalence of poor sleep in individuals with intellectual disability (ID) is between 8.5–31.4% for adults [1] and 16–42% for children [2], with similar estimates for individuals with autism [3, 4]. Variation in estimates is likely due to the age and living environment of participants [5] and the definition and assessment of poor sleep. These prevalence estimates are consistently higher than for typically developing (TD) individuals, due to a variety of risk factors associated with the presence of ID, and the common co-occurrence of autism [6]. The consequences of poor sleep in these groups are as significant as in TD populations and include deleterious effects on child learning and parental stress [7–9]. Recent literature has also begun to explore bi-directional relationships between sleep disorders/difficulties, painful health conditions [10–12] and challenging behavior [13], all of which are more common in people with ID.

Previous literature reviews suggest that genetic syndromes are related to elevated prevalence rates of diagnosable sleep disorders and ‘general’ sleep difficulties in ID. For example, Surtees et al. [14] meta-analysed fifteen studies comparing sleep time of individuals with ID to TD comparison groups and found that individuals with ID slept for 18 minutes less per night. Secondary analysis revealed this difference in sleep quantity was isolated to the studies comparing TD individuals to individuals with genetic syndromes, rather than individuals with heterogeneous ID (ID not associated with a given genetic syndrome, but resulting from a range of prenatal, perinatal and postnatal causes). Similarly, Tietze et al. [15] highlighted studies reporting on sleep quality in eight genetic syndromes associated with ID, with prevalence of poor sleep generally higher in these specific syndromes than in the heterogeneous (‘mixed’) ID group, and higher than other reported estimates of poor sleep in heterogeneous ID [16, 17]. However, despite the findings in these and other systematic reviews (see [5, 15, 18–20]) there have been no meta-analyses comparing the prevalence of sleep disorders between syndromes, or the profile of sleep disorders within syndromes.

It is hypothesised that for any syndrome genetic aetiology gives rise to biological changes which increase vulnerability to sleep disorders. Genetic syndromes are associated with anatomical, physiological and neurological differences. It is possible therefore that aspects of a syndrome, such as cranio-facial morphology, disruption to melatonin production or associated pain-related health conditions, may confer risk for a sleep disorder. For example, in Smith–Magenis syndrome, loss of function to the retinoic acid-induced 1 gene, caused by a deletion on the short arm of chromosome 17, or mutation of the retinoic acid-induced 1 gene, results in changes to individuals’ circadian rhythms [21, 22]. This results in an inverted melatonin release pattern and distinct sleep profile of early morning waking and excessive daytime sleepiness [12] for which many individuals are treated with exogenous melatonin [23]. Quantifying the prevalence of these specific sleep disorders in and between rare syndromes will inform causal models of the development of such sleep disorders. These may be specific to one syndrome (as in the inverted circadian rhythm of Smith–Magenis syndrome) or shared across syndromes with similar physical or behavioral phenotypes. Understanding these potential causes will maximize the limited available data on sleep in genetic syndromes to better inform causal models across syndromes and hence clinical practice.

Despite the heightened prevalence and deleterious consequences of poor sleep in individuals with ID, few studies document specific diagnosable sleep disorders in individuals with genetic syndromes. ‘General’ sleep difficulties are frequently described as part of a behavioral phenotype and stipulated as criteria for clinical diagnosis of Smith–Magenis, Prader–Willi and Angelman syndromes for example [24–26]. However, the descriptions of sleep difficulties often lack specificity despite the assessment of cause of poor sleep being crucial to identifying effective intervention strategies. As Wiggs [27] explains, common presenting symptoms of sleeplessness, hypersomnia or strange sleep behaviors may have different underlying causes and consequently require different treatments. If the wrong treatment for a non-specific sleep difficulty is unsuccessful, poor sleep may be presumed to be refractory and perhaps an inevitable aspect of the syndrome (diagnostic overshadowing). Given that parents of individuals with rare syndromes often cite sleep as an area for which they would like more information and support [28, 29] it is important to delineate the prevalence and profile of specific sleep disorders in these groups. Therefore, the primary aims of this systematic review and meta-analysis are to document: (1) between-syndrome and (2) within-syndrome differences in the profile and prevalence of diagnosable sleep disorders using systematic review and meta-analysis. Given that there are over 1000 genetic syndromes identified as associated with ID [30] it is not possible to review the available sleep data for all of these. Instead, only syndromes where there are likely to be sufficient data to produce reliable prevalence estimates of poor sleep will be selected.

There are a number of challenges to synthesizing the literature on sleep disorders across genetic syndromes. First, there are seven primary types of sleep disorder (and many subtypes) recognized in the International Classification of Sleep Disorders (ICSD-3, [31]) with potentially compromised identification of signs and symptoms in atypical populations. Additionally, caregivers and studies using broad screening tools often report concerns about sleep quality, time or behavior in individuals with ID [19, 32] which may not be aligned with formal diagnostic criteria for sleep disorders. Consequently, a third aim of the review is to synthesize data on the ‘general’ sleep difficulties that many studies have assessed, to complement the synthesis of the prevalence of formally recognized sleep disorders.

The methods of assessing sleep in TD and atypical populations vary significantly. Variability is sometimes driven by selection of a sleep assessment procedure specific to the suspected sleep diagnosis; the ‘gold standard’ approach of overnight polysomnography and pulse oximetry to assess sleep-related breathing difficulties [33] would not be as useful an assessment tool for excessive daytime sleepiness, though it may occur as part of a wider diagnostic procedure. However, some variation in methodology is also likely due to constraints on time and resources. Some methodological differences between studies of poor sleep in typical and atypical populations may be due to the differing levels of ability and associated problems in participant groups, such as willingness or ability to tolerate certain methods of data collection. These differing methodological variations will likely have an impact on the estimated prevalence rates of sleep disorders across syndromes. Therefore, the meta-analysis will use a quality-effects model to weight the meta-analysed data by an objective evaluation of methodological rigour.

In summary, despite the heightened prevalence and deleterious consequences of poor sleep in individuals with ID, and the potential to delineate syndrome-related causal models of sleep disorders, a meta-analysis of the prevalence of specific sleep disorders across genetic syndromes has not yet been conducted. Tietze et al. (2012)’s [15] clinical review of sleep disturbances in children with ‘mixed’ and ‘specific’ disabilities highlighted a wide range of estimates in eight genetic syndromes, but did not meta-analyse these estimates, making it difficult to draw empirical conclusions about prevalence. Furthermore, prevalence data were reported for grouped sleep disturbances rather than specific types of sleep disorders separately, and the quality of individual studies was not considered. This review will therefore extend the work of Tietze et al. [15], and others, to delineate the prevalence of sleep disorders and ‘general’ sleep difficulties within and across syndromes, while considering the quality of each study. These findings will help build causal models of poor sleep and identify priorities for assessment and intervention in each individual syndrome. The aims of the study are:

i) To compare the prevalence of ICSD-3 sleep disorders *between* genetic syndromes associated with ID, to further understanding of the potential cause of poor sleep in these groups.
ii) To describe the relative prevalence of ICSD-3 sleep disorders *within* each genetic syndrome, creating a sleep profile for each syndrome, and recommend syndrome-related methods of assessment and intervention.
iii) To synthesize available data on ‘general’ sleep difficulties in genetic syndromes.
iv) To determine the methodological rigour of the assessment of sleep in genetic syndromes, by examining the recruitment and sample characteristics of each study, the validity of the definition of the sleep disorder and the reliability of the methods used for assessment.

## Method

### Search strategy

Five databases were searched: PsychINFO (1967 to January 2020 Week 4), Embase (1974 to 31st January 2020), MEDLINE (1956 to 31st January 2020), Web of Science (1990 to 31st January 2020) and PubMed (all years), to encompass literature published across psychological, pharmacological and biomedical disciplines. A comprehensive review by Stores [34] indicated that preliminary sleep research, of varying methodological quality, has been conducted in a range of neurodevelopmental disorders, though these studies are by no means extensive or conclusive. To focus the present review on populations where sleep data were likely to be sufficient for robust meta-analysis of prevalence, only those neurodevelopmental disorders identified in [34] and associated with genetic causes (i.e., due to a deletion, mutation, mosaicism, addition or other change to a gene, chromosome or area of a chromosome) were included—21 genetic syndromes in total. This approach has been taken in other meta-analyses of clinical features in syndromes (e.g., [35]), but does necessarily limit sleep findings to these selected syndromes. Key search terms for each syndrome (see Table 1) were compiled from genetics home reference terms and [34] and cross-checked with [35]. Medical subject headings (MeSH terms) were used where available.

**Table 1:** Search terms for genetic syndromes

Syndrome	Search terms
Angelman syndrome (AS)	"Angelman*" OR "Angelman* syndrome" OR "Happy puppet syndrome" OR "Happy puppet"
CHARGE syndrome (CS)	"CHARGE" OR "CHARGE syndrome" OR "CHARGE association" OR "Hall-Hittner* syndrome" OR "Hall* Hittner* syndrome" OR "Coloboma"
*Cornelia de Lange syndrome (CdLS)	"Cornelia de Lange* syndrome" OR "CDLS" OR "De Lange* syndrome" OR "Branchmann-De Lange* syndrome" OR "BDLS" OR "Brachmann* syndrome" OR "Amstelodamensis typus degenerativus" OR "Amsterdam dwarf syndrome" OR "Amsterdam dwarfism" OR "Typus degenerativus amstelodamensis"
*Cri du Chat syndrome (CdC)	"Cri-du-Chat" OR "Cat cry syndrome" OR "5p minus syndrome" OR "Chromosome 5p deletion syndrome" OR "5p- syndrome; Monosomy 5p" OR "5p deletion syndrome" OR "Chromosome 5p- syndrome"
*Down Syndrome (DS)	"Down* syndrome" OR "Trisomy 21" OR "Trisomy G" OR "47,XX,+21" OR "47,XY,+2"
*Fragile X syndrome (FXS)	"Fragile X" OR "Fragile-X" OR "Fragile X syndrome" OR "FXS" OR "FRAXA syndrome" OR "AFRAX" OR "Martin-Bell* syndrome" OR "Marker X syndrome" OR "fraX syndrome" OR "fra(X) syndrome" OR "X-linked mental retardation" OR "Macroorchidism" OR "Escalante* syndrome" OR "Escalante*"
Hurler syndrome (Hurler)	"Hurler*" OR "Mucopolysaccharidosis Ih" OR "MPS1-H" OR "MPS1H" OR "Mucopolysaccharidosis type 1H" OR "Mucopolysaccharidosis type IH" OR "Hurler disease" OR "MPSIH"
Jacobsen syndrome (JS)	"Jacobsen syndrome" OR "Jacobsen*" OR "JBS" OR "Chromosome 11q deletion syndrome" OR "Partial 11q monosomy syndrome"
Juvenile neuronal ceroid-lipofuscinosis (JNCL)	"juvenile neuronal*" OR "JNCL" OR "Neuronal ceroid lipofuscinosis 3" OR "Juvenile neuronal ceroid lipofuscinosis" OR "Vogt Spielmeyer disease" OR "Spielmeyer Sjogren disease" OR "CLN3 disease"
Lesch-Nyhan syndrome (LNS)	"Lesch-Nyhan syndrome" OR "LNS" OR "HPRT deficiency" OR "HPRT1 deficiency" OR "HPRT deficiency, complete" OR "Hypoxanthine guanine phospho-ribosyltransferase 1 deficiency" OR "Lesch-Nyhan syndrome" OR "Lesch Nyhan disease"
Mucopolysaccharidosis Type II (MPS II)	"Hunter*" OR "Mucopolysaccharidosis type II" OR "MPS II" OR "Attenuated MPS" OR "Severe MPS II" OR "Hunter syndrome" OR "Iduronate 2-sulfatase deficiency" OR "I2S deficiency" OR "MPS 2"
Mucopolysaccharidosis Type IIIB (MPS IIIB)	"sanfilippo*" OR "Mucopolysaccharidosis type III" OR "Mucopoly-saccharidosis type 3" OR "Sanfilippo syndrome" OR "MPSIII" OR "Mucopolysaccharidosis type 3" OR "Sanfilippo disease"
Mucopolysaccharidosis Type IV (MPS IV)	"Morquio*" OR "Morquio syndrome B" OR "Mucopolysaccharidosis type IVB" OR "MPS IVB" OR "MPS 4B"
*Neurofibromatosis (NF)	"Neurofibromatosis" OR "Neurofibromatosis type 1" OR "Neurofibromatosis 1" OR "NF1" OR "Peripheral Neurofibromatosis" OR "Recklinghausen* disease" OR "Neurofibromatosis type 2" OR "Neurofibromatosis 2" OR "NF2" OR "Central neurofibromatosis" OR "Bilateral acoustic neurofibromatosis" OR "BANF" OR "Familial acoustic neuromas"
Norrie disease (Norrie)	"Atrophia bulborum hereditaria" OR "Pseudoglioma" OR "Episkopi blindness" OR "Norrie*" OR "Norrie-Warburg syndrome" OR "Anderson-Warburg syndrome" OR "NDP" OR "Fetal iritis syndrome"
*Prader–Willi syndrome (PWS)	"PWS" OR "Prader–Willi*" OR "Willi–Prader syndrome" OR "Prader–Labhart–Willi syndrome"
*Rett Syndrome (Rett)	"Rett*" OR "Rett* syndrome" OR "Rett* disorder" OR "RTS" OR "RTT" OR "Cerebroatrophic hyperammonemia" OR "Autism-dementia-ataxia-loss of purposeful hand use syndrome"
Smith–Lemli–Opitz syndrome (SLOS)	"Smith Lemli Opitz syndrome" OR "SLO syndrome" OR "7-Dehydrocholesterol reductase deficiency" OR "RSH syndrome" OR "SLOS" OR "Rutledge lethal multiple congenital anomaly syndrome" OR "Polydactyly, sex reversal, renal hypoplasia, and unilobular lung" OR "Lethal acrodysgenital syndrome"
Smith–Magenis syndrome (SMS)	"Smith–Magenis*" OR "smith magenis" OR "Chromosome 17p11.2 deletion syndrome" OR "17p- syndrome" OR "17p11.2 monosomy" OR "chromosome 17p deletion syndrome" OR "deletion 17p syndrome" OR "partial monosomy 17p" OR "SMS"
Tuberous Sclerosis Complex (TSC)	"Tuberous sclerosis" OR "Tuberous sclerosis syndrome" OR "Bourneville* disease" OR "Bourneville* phakomatosis" OR "Cerebral sclerosis" OR "Cerebral sclerosis syndrome" OR "Epiloia" OR "Sclerosis tuberose" OR "Tuberose sclerosis" OR "Tuberose sclerosis syndrome" OR "Tuberous sclerosis complex" OR "TSC" OR "TSS"
*Williams syndrome (WS)	"William*" OR "William* syndrome" OR "Beuren* syndrome" OR "Elfin Facies syndrome" OR "Hypercalcemia-Supravalvar Aortic Stenosis" OR "Infantile hypercalcemia" OR "Supravalvar aortic stenosis syndrome" OR "WBS" OR "Williams-Beuren* syndrome" OR "WMS" OR "WS" OR "WBS"

An * indicates a MeSH heading was also used for this syndrome within Ovid databases (PsychINFO, Embase, Medline)

Key search terms for sleep disorders (see Table 2) were adapted from Diagnostic and Statistical Manual of Mental Disorders (DSM–V), ICSD-3 and International Classification of Diseases (ICD-10) criteria. These were combined using the term ‘AND’ with search terms for each genetic syndrome, producing twenty-one separate searches in each of the five databases.

**Table 2:** Sleep search terms

Sleep	"sleep*" OR "Non-24-hour sleep–wake disorder" OR "Non-24-hour sleep–wake syndrome" OR "Non-24-hour sleep–wake rhythm disorder" OR "Free running disorder" OR "Hypernychthemeral disorder" OR "N24HSWD" OR "Non-24-hour circadian rhythm disorder" OR "somniloquy" OR "sleep talking" OR "night talking" OR "Subwakefulness Syndrome" OR "sub wakefulness syndrome" OR "hypnagogic hallucination*" OR "Confusional arousal*" OR "sleep enuresis" OR "nocturnal enuresis" OR "night enuresis" OR "night* wet*" OR "nocturnal bed wet*" OR "rapid* eye movement behavi* disorder*" OR "REM behavi* disorder*" OR "Nightmare disorder*" OR "dream anxiety disorder*" OR "nightmare syndr*" OR "Non* Rapid Eye Movement Arousal" OR "NREM arousal" OR "Nocturnal eat*" OR "nocturnal drink*" OR "night eat*" OR "night drink*" OR "nocturnal Bruxism" OR "sleep bruxism" OR "nocturnal tooth*" OR "nocturnal teeth*" OR "night* walking" OR "sleep terror*" OR "night* terror*" OR "Parasomni*" OR "circadian rhythm disorder*" OR "circadian rhythm sleep*" OR "CRSD" OR "Central Alveolar Hypoventilation" OR "central alveolar hypovent*" OR "Central hypoventilat*" OR "Narcolepsy" OR "narcolep*" OR "hypersomnolen*" OR "hypersomni*" OR "isomni*"

### Study selection

Initial searches produced 42,514 references. Each syndrome search was considered individually, so that only duplicate papers for that syndrome were removed. Duplicates across syndromes (i.e., where one paper reported on the prevalence of a sleep disorder in two syndromes) were not removed at this stage. After within-syndrome duplicates were removed, 37,372 articles remained. The titles and abstracts of these were screened using inclusion and exclusion criteria in Table 3, syndrome by syndrome.

**Table 3:** Inclusion and exclusion criteria to screen titles and abstracts

Inclusion criteria	Exclusion criteria
Empirical peer-reviewed studies	Conference proceedings, magazines, dissertations, review articles and books
Studies published or available in English	Studies only published or available in a language other than English
Title or abstract indicates that the study reports on sleep within the genetic syndrome	Title or abstract does not mention any sleep key terms OR does not mention the genetic syndrome
Sample with syndrome ≥ 5^a^	Sample with syndrome < 5
Studies reporting on live human participants	Studies reporting on animal participants, pure genetic or post-mortem studies

^a^Only those syndromes associated with ID were considered, though not all participants in each study had an ID

In total, 36,637 papers were manually excluded at abstract and title screening (see Additional file 1 for a full list of exclusion reasons). Following this initial screening, the remaining 735 articles underwent full-text screening to assess eligibility for inclusion in the meta-analysis.^1^ The criteria outlined in Table 3 were applied again, with some additional considerations (see Table 4). To ensure reliability, the searches, abstract and full-text screening and data extraction from papers retrieved between March 2017 and January 2020 were replicated by a second researcher, and the results compared. Where eligibility of a paper was unclear, inclusion/exclusion was discussed with the lead author and decided by consensus.

**Table 4:** Eligibility criteria to screen full-text for inclusion/exclusion in the meta-analysis

Inclusion criteria	Exclusion criteria
Study reports the number of participants with the genetic syndrome who met a clinical cut-off for that syndrome (not an associated syndrome e.g., Fragile X-associated tremor/ataxia syndrome)	Study does not report the number of participants who met a clinical cut-off for that syndrome
Study reports on a unique sample (or a potentially overlapping sample, but the proportion of overlap cannot be readily determined)	Study reports on exactly the same sample as reported in a previous study
Participants recruited without any specific bias (e.g., not recruited for a drug trial for a sleep disorder)	Participants were recruited due to an existing sleep disorder
Study reports the number of participants with the genetic syndrome who had a sleep disorder or difficulty	It is not possible to determine the number of participants with the sleep disorder or difficulty included in the study
Data in the study were extractable for meta-analysis (e.g., number of participants presenting sleep disorder)	Data in the study were not extractable for meta-analysis (e.g., mean questionnaire scores)
Sleep was reported as a naturally occurring phenomenon, not through manipulation	Sleep was reported as an adverse event of treatment or following experimental manipulation
Study reports the number of participants experiencing a sleep disorder, not solely the number taking medication to treat a sleep disorder	Study reports only the number of participants who were taking medication for sleep disorder
Study reports prevalence of sleep disorder or difficulty	Study reports only sleep times or architecture

**Fig. 1:**
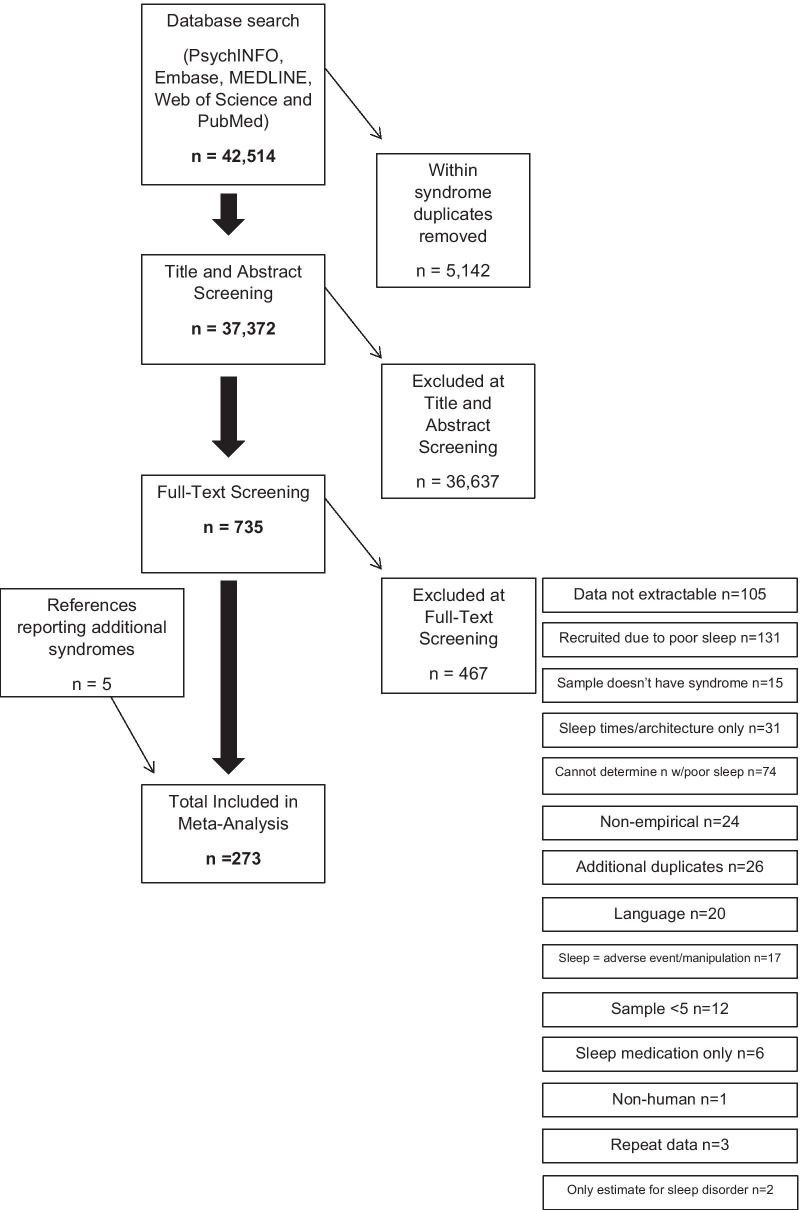
The search process in accordance with PRISMA guidance

A full list of exclusion reasons at full-text screening is provided in Fig. 1, which outlines the search process in accordance with the Preferred Reporting Items for Systematic Reviews and Meta-Analyses (PRISMA, [36]) guidelines. The number of papers included at each stage for each syndrome is summarised in Additional file 2.

To allow between-syndrome comparisons, specific sleep disorders described by the ICSD-3 were only included if there were data on the prevalence from at least two genetic syndromes. Therefore, the meta-analysis focuses on estimates for insomnia, sleep-related breathing disorders (obstructive and central sleep apnoeas), excessive daytime sleepiness^2^, sleep bruxism^3^ and sleep enuresis^4^. As only two papers considered circadian rhythm sleep disorders (one in Rett and one in Angelman syndrome) these disorders were not considered. Additionally, in accordance with the study aims, as 98 papers reported only on a ‘general’ sleep difficulty, this was also included as a subsection of the meta-analysis.

**Fig. 2:**
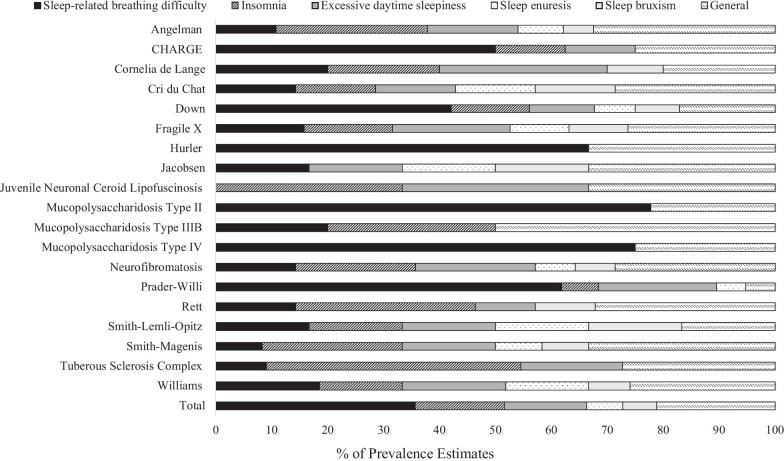
The percentage of papers producing estimates for each type of disorder or difficulty in each syndrome

As this was the first meta-analysis to consider sleep disorders in rare genetic syndromes, the decision was taken to include estimates for sleep disorders which were considered in at least two syndromes for within-syndrome analyses even if this represented the only estimate for that disorder in a given syndrome. This decision maximized inclusion of studies that consider sleep disorders in genetic syndromes. The number of papers that considered each sleep disorder and ‘general’ sleep difficulty in each syndrome is reported in Additional file 3, with the percentage of estimates for these presented in Fig. 2.

### Quality criteria

All 273 included papers were assessed for quality of sample characteristics to evaluate internal and external validity, based on criteria adapted from previous meta-analyses in rare syndromes [35, 37]. Additionally, for each sleep disorder assessed, specific quality criteria were applied, based on the diagnostic manuals of the ICSD-3 and relevant literature [33, 38, 39]. Specific quality criteria were also developed for papers that reported a ‘general’ sleep difficulty. For each paper the sample recruitment, confirmation of syndrome, definition of sleep disorder/difficulty and assessment of sleep disorder/difficulty were rated on a scale from 0 (poor) to 3 (excellent). Thus, a total score of 0–12 was possible. This total was divided by the maximum score of 12 to produce a quality rating, ranging from 0–1, with 0.00–0.25 rated as ‘poor’, 0.26–0.50 as ‘adequate’, 0.51–0.75 as ‘good’ and 0.76–1.00 as ‘excellent’. Each of the 273 papers included in the meta-analysis may have reported on multiple syndromes and multiple sleep disorders, resulting in a total of 463 prevalence estimates across syndromes and sleep disorders. Therefore, each paper may have had several quality criteria assigned to it related to each sleep disorder. The relevant quality rating for each sleep disorder was used when considering quality-effects during analysis.

To determine the inter-rater reliability of the quality criteria, papers retrieved between March 2017 and January 2020 (185 papers) were also rated independently for quality by a second rater. Mean weighted kappa = 0.711 (range 0.526–1.000). Quality criteria are described in full in Additional file 4.

### Analysis

To address the first three aims of the study, prevalence data were extracted from the final 273 papers to calculate pooled prevalence estimates of each type of sleep disorder and ‘general’ sleep difficulties. Using MetaXL 5.3 statistical software [40] both random-effects and quality-effects models of pooled prevalence were calculated, with 95% confidence intervals (CI). These were then compared within each syndrome, between syndromes and to TD estimates from the existing literature. This decision was taken to provide a clinical index of risk for specific sleep disorders in these syndromes. Given that very few studies used a control group, estimates were drawn from the most appropriate data available in the TD literature (i.e., presenting estimates for both children and adults, or using multiple methodologies to assess the same disorder, if these differed substantially). Pooled prevalence estimates could not be compared to estimates from the heterogeneous ID literature, firstly because data on specific sleep disorders are limited in this group (see [14]) and secondly because available data would likely include individuals with genetic syndromes considered in this meta-analysis, thus compromising the validity of comparisons.

Random-effects models assume that variability of prevalence estimates between included studies derives from differences in sampling error and study design, such as the number of participants included in each study [14]. Given the significant heterogeneity of prevalence estimates reported in the literature, this model was deemed more appropriate than the fixed-effects model, which assumes that differences in estimates are due to sampling error alone [41]. However, unlike quality-effects models, random-effects models do not take into account the credibility related heterogeneity of included studies. For brevity, prevalence estimates derived from the quality-effects models are reported in accordance with the fourth aim of the study.

To compare the profile of sleep disorders between syndromes, relative risk statistics were calculated. Due to the large number of comparisons made across sleep disorders and syndromes, 99.99% confidence intervals were calculated for relative risk analyses. Relative risks were considered significant if the confidence intervals did not include one [42].

## Results

### Study characteristics

The included 273 papers produced 463 prevalence estimates of five sleep disorders recognised by the ICSD-3 across nineteen genetic syndromes. In addition, 98 of these 273 papers reported the number of participants with ‘general’ sleep difficulties. These were considered to capture the less specific ‘clinical’ issues often reported by caregivers relating to reduced sleep quality and duration.

A total of 55,310 participants were included in the meta-analysis. Of the 273 papers, 113 reported on individuals up to and including 18 years old, while 23 papers focused on adults aged 19 and over. Samples of both children and adults were reported in 122 papers and the age range of the sample could not be determined for 15 papers. The full sample characteristics, quality ratings and outcome data for each type of sleep disorder/difficulty in each paper are presented in Additional file 5, with references provided in Additional file 6.

### Prevalence of sleep disorders across syndromes

To address the first three aims of the meta-analysis, the pooled prevalence of each sleep disorder and ‘general’ sleep difficulties in each syndrome was calculated (see Additional file 7). For brevity, quality-effects forest plots are presented alongside a more detailed overview of paper characteristics and syndrome-related sleep profiles in Additional files 8 and 9, respectively.

Pooled prevalence estimates for specific sleep disorders varied widely between and within syndromes, with estimates drawn from the quality-effects model largely more conservative than those drawn from the random-effects model. Higgins *I*^2^ revealed substantial heterogeneity in estimates for each of the sleep disorders. Heterogeneity refers to variability in prevalence rates that does not occur due to chance but due to genuine differences underlying study results [43]. As such, some heterogeneity is to be expected in meta-analyses, though the substantial heterogeneity here likely reflects many of the challenges of sleep assessment in individuals with rare syndromes.

Overall estimates for each sleep disorder ranged from 26% (sleep bruxism) to 45% (insomnia), according to the quality-effects model. In the majority of cases sleep disorders were more prevalent in genetic syndromes than in TD estimates. The prevalence of ‘general’ sleep difficulties across syndromes was 32%, similar to the estimate for insomnia in TD children (25%; [44]).

### Between-syndrome contrasts and relative risk analyses of rates of sleep disorders

**Fig. 3:**
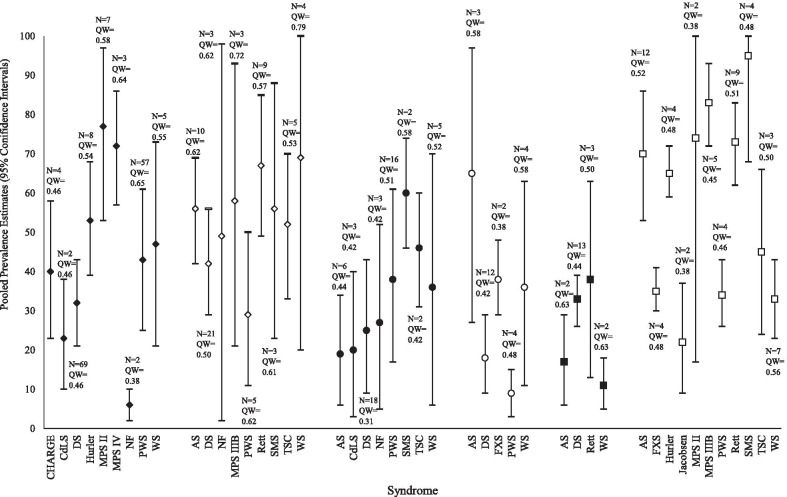
Quality-effects pooled prevalence estimates for all sleep disorders, with 95% confidence intervals for each genetic syndrome (abbreviated). *N* refers to the number of papers used to generate the pooled prevalence estimates, QW the mean quality rating of these papers. Filled diamonds represent the estimates for sleep-related breathing difficulties, unfilled the estimates for insomnia. Filled circles represent the estimates for excessive daytime sleepiness, unfilled the estimates for sleep enuresis. Filled squares represent the estimates for sleep bruxism, unfilled the estimates for ‘general’ sleep difficulties

To address the first and third aims of the meta-analysis, pooled prevalence estimates and relative risk for each type of sleep disorder and ‘general’ sleep difficulties were compared *between* genetic syndromes (see Fig. 3 and Table 5). Only syndromes with robust *pooled* prevalence estimates (i.e., estimates drawn from multiple studies with confidence intervals not including one, [35]) were included in this comparison.

**Table 5:** Relative risk statistics, with 99.99% confidence intervals for the prevalence of sleep disorders in each syndrome in comparison to all other syndromes

	Sleep related breathing difficulties	Excessive daytime sleepiness	Sleep enuresis	Sleep bruxism	‘General’ sleep difficulty
Angelman	/	− 0.32 (0.16–0.63) [SMS]	+ 7.22 (2.63–19.86) [PWS]	/	+ 3.18 (1.72–5.90) [JS] + 2.12 (1.30–3.45) [WS] + 2.06 (1.28–3.32) [PWS] + 2.00 (1.25–3.20) [FXS]
CHARGE	+ 6.67 (1.85–24.08) [NF]	/	/	/	/
Cornelia de Lange	− 0.30 (0.17–0.54) [MPSII] − 0.32 (0.18–0.58) [MPSIV] − 0.43 (0.23–0.82) [Hurler]	− 0.33 (0.17–0.65) [SMS]	/	/	/
Down	+ 5.33 (1.44–19.72) [NF] − 0.42 (0.26–0.67) [MPSII] − 0.44 (0.27–0.73) [MPSIV]	− 0.42 (0.23–0.75) [SMS]	/	+ 3.00 (1.12–8.04) [WS]	/
Fragile X	/	/	+ 4.22 (1.46–12.21) [PWS]	/	− 0.37 (0.24–0.57) [SMS] − 0.42 (0.27–0.66) [MPSIIIB] − 0.48 (0.30–0.76) [Rett] − 0.50 (0.31–0.80) [AS] − 0.54 (0.33–0.87) [Hurler]
Hurler	+ 8.83 (2.50–31.18) [NF] − 0.69 (0.49–0.96) [MPSII] − 0.74 (0.52–1.04) [MPSIV]	/	/	/	+ 2.95 (1.58–5.53) [JS] + 1.97 (1.20–3.24) [WS] + 1.91 (1.17–3.11) [PWS] + 1.86 (1.15–3.00) [FXS]
Jacobsen	/	/	/	/	− 0.23 (0.13–0.42) [SMS] − 0.27 (0.15–0.48) [MPSIIIB] − 0.30 (0.16–0.56) [Rett] − 0.31 (0.17–0.58) [AS] − 0.34 (0.18–0.63) [Hurler]
MPS II	+ 12.83 (3.72–44.29) [NF] + 3.35 (1.85–6.05) [CdLs] + 2.41 (1.49–3.90) [DS]	/	/	/	/
MPS IIIB	/	/	/	/	+ 3.77 (2.07–6.88) [JS] + 2.52 (1.58–4.00) [WS] + 2.44 (1.55–3.84) [PWS] + 2.37 (1.52–3.70) [FXS] + 1.84 (1.27–2.67) [TSC]
MPS IV	+ 12.00 (3.47–41.56) [NF] + 3.13 (1.72–5.70) [CdLs] + 2.25 (1.38–3.68) [DS]	/	/	/	/
Neurofibromatosis	− 0.08 (0.02–0.27) [MPSII] − 0.08 (0.02–0.29) [MPSIV] − 0.10 (0.03–0.37) [Hurler] − 0.13 (0.04–0.45) [WS] − 0.14 (0.04–0.50) [PWS] − 0.15 (0.04–0.54) [CHARGE] − 0.19 (0.05–0.69) [DS]	/	/	/	/
Prader–Willi	+ 7.17 (2.00–25.72) [NF]	/	− 0.14 (0.05–0.38) [AS] − 0.24 (0.08–0.69) [FXS]	/	− 0.36 (0.23–0.55) [SMS] − 0.41 (0.26–0.65) [MPSIIIB] − 0.47 (0.29–0.75) [Rett] − 0.49 (0.30–0.78) [AS] − 0.52 (0.32–0.85) [Hurler]
Rett	/	/	/	/	+ 3.32 (1.80–6.13) [JS] + 2.21 (1.37–3.58) [WS] + 2.15 (1.34–3.44) [PWS] + 2.09 (1.31–3.31) [FXS]
Smith–Magenis	/	+ 3.16 (1.59–6.29) [AS] + 3.00 (1.54–5.86) [CdLs] + 2.40 (1.33–4.35) [DS]	/	/	+ 4.32 (2.40–7.77) [JS] + 2.88 (1.84–4.50) [WS] + 2.79 (1.80–4.33) [PWS] + 2.71 (1.77–4.17) [FXS] + 2.11 (1.49–3.00) [TSC]
Tuberous Sclerosis Complex	/	/	/	/	− 0.47 (0.33–0.67) [SMS] − 0.54 (0.37–0.79) [MPSIIIB]
Williams	+ 7.83 (2.20–27.90) [NF]	/	/	− 0.33 (0.12–0.89) [DS]	− 0.35 (0.22–0.54) [SMS] − 0.40 (0.25–0.63) [MPSIIIB] − 0.45 (0.28–0.73) [Rett] − 0.51 (0.31–0.83) [Hurler] − 0.57 (0.29–0.77) [AS]

+ sleep disorder is significantly more prevalent than in one other syndrome; − sleep disorder is significantly less prevalent than in one other syndrome. / sleep disorder not assessed in that syndrome, or no difference in relative risk. There were no differences between syndromes in relative risk for insomnia

Relative risk analyses revealed no syndrome was at elevated risk of insomnia compared to any other. However, the relative risk of sleep-related breathing difficulties (SRBD) was notably significantly higher in MPS II (77%) than in three syndromes, including Down (32%) and Prader–Willi syndromes (43%). The relative risk of excessive daytime sleepiness was higher in Smith–Magenis syndrome (60%) than in neurofibromatosis (27%), Angelman (19%) and Cornelia de Lange (20%) syndromes. Additionally, the risk of having ‘general’ sleep difficulties was higher in Smith–Magenis syndrome (95%) than in five other syndromes. The risk of ‘general’ sleep difficulties was lowest in fragile X (35%), Jacobsen (22%), Prader–Willi (34%) and Williams (33%) syndromes, where risk was lower than five other syndromes.

### Comparison of prevalence rates of sleep disorders within genetic syndromes

**Fig. 4:**
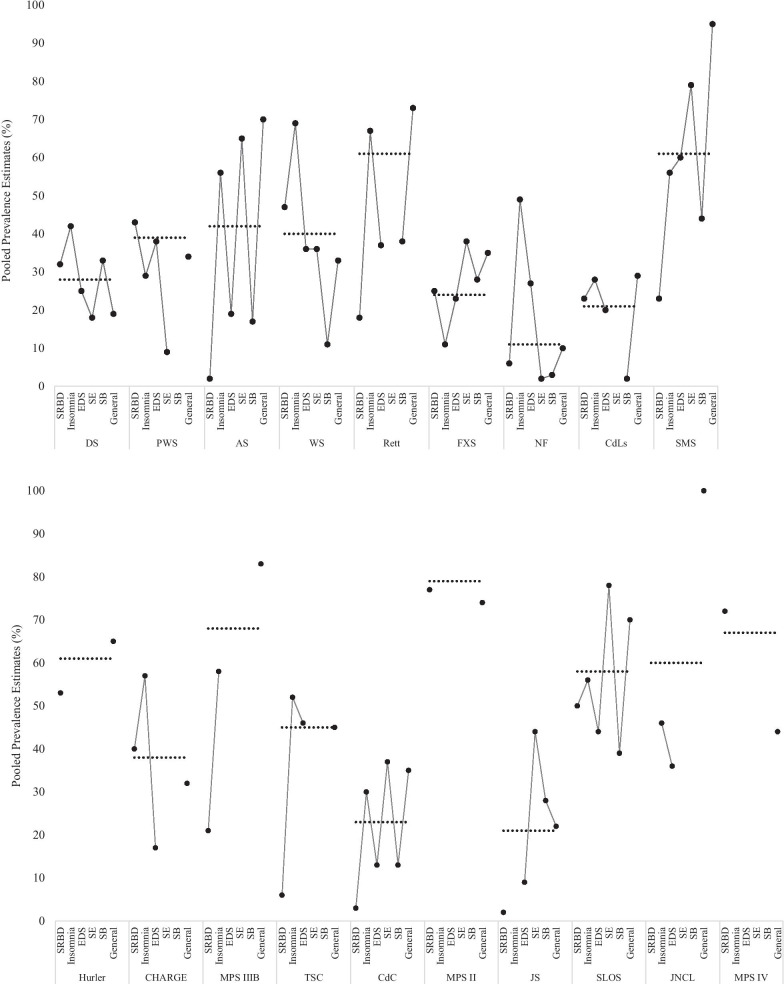
The profile of sleep disorders and ‘general’ sleep difficulties in each syndrome. The black circles represent the pooled prevalence estimates from the quality-effects model, with the overall prevalence of any sleep disorder/difficulty in each syndrome according to the quality-effects model presented as a dotted line for comparison. Abbreviations for sleep disorders and syndromes are used throughout

To address the second aim of the meta-analysis, pooled prevalence estimates of each sleep disorder were compared *within* each syndrome and syndrome-related profiles of sleep disorder delineated (see Fig. 4).

As demonstrated in Fig. 4, there was variation in the profile of specific sleep disorders in each syndrome, thus the overall prevalence of poor sleep for each syndrome may mask specific issues. For example, though the overall prevalence of sleep disorders was particularly low in neurofibromatosis (11%), the prevalence of specific disorders ranges from 2% (sleep enuresis) to 49% (insomnia). Similar ranges in estimates were seen in tuberous sclerosis complex (6–52%), Angelman (2–65%) and CHARGE syndromes (17–57%).

## Discussion

This review is the first to detail the *prevalence* of sleep disorders and ‘general’ sleep difficulties in rare genetic syndromes associated with ID. The findings extend those of Tietze et al. [15] and Surtees et al. [14] who report elevated risk of poor sleep in individuals with ID and neurodevelopmental disorders in comparison to TD individuals. Additionally, this is the first meta-analysis to consider the *profile* of sleep disorders within and between genetic syndromes. These profiles are clinically meaningful for parents and professionals working with individuals with specific syndromes. The conclusions drawn are strengthened by a robust search and screening process which allowed for inclusion of a large number of papers drawn from five databases. The addition of specific quality criteria with which to consider each of the sleep disorders/difficulties is a further strength of the meta-analysis.

To address the first aim of the meta-analysis, the prevalence of five specific sleep disorders was compared across nineteen genetic syndromes. Overall, with few exceptions, sleep disorders were more common in individuals with genetic syndromes than in TD adults [45] and children [44] with substantial variability in the prevalence of each type of sleep disorder in each syndrome. A brief summary of this variability and priorities for assessment and intervention is provided in Table 6.

**Table 6:** Clinical summary of elevated and equivocal risk for each type of sleep disorder in each syndrome

Syndrome	Sleep-related breathing difficulties	Insomnia	Excessive daytime sleepiness	Sleep Enuresis	Sleep bruxism
Angelman	+	++	+	++	+
CHARGE	++	–	–	–	–
Cornelia de Lange	++	++	+	–	–
Cri du Chat	–	–	–	–	–
Down	++	++	+	+	++
Fragile X	++	+	+	++	++
Hurler	++	–	–	–	–
Jacobsen	–	–	–	–	–
Juvenile Neuronal Ceroid Lipofuscinosis	–	–	–	–	–
Mucopolysaccharidosis Type II	++	–	–	–	–
Mucopolysaccharidosis Type IIIB	+	–	–	–	–
Mucopolysaccharidosis Type IVA	++	–	–	–	–
Neurofibromatosis	+	++	++	–	–
Prader–Willi	++	+	+	+	–
Rett	+	++	+	–	+
Smith–Magenis	–	+	++	–	–
Smith–Lemli–Opitz	–	–	–	–	–
Tuberous Sclerosis Complex	–	++	++	–	–
Williams	++	++	+	+	+

++ indicates a sleep disorder which should be considered as part of a priority assessment in that syndrome, + a sleep disorder which should be considered as part of routine assessment, and – a sleep disorder where further research is needed

These differences in prevalence rates for each type of sleep disorder suggest differing causal mechanisms. For example, the substantially elevated risk of SRBD in Mucopolysaccharidosis types II and IIIB is likely due to airway obstruction caused by build-up of mucopolysaccharides characteristic of the syndromes in the trachea and throat [46]. Taken together with the high prevalence of SRBD in Down and Prader–Willi syndromes associated with craniofacial morphology and hypotonia [47, 48] these data add to our understanding of the potential biological pathways from physical phenotype of a syndrome to sleep disorder. Similarly, the pooled prevalence rate of excessive daytime sleepiness in individuals with Smith–Magenis syndrome (60%) further supports the suggestion of an ‘inverted’ circadian rhythm and associated changes to the release pattern of melatonin, which is reported to peak in the middle of the day in this syndrome [21]. Given that melatonin is the hormone responsible for ‘sleepiness’ [49], typically released during darkness, peak release of this during the day would be expected to cause excessive daytime sleepiness.

In addition to strengthening these arguments, the data also allude to other potential biological mechanisms underlying sleep disorders. For example, excessive daytime sleepiness was highly prevalent in tuberous sclerosis complex (46%), which may be due to the high rates of epilepsy and use of anti-epileptic medication reported in this syndrome [50]. In Angelman syndrome, sleep enuresis, which occurred in 65% individuals, may be linked to the relatively low level of adaptive functioning and general continence seen in this group [51]. These prevalence rates therefore suggest that genetic aetiology of certain syndromes may carry elevated risk for sleep disorders with an underlying biological cause. These biological factors should be a primary target for sleep intervention, for example by working to improve daytime continence and seizure management in order to improve sleep. This is in contrast to much of the behavioural sleep literature, which suggests individuals with ID may be at greater risk of poor sleep due to environmental factors such as sleep hygiene and a reliance on parental involvement in settling and re-settling to sleep (e.g., [52, 53]).

Interestingly, in several syndromes where biological causes of sleep disorder are often well known and thus can be treated, such as SRBD in Down syndrome, or excessive daytime sleepiness in Smith–Magenis syndrome, other sleep disorders (sleep bruxism and sleep enuresis, respectively) were also identified as being highly prevalent in a smaller number of studies. This suggests that there may be other causes of poor sleep in these groups which do not receive as much research attention or clinical focus. These findings highlight the importance of rigorous assessment of presenting symptoms to identify underlying cause and thereby target the most efficacious treatment approach in these groups [27].

Conversely, insomnia, which was reported at a higher rate than TD estimates in all syndromes except fragile X, was not associated with specific genetic risk. This suggests insomnia could emerge because of the individual’s environment or associated developmental delay, rather than any particular genetic mechanism. This is reflected in the TD literature where insomnia, particularly of childhood, is often conceptualised as ‘behavioural’ and treated with behavioural intervention techniques [54]. Additional risk of insomnia may be conferred by high rates of epilepsy in individuals with ID [55] and the side effects of anti-epileptic and other medications [1]. The elevated prevalence of mental health difficulties [56] differences in sensory processing [57], mobility issues [52] and reduced influence of zeitgebers which entrain the sleep–wake cycle [58] may also contribute to risk. Furthermore, phenotypic behaviours associated with a given genetic syndrome may interact with the individual’s environment to elevate these difficulties. Interestingly, the rates of ‘general’ sleep difficulties did differ between syndromes, with particularly high pooled prevalence estimates in individuals with Angelman and Smith–Magenis syndromes. This suggests that there may be aspects of the behavioural phenotype of these syndromes which contribute to difficulties around sleep which do not map onto a specific sleep disorder. For example, the profile of sociability in both groups [59, 60] may lead to greater distress at bedtime or waking, even if the settling and waking issues do not in themselves meet criteria for insomnia. Elevated rates of ‘general’ sleep difficulties in these groups could also be caused by pain from physical health conditions which are prevalent in these syndromes and may disrupt sleep [61]. Broader assessment and intervention for these aspects of health and behavior may therefore be beneficial for individuals presenting with ‘general’ sleep difficulties.

To address the second aim of the meta-analysis, a sleep profile was described for nineteen genetic syndromes. Of all the syndromes Mucopolysaccharidosis types II and IIIB had the highest overall prevalence of any sleep disorder, largely driven by the high rates of SRBD. Distinguishing between these different types of sleep disorder is crucial for targeting priorities for assessment and tailoring intervention techniques as appropriate. Given that caregivers may not always be aware of individuals’ breathing at night-time, and the consequences of sleep disordered breathing include poor quality sleep, fatigue, inattention and excessive daytime sleepiness [62] it is important that these risks are identified rather than using behavioural techniques to treat an underlying biological problem. This is particularly important given that access to information about sleep is a priority for caregivers of children with rare genetic syndromes [28, 29]. In Mucopolysaccharidosis II in particular the relative risk of SRBD was 2.41 times greater than Down syndrome, which is considered to be at very high risk for SRBD, such that a proactive screening process has been called for in children with Down syndrome [63]. The results of this meta-analysis suggest these screening approaches are also warranted in other syndromes such as Mucopolysaccharidosis II, for which risk of experiencing these difficulties is even greater.

The final aim of this meta-analysis was to consider the quality of research investigating sleep disorders and difficulties in individuals with rare syndromes, through the development of a novel quality framework. Some papers received high quality ratings, utilising robust assessment techniques for well-defined sleep disorders, while others had recruited a sample without genetic confirmation of the syndrome or lacked clear definitions of the aspect of poor sleep assessed (for example, 98 papers reported a ‘general’ sleep difficulty). The majority of these papers were not reporting a composite score across a range of subscale scores investigating individual sleep issues, though Axelsson et al. [64], Breslin et al. [65] and Licis et al. [66] are notable high quality exceptions. Instead, these papers tended to describe a ‘general’ issue with sleep that was poorly-defined. Arguably, these ‘general’ difficulties could incorporate any number of sleep issues, including those defined by the ICSD and addressed separately by the meta-analysis. This is particularly concerning given that the prevalence estimates for specific disorders are elevated above TD estimates in every syndrome, with only a handful of exceptions. The assessments and interventions for these disorders are well-established, so it is unacceptable that a label of ‘general’ sleep difficulties in individuals with rare syndromes who are arguably at greater risk, may mask the underlying (and treatable) disorder, resulting in sleep hygiene interventions for possibly biological sleep disorders and vice-versa. This lack of specificity in sleep assessment and intervention would not be accepted for TD individuals and is arguably even more crucial for individuals with rare syndromes, given the associated health risks and impact on quality of life.

Additionally, in many cases only one study had looked at a particular sleep disorder in a specific syndrome, (for example, Maas et al. [67] in Cri du Chat syndrome). These studies could therefore not be meta-analysed and prevalence rates may not be robust. The lack of research also meant that two syndromes (Lesch-Nyhan and Norrie) were not included in the meta-analysis and could not contribute to the overall prevalence of sleep disorders in rare syndromes. Furthermore, the pooled prevalence estimates for specific sleep disorders drawn from the quality-effects model are predominantly more conservative than the estimates drawn from the random-effects model. This demonstrates the potential for poor study methodology to inflate prevalence estimates in rare syndromes. Therefore, there is a clear need for more cohort or case–control studies, using more robust definitions and assessments of specific sleep disorders in rare genetic syndromes.

In particular, case–control studies of individuals with Cri du Chat, Lesch-Nyhan and Norrie syndromes are warranted, to provide a more accurate profile of sleep disorders in these groups in comparison with typically developing peers. For syndromes where an elevated prevalence of ‘general’ sleep difficulties was identified, such as Angelman and Smith–Magenis syndromes, sleep studies combining objective and subjective assessments are required to further delineate factors contributing to poor sleep. In groups such as these where typical or ‘gold standard’ assessment approaches such as polysomnography may not be tolerated or appropriate, actigraphy should be considered as an objective measure of insomnia and, in combination with the Multiple Sleep Latency Test, excessive daytime sleepiness (see [68]). Sleep-related breathing difficulties can be assessed using finger pulse oximetry without the full range of polysomnography wires (see [69]). Further research is needed to determine the most appropriate methods of assessment for sleep bruxism, sleep enuresis and other sleep-related movement disorders and parasomnias in genetic syndromes associated with ID. Finally, across all syndromes these approaches should be applied in longitudinal designs to evaluate the relative stability of the presence and profile of sleep disorders in these high risk groups.

## Limitations

Consideration of the quality of the literature in this area has also identified some limitations to the scope of the meta-analysis. The syndromes included were identified by Stores [34] as having available sleep data, as reported in published studies available at the time. This strategy was taken to focus on syndromes where some data on sleep disorders were likely to be extractable for meta-analysis. This approach has been taken in other meta-analyses of clinical features in syndromes (e.g., [35]), but as Stores [34] acknowledges, other (lesser studied, lesser known or newly discovered) syndromes may also experience poor sleep which would not be pooled in this meta-analysis. Therefore, this meta-analysis provides a useful starting point from which later cross-syndrome comparisons could be made. As research in this field is extended, future reviews may consider combining our approach with a scoping search for any additional syndromes that were not identified in this review. Additionally, the search terms for each syndrome were generated using [34] and the Genetics Home Reference website, resulting in some syndromes where the genetic aetiology was included in the terms and others where this was not. It is therefore possible that some papers were missed in certain syndrome searches, though it is worth noting that these papers would likely have a genetic rather than clinical focus, and are therefore unlikely to have included sleep data extractable for meta-analysis. Furthermore, papers were only included if available in English, thus potentially biasing the pooled prevalence estimates toward English-speaking countries. However, < 1% of exclusions at title and abstract screening were due to the abstracts not being available in English and papers with abstracts but not-full texts available in English accounted for only 4% of exclusions at full-text screening. A final limitation is that pooled prevalence estimates for each sleep disorder were compared to TD estimates from the existing literature, rather than control groups of included studies. This was necessary given the lack of control group in the majority of included studies, but there are many reasons why these prevalence rates may not be directly comparable, including how TD cases were ascertained. Where possible the most comparable TD prevalence estimated was used.

## Conclusions

This meta-analysis is the first to compare the prevalence of ICSD-3 defined sleep disorders across nineteen rare genetic syndromes associated with ID. Additionally, this review documents the profile of sleep disorders across these syndromes, and highlights syndromes where the prevalence of poorly-defined ‘general’ sleep difficulties was comparatively high, including Angelman and Smith–Magenis syndromes. These robust descriptions will inform both research into the underlying aetiology of poor sleep in individuals with ID, and clinical practice for those working with individuals with these syndromes. The results of the meta-analysis highlight the need for a more detailed ‘syndrome-related’ sleep profile to be included in clinical and diagnostic criteria, and considered as part of assessment and intervention for poor sleep. Where the meta-analysis has highlighted a lack of robust sleep data for rarer syndromes, further research is warranted.

## Supplementary Information

**Additional file 1.** Number of exclusions at Title and Abstract Screening.

**Additional file 2.** Number of papers (n) included and excluded at each stage of the selection process.

**Additional file 3.** Number of papers reporting prevalence estimates of each type of sleep disorder or difficulty for each syndrome.

**Additional file 4.** Quality criteria for sample characteristics, ‘general’ sleep difficulties and each type of sleep disorder.

**Additional file 5.** Characteristics of each study.

**Additional file 6.** Included references.

**Additional file 7.** Summary of prevalence rates for each sleep disorder and ‘general’ sleep difficulties across nineteen genetic syndromes.

**Additional file 8.** Detailed sleep disorder forest plots.

**Additional file 9.** Detailed syndrome forest plots.

## Data Availability

All data generated or analysed during this study are included in this published article and its supplementary information files.

## Abbreviations

ID: Intellectual disability
TD: Typical development
OSA: Obstructive sleep apnoea
ICSD-3: International Classification of Sleep Disorders-Third Edition
PRISMA: Preferred Reporting Items for Systematic Reviews and Meta-Analyses
CI: Confidence intervals
SRBD: Sleep-related breathing difficulties

## Footnotes

1 Where papers reported on more than five participants with a syndrome that was not the focus of that particular search but was considered in the meta-analysis (e.g., a paper reporting on five or more participants with Hurler syndrome, as well as participants with Mucopolysaccharidosis Type II) this was included for both syndromes, whether or not it was returned in the second syndrome’s original search.

2 As only three papers reported specifically on either subtype of narcolepsy (two in Prader–Willi syndrome and one in tuberous sclerosis complex), only papers reporting the common complaint of excessive daytime sleepiness required for the diagnosis of central disorders of hypersomnolence were included.

3 Thirty-four papers reported on sleep-related movement disorders across twelve syndromes. However, only nine considered restless leg syndrome/periodic limb movement disorder, with more than two estimates in only one syndrome (four in Williams syndrome, one in Mucopolysaccharidosis Type II, one in Smith–Lemli–Opitz syndrome, one in fragile X syndrome, one in Angelman syndrome and one in Down syndrome). Therefore, these estimates could not be included, and sleep bruxism was the only sleep-related movement disorder considered in the meta-analysis.

4 Due to the range of behaviours included in different studies under the umbrella term ‘parasomnias’ (e.g., night terrors, night laughing, sleep walking), it was felt that meta-analysis of all estimates would not be appropriate. Therefore, only estimates for the disorder most commonly reported under this umbrella term, sleep enuresis, were included in the meta-analysis.

## References

[1] van de Wouw E, Evenhuis HM, Echteld MA (2012). Prevalence, associated factors and treatment of sleep problems in adults with intellectual disability: a systematic review. Res Dev Disabil.

[2] Wiggs L, Stores G (1996). Severe sleep disturbance and daytime challenging behavior in children with severe learning disabilities. J Intellect Disabil Res.

[3] Reynolds AM, Malow BA (2011). Sleep and autism spectrum disorders. PediatrClin N Am.

[4] Cohen S, Conduit R, Lockley SW, Rajaratnam SM, Cornish KM (2014). The relationship between sleep and behavior in autism spectrum disorder (ASD): a review. J NeurodevDisord.

[5] Didden R, Sigafoos J (2001). A review of the nature and treatment of sleep disorders in individuals with developmental disabilities. Res Dev Disabil.

[6] Mayes SD, Calhoun SL (2009). Variables related to sleep problems in children with autism. Res Autism SpectrDisord.

[7] Ashworth A, Hill CM, Karmiloff‐Smith A, Dimitriou D. A cross‐syndrome study of the differential effects of sleep on declarative memory consolidation in children with neurodevelopmental disorders. Dev Sci. 2017;20. https://www.ncbi.nlm.nih.gov/pmc/articles/PMC5347847/. Cited 18 Jul 2019. 10.1111/desc.12383 PMC5347847 26690566

[8] Chu J, Richdale AL (2009). Sleep quality and psychological wellbeing in mothers of children with developmental disabilities. Res Dev Disabil.

[9] Richdale A, Francis A, Gavidia-Payne S, Cotton S (2000). Stress, behaviour, and sleep problems in children with an intellectual disability. J Intellect Dev Disabil.

[10] Breau LM, Camfield CS (2011). Pain disrupts sleep in children and youth with intellectual and developmental disabilities. Res Dev Disabil.

[11] Ghanizadeh A, Faghih M (2011). The impact of general medical condition on sleep in children with mental retardation. Sleep Breath.

[12] Trickett J, Heald M, Oliver C, Richards C. A cross-syndrome cohort comparison of sleep disturbance in children with Smith–Magenis syndrome, Angelman syndrome, autism spectrum disorder and tuberous sclerosis complex. J Neurodev Disord. 2018;10. https://www.ncbi.nlm.nih.gov/pmc/articles/PMC5831859/. Cited 18 Jul 2019. 10.1186/s11689-018-9226-0 PMC5831859 29490614

[13] Cohen S, Fulcher BD, Rajaratnam SMW, Conduit R, Sullivan JP, St Hilaire MA (2018). Sleep patterns predictive of daytime challenging behavior in individuals with low-functioning autism. Autism Res.

[14] Surtees ADR, Oliver C, Jones CA, Evans DL, Richards C (2018). Sleep duration and sleep quality in people with and without intellectual disability: a meta-analysis. Sleep Med Rev.

[15] Tietze A-L, Blankenburg M, Hechler T, Michel E, Koh M, Schlüter B (2012). Sleep disturbances in children with multiple disabilities. Sleep Med Rev.

[16] Boyle A, Melville CA, Morrison J, Allan L, Smiley E, Espie CA (2010). A cohort study of the prevalence of sleep problems in adults with intellectual disabilities. J Sleep Res.

[17] Quine L (1991). Sleep problems in children with mental handicap. J Intellect Disabil Res.

[18] Esbensen AJ, Schwichtenberg AJ (2016). Sleep in neurodevelopmental disorders. Int Rev Res Dev Disabil.

[19] Spruyt K, Braam W, Curfs LM (2018). Sleep in Angelman syndrome: a review of evidence. Sleep Med Rev.

[20] Lee C-F, Lee C-H, Hsueh W-Y, Lin M-T, Kang K-T (2018). Prevalence of obstructive sleep apnea in children with Down syndrome: a meta-analysis. J Clin Sleep Med.

[21] De Leersnyder H, de Blois M-C, Claustrat B, Romana S, Albrecht U, von Kleist-Retzow J-C (2001). Inversion of the circadian rhythm of melatonin in the Smith–Magenis syndrome. J Pediatr.

[22] Potocki L (2000). Circadian rhythm abnormalities of melatonin in Smith–Magenis syndrome. J Med Genet.

[23] De Leersnyder H, Zisapel N, Laudon M (2011). Prolonged-release melatonin for children with neurodevelopmental disorders. PediatrNeurol.

[24] Holm VA, Cassidy SB, Butler MG, Hanchett JM, Greenswag LR, Whitman BY (1993). Prader–Willi syndrome: consensus diagnostic criteria. Pediatrics.

[25] Smith AC, Boyd KE, Brennan C, Charles J, Elsea SH, Finucane BM, Adam MP, Ardinger HH, Pagon RA, Wallace SE, Bean LJ, Stephens K (1993). Smith–Magenis Syndrome. GeneReviews® [Internet].

[26] Williams CA, Beaudet AL, Clayton-Smith J, Knoll JH, Kyllerman M, Laan LA (2006). Angelman syndrome 2005: updated consensus for diagnostic criteria. Am J Med Genet A.

[27] Wiggs L (2001). Sleep problems in children with developmental disorders. J R Soc Med.

[28] Pearson EV, Waite J, Oliver C (2018). Differences in the information needs of parents with a child with a genetic syndrome: a cross-syndrome comparison. J Policy Pract Intellect Disabil.

[29] Trickett J, Heald M, Oliver C (2017). Sleep in children with Angelman syndrome: parental concerns and priorities. Res Dev Disabil.

[30] Abbeduto L, McDuffie A, Armstrong CL, Morrow L (2010). Genetic syndromes associated with intellectual disabilities. Handbook of medical neuropsychology: applications of cognitive neuroscience.

[31] American Academy of Sleep Medicine (2005). International classification of sleep disorders. Diagnostic and coding manual.

[32] Hering E, Epstein R, Elroy S, Iancu DR, Zelnik N (1999). Sleep patterns in autistic children. J Autism Dev Disord.

[33] Sateia MJ (2014). International classification of sleep disorders-third edition: highlights and modifications. Chest.

[34] Stores G. Sleep disturbance in specific neurodevelopmental disorders. Sleep and its disorders in children and adolescents with a neurodevelopmental disorder: a review and clinical guide. Cambridge: Cambridge University Press; 2014. p. 79–158.

[35] Richards C, Jones C, Groves L, Moss J, Oliver C (2015). Prevalence of autism spectrum disorder phenomenology in genetic disorders: a systematic review and meta-analysis. Lancet Psychiatry.

[36] Moher D, Liberati A, Tetzlaff J, Altman DG (2009). Preferred reporting items for systematic reviews and meta-analyses: the PRISMA statement. BMJ Br Med J Publ Group.

[37] Royston R, Howlin P, Waite J, Oliver C (2017). Anxiety disorders in Williams syndrome contrasted with intellectual disability and the general population: a systematic review and meta-analysis. J Autism Dev Disord.

[38] Clarkson E (2016). The relationship between sleep and daytime behaviour in children with autism spectrum disorder.

[39] Martin BT, Williamson BD, Edwards N, Teng AY (2008). Parental symptom report and periodic limb movements of sleep in children. J Clin Sleep Med.

[40] Barendregt JJ, Doi SA. MetaXL user guide: Version 5.3.

[41] Lipsey MW, Wilson DB (2001). Practical meta-analysis.

[42] Andrade C (2015). A primer on confidence intervals in psychopharmacology. J Clin Psychiatry.

[43] Higgins JPT, Thompson SG, Deeks JJ, Altman DG (2003). Measuring inconsistency in meta-analyses. BMJ.

[44] Owens J (2007). Classification and epidemiology of childhood sleep disorders. Sleep Med Clin.

[45] Brown WD, Lee-Chiong TL (2005). Insomnia: prevalence and daytime consequences. Sleep: a comprehensive handbook.

[46] Kamin W (2008). Diagnosis and management of respiratory involvement in Hunter syndrome. ActaPaediatr.

[47] Trois MS, Marcus CL, Ch MBB (2009). Obstructive sleep apnea in adults with Down syndrome. J Clin Sleep Med.

[48] Sedky K, Bennett DS, Pumariega A (2014). Prader Willi syndrome and obstructive sleep apnea: co-occurrence in the pediatric population. J Clin Sleep Med.

[49] Utiger RD (1992). Melatonin—the hormone of darkness. N Engl J Med.

[50] van Eeghen AM, Numis AI, Staley BA, Therrien SE, Thibert RL, Thiele EA (2011). Characterizing sleep disorders of adults with tuberous sclerosis complex: a questionnaire-based study and review. Epilepsy Behav.

[51] Wheeler AC, Sacco P, Cabo R (2017). Unmet clinical needs and burden in Angelman syndrome: a review of the literature. Orphanet J Rare Dis.

[52] Jan JE, Owens JA, Weiss MD, Johnson KP, Wasdell MB, Freeman RD (2008). Sleep hygiene for children with neurodevelopmental disabilities. Pediatrics.

[53] Richdale A, Wiggs L (2005). Behavioral approaches to the treatment of sleep problems in children with developmental disorders: what is the state of the art?. Int J Behav Consult Ther.

[54] Sharma MP, Andrade C (2012). Behavioral interventions for insomnia: theory and practice. Indian J Psychiatry.

[55] Robertson J, Hatton C, Emerson E, Baines S (2015). Prevalence of epilepsy among people with intellectual disabilities: a systematic review. Seizure.

[56] Cooper S-A, McLean G, Guthrie B, McConnachie A, Mercer S, Sullivan F (2015). Multiple physical and mental health comorbidity in adults with intellectual disabilities: population-based cross-sectional analysis. BMC FamPract.

[57] Mazurek MO, Petroski GF (2015). Sleep problems in children with autism spectrum disorder: examining the contributions of sensory over-responsivity and anxiety. Sleep Med.

[58] Angriman M, Caravale B, Novelli L, Ferri R, Bruni O (2015). Sleep in children with neurodevelopmental disabilities. Neuropediatrics.

[59] Adams D, Horsler K, Oliver C (2011). Age related change in social behavior in children with Angelman syndrome. Am J Med Genet.

[60] Wilde L, Mitchell A, Oliver C (2016). Differences in social motivation in children with Smith–Magenis syndrome and Down syndrome. J Autism Dev Disord.

[61] Agar G, Oliver C, Trickett J, Licence L, Richards C (2020). Sleep disorders in children with Angelman and Smith–Magenis syndromes: the assessment of potential causes of disrupted settling and night time waking. Res Dev Disabil.

[62] Gottlieb DJ, Vezina RM, Chase C, Lesko SM, Heeren TC, Weese-Mayer DE (2003). Symptoms of sleep-disordered breathing in 5-year-old children are associated with sleepiness and problem behaviors. Pediatrics.

[63] Bull MJ (2011). Committee on Genetics. Health supervision for children with Down syndrome. Pediatrics.

[64] Axelsson EL, Hill CM, Sadeh A, Dimitriou D (2013). Sleep problems and language development in toddlers with Williams syndrome. Res Dev Disabil.

[65] Breslin JH, Edgin JO, Bootzin RR, Goodwin JL, Nadel L (2011). Parental report of sleep problems in Down syndrome. J Intellect Disabil Res.

[66] Licis AK, Vallorani A, Gao F, Chen C, Lenox J, Yamada KA (2013). Prevalence of sleep disturbances in children with neurofibromatosis type 1. J Child Neurol.

[67] Maas APHM, Didden R, Korzilius H, Braam W, Smits MG, Curfs LMG (2009). Sleep in individuals with Cri du Chat syndrome: a comparative study. J Intellect Disabil Res.

[68] Smith MT, McCrae CS, Cheung J, Martin JL, Harrod CG, Heald JL (2018). Use of actigraphy for the evaluation of sleep disorders and circadian rhythm sleep-wake disorders: an American Academy of Sleep Medicine Systematic Review, Meta-Analysis, and GRADE Assessment. J Clin Sleep Med.

[69] Ashworth A, Hill CM, Karmiloff-Smith A, Dimitriou D (2015). The importance of sleep: attentional problems in school-aged children with Down syndrome and Williams syndrome. Behav Sleep Med.

