# Supplementary material for: Sleep disorders in rare genetic syndromes: a meta-analysis of prevalence and profile
Source: Mol Autism. 2021 Feb 25;12:18. doi: 10.1186/s13229-021-00426-w (PMC7908701; doi:10.1186/s13229-021-00426-w)
Supplement: Supplementary file 1 — Additional file 1. Number of exclusions at Title and Abstract Screening. [file 13229_2021_426_MOESM1_ESM.docx]

|  | **Exclusion Reason** | | | | | | |
| --- | --- | --- | --- | --- | --- | --- | --- |
| **Syndrome** | **Not Empirical** | **Not English** | **No sleep and no syndrome** | **Sample = <5** | **Non-Human** | **Additional Duplicates** | **TOTAL** |
| Angelman syndrome (AS) | 251 | 251 | 171 | 81 | 67 | 72 | 656 |
| CHARGE syndrome (CS) | 1803 | 1803 | 8345 | 44 | 228 | 245 | 10863 |
| Cornelia de Lange syndrome (CdLS) | 34 | 34 | 70 | 42 | 15 | 9 | 171 |
| Cri du Chat syndrome (CdC) | 15 | 15 | 22 | 22 | 0 | 4 | 66 |
| Down Syndrome (DS) | 1221 | 1221 | 1267 | 184 | 197 | 242 | 3135 |
| Fragile X syndrome (FXS) | 433 | 433 | 1121 | 95 | 315 | 58 | 2025 |
| Hurler syndrome (Hurler) | 71 | 71 | 99 | 17 | 35 | 23 | 245 |
| Jacobsen syndrome (JS) | 26 | 26 | 133 | 10 | 4 | 12 | 186 |
| Juvenile neuronal ceroid-lipofuscinosis (JNCL) | 15 | 15 | 27 | 13 | 59 | 3 | 118 |
| Lesch-Nyhan syndrome (LNS) | 44 | 44 | 162 | 23 | 34 | 8 | 271 |
| Mucopolysaccharidosis Type II (MPS II) | 342 | 342 | 1241 | 42 | 45 | 63 | 1734 |
| Mucopolysaccharidosis Type IIIB (MPS IIIB) | 65 | 65 | 82 | 24 | 41 | 29 | 241 |
| Mucopolysaccharidosis Type IV (MPS IV) | 46 | 46 | 80 | 2 | 11 | 1 | 140 |
| Neurofibromatosis (NF) | 279 | 279 | 1174 | 323 | 136 | 51 | 1966 |
| Norrie disease (Norrie) | 21 | 21 | 63 | 20 | 11 | 4 | 120 |
| Prader-Willi syndrome (PWS) | 567 | 567 | 546 | 152 | 51 | 177 | 1503 |
| Rett Syndrome (Rett) | 606 | 606 | 745 | 190 | 187 | 152 | 1885 |
| Smith-Lemli-Opitz syndrome (SLOS) | 40 | 40 | 62 | 11 | 31 | 5 | 149 |
| Smith-Magenis syndrome (SMS) | 286 | 286 | 392 | 91 | 39 | 131 | 941 |
| Tuberous Sclerosis Complex (TSC) | 374 | 374 | 899 | 185 | 104 | 86 | 1667 |
| Williams syndrome (WS) | 2227 | 2227 | 6243 | 5 | 13 | 63 | 8555 |
| **TOTAL** | 8766 | 290 | 22944 | 1576 | 1623 | 1438 | 36637 |

Additional File 1
