# Supplementary material for: Sleep disorders in rare genetic syndromes: a meta-analysis of prevalence and profile
Source: Mol Autism. 2021 Feb 25;12:18. doi: 10.1186/s13229-021-00426-w (PMC7908701; doi:10.1186/s13229-021-00426-w)
Supplement: Supplementary file 2 — Additional file 2. Number of papers (n) included and excluded at each stage of the selection process. [file 13229_2021_426_MOESM2_ESM.docx]

Additional File 2

| *Number of papers (n) included and excluded at each stage of the selection process* | | | | | | | | |
| --- | --- | --- | --- | --- | --- | --- | --- | --- |
|  | **Identification** | | **Screening** | | **Eligibility** | | **Multiple Syndromes** | **Included** |
|  | *N identified in initial search* | *N after duplicates removed* | *N screened* | *N Excluded* | *N full texts read for eligibility* | *N Excluded* | *N included through hand search* | *N included in meta-analysis* |
| **AS** | 984 | 708 | 708 | 656 | 52 | 32 | 0 | 20 |
| **CHARGE** | 11434 | 10869 | 10869 | 10863 | 6 | 1 | 0 | 5 |
| **CdLS** | 212 | 177 | 177 | 171 | 6 | 2 | 0 | 4 |
| **CdC** | 83 | 70 | 70 | 66 | 4 | 2 | 0 | 2 |
| **DS** | 4493 | 3444 | 3444 | 3135 | 309 | 220 | 0 | 89 |
| **FXS** | 2334 | 2061 | 2061 | 2025 | 36 | 29 | 0 | 7 |
| **Hurler** | 333 | 260 | 260 | 245 | 15 | 5 | 1 | 11 |
| **JS** | 198 | 188 | 188 | 186 | 2 | 0 | 0 | 2 |
| **JNCL** | 133 | 122 | 122 | 118 | 4 | 3 | 1 | 2 |
| **LNS** | 296 | 272 | 272 | 271 | 1 | 1 | 0 | 0 |
| **MPS II** | 1972 | 1747 | 1747 | 1734 | 13 | 5 | 1 | 9 |
| **MPS IIIB** | 320 | 255 | 255 | 241 | 14 | 6 | 0 | 8 |
| **MPS IV** | 168 | 144 | 144 | 140 | 4 | 2 | 2 | 4 |
| **NF** | 2099 | 1976 | 1976 | 1966 | 10 | 4 | 0 | 6 |
| **Norrie** | 140 | 120 | 120 | 120 | 0 | 0 | 0 | 0 |
| **PWS** | 2180 | 1606 | 1606 | 1503 | 103 | 49 | 0 | 54 |
| **Rett** | 2522 | 1946 | 1946 | 1885 | 61 | 42 | 0 | 19 |
| **SLOS** | 165 | 152 | 152 | 149 | 3 | 1 | 0 | 2 |
| **SMS** | 1341 | 977 | 977 | 941 | 36 | 28 | 0 | 8 |
| **TSC** | 1915 | 1689 | 1689 | 1667 | 22 | 14 | 0 | 8 |
| **WS** | 9192 | 8589 | 8589 | 8555 | 34 | 21 | 0 | 13 |
| **Total** | 42514 | 37372 | 37372 | 36637 | 735 | 467 | 5 | 273 |
