# Supplementary material for: Sleep disorders in rare genetic syndromes: a meta-analysis of prevalence and profile
Source: Mol Autism. 2021 Feb 25;12:18. doi: 10.1186/s13229-021-00426-w (PMC7908701; doi:10.1186/s13229-021-00426-w)
Supplement: Supplementary file 3 — Additional file 3. Number of papers reporting prevalence estimates of each type of sleep disorder or difficulty for each syndrome. [file 13229_2021_426_MOESM3_ESM.docx]

Additional File 3

| *Number of papers reporting prevalence estimates of each type of sleep disorder or difficulty for each syndrome.* | | | | | | | | |
| --- | --- | --- | --- | --- | --- | --- | --- | --- |
|  | *Number of papers* | *Sleep-Related Breathing Difficulties* | *Insomnia* | *Excessive Daytime Sleepiness* | *Sleep Enuresis* | *Sleep Bruxism* | *‘General’ sleep difficulty* | *Number of estimates for any sleep disorder/difficulty* |
| **AS** | 20 | 4 | 10 | 6 | 3 | 2 | 12 | 37 |
| **CHARGE** | 5 | 4 | 1 | 1 | 0 | 0 | 2 | 8 |
| **CdLS** | 4 | 2 | 2 | 3 | 0 | 1 | 2 | 10 |
| **CdC** | 2 | 1 | 1 | 1 | 1 | 1 | 2 | 7 |
| **DS** | 89 | 69 | 23 | 19 | 12 | 13 | 28 | 164 |
| **FXS** | 7 | 3 | 3 | 4 | 2 | 2 | 5 | 19 |
| **Hurler** | 11 | 8 | 0 | 0 | 0 | 0 | 4 | 12 |
| **JS** | 2 | 1 | 0 | 1 | 1 | 1 | 2 | 6 |
| **JNCL** | 2 | 0 | 1 | 1 | 0 | 0 | 1 | 3 |
| **LNS** | 0 | 0 | 0 | 0 | 0 | 0 | 0 | 0 |
| **MPS II** | 9 | 7 | 0 | 0 | 0 | 0 | 2 | 9 |
| **MPS IIIB** | 8 | 2 | 3 | 0 | 0 | 0 | 5 | 10 |
| **MPS IV** | 4 | 3 | 0 | 0 | 0 | 0 | 1 | 4 |
| **NF** | 6 | 2 | 3 | 3 | 1 | 1 | 4 | 14 |
| **Norrie** | 0 | 0 | 0 | 0 | 0 | 0 | 0 | 0 |
| **PWS** | 54 | 47 | 5 | 16 | 4 | 0 | 4 | 76 |
| **Rett** | 19 | 4 | 9 | 3 | 0 | 3 | 9 | 28 |
| **SLOS** | 2 | 1 | 1 | 1 | 1 | 1 | 1 | 6 |
| **SMS** | 8 | 1 | 3 | 2 | 1 | 1 | 4 | 12 |
| **TSC** | 8 | 1 | 5 | 2 | 0 | 0 | 3 | 11 |
| **WS** | 13 | 5 | 4 | 5 | 4 | 2 | 7 | 27 |
| **Total** | 273 | 165 | 74 | 68 | 30 | 28 | 98 | 463 |
