# Supplementary material for: Sleep disorders in rare genetic syndromes: a meta-analysis of prevalence and profile
Source: Mol Autism. 2021 Feb 25;12:18. doi: 10.1186/s13229-021-00426-w (PMC7908701; doi:10.1186/s13229-021-00426-w)
Supplement: Supplementary file 4 — Additional file 4. Quality criteria for sample characteristics, ‘general’ sleep difficulties and each type of sleep disorder. [file 13229_2021_426_MOESM4_ESM.docx]

Additional File 4

| *Quality criteria for sample characteristics, ‘general’ sleep difficulties and each type of sleep disorder* | | | | | |
| --- | --- | --- | --- | --- | --- |
|  |  | **Quality Rating** | | | |
|  |  | **0** | **1** | **2** | **3** |
| **Sample Characteristics** | *Sample Recruitment* | Not specified/reported | Restricted or non-random sample, such as health clinics or a single school  OR  Single regional sample (e.g. regional parent support group) | Multiple restricted or non-random samples e.g., multi-region specialist clinics  OR  National non-random sampling e.g. national parent support groups | Random or total population sample  OR  Sleep data collected as part of wider dataset, reducing likelihood of response bias |
|  | *Confirmation of Syndrome* | Not specified/reported OR  Clinical diagnosis only suspected | Clinical diagnosis by ‘generalist’ e.g., General Practitioner or Paediatrician  OR  Parent confirmation of genetic diagnosis (e.g. through questionnaire) | Clinical diagnosis by ‘expert’ e.g., Clinical Geneticist or Specialist Paediatrician | Molecular/Cytogenetic/ Metabolic confirmation of diagnosis |
| **‘General’ Sleep Difficulty** | *Definition* | Not specified/reported | Sleep problems (not specific) rated as present/absent | Sleep problems with indication of frequency or duration of difficulty and impairment of functioning | Global sleep disorder cut-off scores |
|  | *Assessment* | Not specified/reported | Parent/professional (yes/no) | Non-validated questionnaire | Validated questionnaire with total scores (e.g. CSHQ, PSQ) |
| **Insomnia** | *Definition* | Not specified/reported | Sleeplessness items rated as present/absent | Sleeplessness items with indication of frequency or duration of difficulty | Based on diagnostic criteria, such as DSM-IV or V, or ICSD  OR  Sleeplessness items with indication of frequency and duration of difficulty or mention of daytime impairment |
|  | *Assessment* | Not specified/reported | Parent/professional opinion (yes/no) | Questionnaire items not described, or measure is not typically used to assess sleep  OR  Items do not include any of the three main symptoms and are not based on diagnostic criteria | Questionnaire items include any of the three main symptoms or are explicitly based on diagnostic criteria  OR  Standardised, validated or clinical measure  OR  Multiple methods e.g.  questionnaire, diagnostic interview, sleep diary, physiological measure |
| **Sleep Bruxism** | *Definition* | Not specified/reported | Complaint of tooth grinding during sleep | Complaint of tooth grinding during sleep with associated sound, tooth wear or discomfort | Complaint with medical difficulties ruled out |
|  | *Assessment* | Not specified/reported | Parent/professional opinion (yes/no) | Within general sleep questionnaire (e.g. PSQ, CSHQ)  OR non-validated specific questionnaire | Specific sleep bruxism validated questionnaire  OR  dental examination |
| **Sleep Enuresis** | *Definition* | Not specified/reported | Involuntary voiding of urine during sleep | Involuntary voiding of urine during sleep with indication of frequency [at least twice per month (age 3-6) and once per month (older than 6)] | Involuntary voiding of urine during sleep with indication of frequency and duration (to determine primary or secondary) |
|  | *Assessment* | Not specified/reported | Parent/professional opinion (yes/no) | Sleep and enuresis history  OR  within general sleep questionnaire (e.g. PSQ, CSHQ)  OR  Non-validated questions | Sleep and enuresis history with urine analysis  OR  specific validated questionnaire (e.g. The Parental Questionnaire: Enuresis/Urinary Incontinence) |
| **Excessive Daytime Sleepiness** | *Definition* | Not specified/reported | Excessive Daytime Sleepiness Items rated as present/absent | Mean sleep latency of < 8 min on the Multiple Sleep Latency Test | Mean sleep latency of < 8 min on the Multiple Sleep Latency Test + no obvious cause on 1 week of sleep diary/actigraphy |
|  | *Assessment* | Not specified/reported | Parent/professional (yes/no)  OR  Non validated questions  OR  Within wider sleep questionnaire | Multiple Sleep Latency Test OR  Epworth Sleepiness Scale | Multiple Sleep Latency Test + 1 week of sleep diary/actigraphy |
| **Sleep-Related Breathing Difficulties** | *Definition* | Not specified/reported | Sleep-related breathing difficulties items rated as present/absent | Sleep-related breathing difficulties items with indication of frequency or duration of difficulty  OR  alternative apnoea-hypopnea index (AHI) cut-offs  OR  AHI cut-off not given | Specified PSG AHI cut-offs:  Adults: AHI >15/h  OR  AHI >5/h with associated clinical symptoms  Children: AHI >1/h and one associated clinical symptom |
|  | *Assessment* | Not specified/reported | Parent/professional opinion (yes/no)  OR  non-validated questionnaire for issue/wider sleep problems (e.g. BEDS) | Polysomnography or pulse oximetry without defined cut-offs  OR  validated specific questionnaire (e.g. SRBDQ) | Polysomnography + pulse oximetry with defined cut-offs |
