# Supplementary material for: Sleep disorders in rare genetic syndromes: a meta-analysis of prevalence and profile
Source: Mol Autism. 2021 Feb 25;12:18. doi: 10.1186/s13229-021-00426-w (PMC7908701; doi:10.1186/s13229-021-00426-w)
Supplement: Supplementary file 5 — Additional file 5. Characteristics of each study. [file 13229_2021_426_MOESM5_ESM.docx]

Additional File 5

| *Characteristics of each study. QR = quality rating for that sleep disorder in that study. Age is presented in years unless otherwise specified.* | | | | | | | | | | | | | | | | | | | | | | | | | | | | | | |
| --- | --- | --- | --- | --- | --- | --- | --- | --- | --- | --- | --- | --- | --- | --- | --- | --- | --- | --- | --- | --- | --- | --- | --- | --- | --- | --- | --- | --- | --- | --- |
|  |  | **Sample Characteristics** | | | | | **Sleep-Related Breathing Difficulties** | | | | **Insomnia** | | | | **Excessive Daytime Sleepiness** | | | | **Sleep Enuresis** | | | | **Sleep Bruxism** | | | | **‘General’** | | | |
| Authors | Syndrome | N | M:F | MAge ±SD  (Range) | Recruitment | Confirmation | Definition | Assessment | % (n) | QR | Definition | Assessment | % (n) | QR | Definition | Assessment | % (n) | QR | Definition | Assessment | % (n) | QR | Definition | Assessment | % (n) | QR | Definition | Assessment | % (n) | QR |
| Abel & Tonnsen (2017) | AS | 18 | 9:9 | Median 18m (8-45m) | 3 | 1 | - | - | - | - | - | - | - | - | - |  | - | - |  | - | - | - | - | - | - | - | 1 | 1 | 50  (9) | 0.50 |
| Berry et al., (2005) | AS | 91 | 35:27 | 13.6  (1.3–40.7) | 1 | 3 | - | - | - | - | - | - | - | - | - | - | - | - | - | - | - | - | - | - | - | - | 1 | 1 | 68  (42)^[[1]](#footnote-1)^ | 0.50 |
| Bruni et al., (2004) | AS | 47 | 26:21 | 10.10  (2.3 – 26.2) | 1 | 3 | 1 | 1 | 19  (9) | 0.50 | 1 | 3 | 57  (27) | 0.67 | 1 | 1 | 23  (11) | 0.50 | - | - | - | - | - | - | - | - | - | - | - | - |
| Clarke & Marston (2000) | AS | 73 | 38:35 | 11  (5-33) | 2 | 1 | - | - | - | - | - | - | - | - | - | - | - | - | - | - | - | - | - | - | - | - | 1 | 1 | 42  (30)^[[2]](#footnote-2)^ | 0.42 |
| Conant et al., (2009) | AS | 290 | - | - | 3 | 0 | 2 | 1 | 3  (9) | 0.50 | 2 | 3 | 51  (149) | 0.67 | 1 | 1 | 12  (35) | 0.42 | - | - | - | - | - | - | - | - | - | - | - | - |
| Didden et al., (2004) | AS | 109 | 53:56 | 15.2  (2-44) | 2 | 3 | - | - | - | - | 2 | 3 | 40  (44) | 0.83 | 1 | 1 | 12  (13) | 0.58 | - | - | - | - | 2 | 2 | 10  (11) | 0.75 | 1 | 1 | 54  (59) | 0.58 |
| Goldman et al., (2012) | AS | 15 | 6:9 | 6.5  (2-16) | 1 | 3 | - | - | - | - | - | - | - | - | - | - | - | - | - | - | - | - | - | - | - | - | 1 | 1 | 100  (15) | 0.50 |
| Khan et al., (2019) | AS | 302 | 145:157 | 5.5 ± 5.9 | 3 | 3 | - | - | - | - | - | - | - | - | - |  | - | - |  | - | - | - | - | - | - | - | 0 | 1 | 80 (241) | 0.58 |
| Larson et al., (2015) | AS | 109 | 54:54^[[3]](#footnote-3)^ | 24.3  (16 – 50) | 3 | 1 | - | - | - | - | - | - | - | - | 1 | 1 | 56  (61) | 0.50 | - | - | - | - | - | - | - | - | 1 | 1 | 72  (78) | 0.50 |
| Miodrag & Peters (2015) | AS | 124 | 62:62 | -  (11m –27y)^[[4]](#footnote-4)^ | 2 | 0 | - | - | - | - | 2 | 1 | 61  (76) | 0.42 | - | - | - | - | - | - | - | - | - | - | - | - | - | - | - | - |
| Prasad et al., (2018) | AS | 53 | 25:28 | 25  (16-43) | 1 | 1 | - | - | - | - | - | - | - | - | - |  | - | - |  | - | - | - | - | - | - | - | 1 | 1 | 62 (33) | 0.33 |
| Radstaake et al., (2013) | AS | 71 | 36:35 | 20.5 ± 9.8  (6.8-45.8) | 2 | 1 | - | - | - | - | - | - | - | - | - | - | - | - | 2 | 3 | 87  (62) | 0.67 | - | - | - | - | - | - | - | - |
| Smith et al., (1996) | AS | 27 | 9:18 | 11.2  (3-34) | 2 | 3 | - | - | - | - | - | - | - | - | - | - | - | - | - | - | - | - | - | - | - | - | 1 | 1 | 86  (18)^[[5]](#footnote-5)^ | 0.58 |
| Sueri et al., (2017) | AS | 46 | 27:19 | 25  (14-45) | 2 | 3 | - | - | - | - | 1 | 3 | 52 (24) | 0.75 | - | - | - | - | - | - | - | - | - | - | - | - | 3 | 2 | 85  (39) | 0.83 |
| Summers et al., (1995) | AS | 11 | 6:5 | 5.4  - | 2 | 1 | - | - | - | - | 2 | 2 | 64  (7) | 0.58 | - | - | - | - | - | - | - | - | - | - | - | - | - | - | - | - |
| Tan et al., (2011) | AS | 92 | 50:42 | Median 33.5m  (5-60) | 3 | 3 | - | - | - | - | - | - | - | - | - | - | - | - | - | - | - | - | - | - | - | - | 1 | 1 | 80  (74) | 0.67 |
| Trickett et al., (2017) | AS | 50 | 20:30 | 7.02 ± 4 | 1 | 0 | 0 | 1 | 2  (1) | 0.33 | 1 | 1 | 68 (34) | 0.25 | 1 | 1 | 2  (1) | 0.25 | - | - | - | - | - | - | - | - | 1 | 1 | 90  (45) | 0.25 |
| Trickett et al., (2018) | AS | 70 | 32:38 | 8.64 ± 3.77 | 2 | 1 | - | - | - | - | 2 | 3 | 46 (32) | 0.67 | - | - | - | - | - | - | - | - | - | - | - | - | - | - | - | - |
| Wagner et al., (2017) | AS | 153 | 84:67^[[6]](#footnote-6)^ | 15.10 ± 7.16  - | 2 | 0 | - | - | - | - | - | - | - | - | - | - | - | - | 3 | 3 | 81^[[7]](#footnote-7)^  (115) | 0.67 | - | - | - | - | - | - | - | - |
| Walz et al., (2005) | AS | 339 | 165:174 | 10.98 (3-22) | 2 | 1 | 2 | 1 | 0  (0) | 0.50 | 2 | 3 | 48  (162) | 0.67 | 1 | 1 | 14  (48) | 0.42 | 1 | 1 | 40  (135) | 0.42 | 1 | 2 | 22  (73) | 0.50 | - | - | - | - |
| Deuce et al., (2012) | Charge | 44 | - | Mode 7  (1-15) | 3 | 2 | - | - | - | - | - | - | - | - | - | - | - | - | - | - | - | - | - | - | - | - | 1 | 1 | 14  (6) | 0.58 |
| Hartshorne et al., (2009) | Charge | 87 | 52:35 | 11y1m  (6-18) | 2 | 1 | 1 | 1 | 38  (34) | 0.42 | 1 | 3 | 57  (50) | 0.58 | 1 | 1 | 17  (15) | 0.42 | - | - | - | - | - | - | - | - |  |  |  |  |
| Hartshorne et al., (2016) | Charge | 53 | 33:20 | -  (13-39) | 3 | 0 | 1 | 1 | 25  (13) | 0.42 | - | - | - | - | - | - | - | - | - | - | - | - | - | - | - | - | 1 | 1 | 58  (31) | 0.42 |
| Roger et al., (1999) | Charge | 45 | 23:22 | - | 2 | 1 | 1 | 1 | 25  (11) ^[[8]](#footnote-8)^ | 0.42 | - | - | - | - | - | - | - | - | - | - | - | - | - | - | - | - | - | - | - | - |
| Trider et al., (2012) | Charge | 51 | 32:19 | 6y3m (20m – 14y) | 2 | 3 | 1 | 1 | 65  (33) | 0.58 | - | - | - | - | - | - | - | - | - | - | - | - | - | - | - | - | - | - | - | - |
| Hall et al., (2008) | CdLS | 54 | 46:12 | 13.88 ± 8.58 | 2 | 0 | - | - | - | - | - | - | - | - | - | - | - | - | - | - | - | - | - | - | - | - | 1 | 2 | 52  (28) | 0.42 |
| Rajan et al., (2012) | CdLS | 29 | - | 14.5  (0.6 – 37) | 2 | 0 | 2 | 3 | 48  (14) | 0.58 | - | - | - | - | 0 | 2 | 39  (12) | 0.33 | - | - | - | - | - | - | - | - | - | - | - | - |
| Stavinoha et al., (2011) | CdLS | 22 | 12:10 | 14.5  (1-37) | 2 | 0 | 1 | 2 | 32  (7) | 0.42 | - | - | - | - | 1 | 2 | 23  (5) | 0.42 | - | - | - | - | - | - | - | - | - | - | - | - |
| Zambrelli et al., (2016) | CdLS | 46 | 24:22 | 14.8  (4-46) | 1 | 3 | 1 | 1 | 17  (8) | 0.50 | 2 | 3 | 17  (8) | 0.75 | 1 | 1 | 9  (4) | 0.50 | - | - | - | - | 1 | 2 | 2  (1) | 0.58 | 1 | 3 | 15  (7) | 0.67 |
| Maas et al., (2009a) | CdC | 30 | 11:19 | 14y 3m  (2y 1m – 12y 6m) | 2 | 1 | 2 | 1 | 3  (1) | 0.50 | 2 | 3 | 30  (9) | 0.67 | 1 | 1 | 13  (4) | 0.42 | 2 | 2 | 37  (11) | 0.58 | 1 | 1 | 13  (4) | 0.42 | 1 | 1 | 63  (19) | 0.42 |
| Maas et al., (2012) | CdC | 25 | 8:17 | 13.5 ±  7.7  (3.8 – 28.5) | 2 | 0 | - | - | - | - | - | - | - | - | - | - | - | - | - | - | - | - | - | - | - | - | 1 | 3 | 12  (3) | 0.50 |
| Alexander et al., (2016) | DS | 6430 | 1573:1353^[[9]](#footnote-9)^ | 30.4% under 18^[[10]](#footnote-10)^ | 3 | 1 | 0 | 1 | 0.1  (21) | 0.42 | - | - | - | - | - | - | - | - | - | - | - | - | - | - | - | - | 0 | 1 | 2  (105) | 0.42 |
| Andreou et al., (2002) | DS | 12 | 6:6 | 21:66 ± 4:11  (17-30) | 1 | 0 | 2 | 3 | 100  (12) | 0.50 | - | - | - | - | 1 | 2 | 0  (0) | 0.33 | - | - | - | - | - | - | - | - | - | - | - | - |
| Areias et al., (2011) | DS | 45 | 22:23 | Median 13  (6-20) | 3 | 0 | - | - | - | - | - | - | - | - | - | - | - | - | - | - | - | - | 1 | 3 | 22  (10) | 0.58 | - | - | - | - |
| Ashworth et al., (2013) | DS | 22 | 11:11 | 9.42  (6.09 – 12.23) | 2 | 3 | - | - | - | - | 1 | 3 | 73  (16) | 0.75 | 1 | 1 | 86  (19) | 0.58 | 1 | 2 | 27  (6) | 0.67 | 1 | 2 | 45  (10) | 0.67 | 1 | 1 | 14  (3) | 0.58 |
| Austeng et al., (2014) | DS | 29 | 15:14 | 8  (8-8) | 3 | 3 | 2 | 3 | 66  (19) | 0.92 | - | - | - | - | - | - | - | - | - | - | - | - | - | - | - | - | - | - | - | - |
| Basil et al., (2016) | DS | 177 | 167:136^[[11]](#footnote-11)^ | 10.6  (2.14 – 19.1)^[[12]](#footnote-12)^ | 1 | 1 | 2 | 3 | 74  (131) | 0.58 | - | - | - | - | - | - | - | - | - | - | - | - | - | - | - | - | - | - | - | - |
| Bassell et al., (2015) | DS | 108 | 52:56 | 5.81  (1.50 – 13.40) | 1 | 0 | 1 | 1 | 18  (19) | 0.25 | 1 | 3 | 30  (16) | 0.42 | 1 | 1 | 37  (40) | 0.25 | 1 | 2 | 52  (56) | 0.33 | 1 | 2 | 36  (39) | 0.33 | 3 | 3 | 74  (39) | 0.58 |
| Bhatia et al., (2005) | DS | 40 | 26:14 | -^[[13]](#footnote-13)^ | 1 | 0 | - | - | - | - | - | - | - | - | - | - | - | - | - | - | - | - | - | - | - | - | 1 | 2 | 15  (6) | 0.33 |
| Breslin et al., (2011) | DS | 35 | 19:16 | 12.65  (7-18) | 2 | 1 | 1 | 1 | 60  (21) | 0.42 | 2 | 3 | 34  (12) | 0.67 | 1 | 1 | 26  (9) | 0.42 | - | - | - | - | 1 | 2 | 34  (12) | 0.50 | 3 | 3 | 86  (30) | 0.75 |
| Breslin et al., (2014) | DS | 38 | 15:23 | 9y7m  (7-12) | 2 | 1 | 2 | 3 | 50  (19)^[[14]](#footnote-14)^ | 0.66 | - | - | - | - | - | - | - | - | - | - | - | - | - | - | - | - | - | - | - | - |
| Brooks et al., (2015) | DS | 25 | 14:11 | 10.1  (2.2-18.7) | 1 | 0 | 2 | 3 | 76  (19) | 0.50 | - | - | - | - | 1 | 1 | 60  (15) | 0.25 | - | - | - | - | - | - | - | - | - | - | - | - |
| Capone et al., (2008) | DS | 23 | 20:3 | 7.8 ± 2.6  (3-13) | 1 | 3 | 1 | 1 | 4  (1) | 0.50 | 1 | 1 | 70  (16) | 0.50 | - | - | - | - | - | - | - | - | - | - | - | - | - | - | - | - |
| Capone et al., (2013) | DS | 37 | 19:18 | 21 ± 5  (14-30) | 1 | 3 | 3 | 3 | 76  (28) | 0.83 | - | - | - | - | - | - | - | - | - | - | - | - | - | - | - | - | - | - | - | - |
| Carskadon et al., (1993) | DS | 70 | 38:32 | 11.6 ± 4  (6-18) | 1 | 0 | 1 | 1 | 14  (10) | 0.25 | - | - | - | - | 1 | 1 | 11  (8) | 0.25 | 1 | 1 | 8  (6) | 0.42 | - | - | - | - | 1 | 1 | 4  (3) | 0.25 |
| Carter et al., (2009) | DS | 58 | 30:28 | 8.4  (0.7 – 17.9) | 3 | 0 | 0 | 1 | 25  (15) | 0.33 | 2 | 3 | 40  (16) | 0.67 | - | - | - | - | - | - | - | - | 1 | 2 | 45  (18) | 0.50 | 3 | 3 | 69  (40) | 0.75 |
| Chen et al., (2006) | DS | 20 | - | 26  (14-41) | 2 | 0 | 0 | 1 | 25  (4) | 0.25 | - | - | - | - | - | - | - | - | - | - | - | - | - | - | - | - | 1 | 1 | 20  (4) | 0.33 |
| Chen et al., (2013) | DS | 29 | 21:8 | 21.35  (14-31) | 3 | 0 | 2 | 1 | 41  (12) | 0.50 | - | - | - | - | - | - | - | - | - | - | - | - | - | - | - | - | - | - | - | - |
| Choi et al., (2019) | DS | 88 | 44:44 | 7.89 ± 3.03 | 2 | 0 | 2 | 1 | (12) | 0.42 | 2 | 3 | 25  (22) | 0.58 | 1 | 1 | 35  (31) | 0.33 | 1 | 2 | 23  (20) | 0.42 | 1 | 2 | 26  (23) | 0.42 | 3 | 3 | 83  (73) | 0.66 |
| Churchill et al., (2015) | DS | 110 | 83:27 | 11.05  (5-12) | 2 | 1 | 0 | 1 | 35  (38) | 0.33 | - | - | - | - | - | - | - | - | - | - | - | - | - | - | - | - | - | - | - | - |
| Cocchi (1989) | DS | 432 | 254:178 | -  (5-484) | 0 | 3 | - | - | - | - | - | - | - | - | - | - | - | - | - | - | - | - | 0 | 0 | 43  (186) | 0.25 | - | - | - | - |
| Cocchi (1996) | DS | 16 | 11:5 | 107.56m  (-) | 1 | 3 | - | - | - | - | 1 | 1 | 19  (3) | 0.50 | - | - | - | - | - | - | - | - | - | - | - | - | - | - | - | - |
| Cooper & Prasher (1998) | DS | 19 | 5:14 | 57  (42-72) | 3 | 0 | - | - | - | - | - | - | - | - | - | - | - | - | - | - | - | - | - | - | - | - | 1 | 2 | 68  (10) | 0.50 |
| Cornacchia et al., (2019) | DS | 56 | 31:25 | -^[[15]](#footnote-15)^ | 1 | 0 | 2 | 2 | 82  (46) | 0.42 | - | - | - | - | - | - | - | - | - | - | - | - | - | - | - | - | - | - | - | - |
| Cotton & Richdale (2006) | DS | 15 | 8:7 | 9.03  (3-16) | 1 | 0 | - | - | - | - | 1 | 3 | 33  (5) | 0.42 | 1 | 1 | 7  (1) | 0.25 | 1 | 2 | 0  (0) | 0.58 | - | - | - | - | 1 | 1 | 40  (6) | 0.25 |
| Dahlqvist et al., (2003) | DS | 17 | 10:7 | 5.9  (-) | 3 | 0 | 2 | 2 | 24  (4) | 0.58 | - | - | - | - | - | - | - | - | - | - | - | - | - | - | - | - | - | - | - | - |
| de Miguel-Díez et al., (2003) | DS | 108 | 69:39 | 7.9  (1-18) | 1 | 0 | 2 | 3 | 55  (59) | 0.50 | - | - | - | - | - | - | - | - | - | - | - | - | - | - | - | - | - | - | - | - |
| Dekker et al., (2018) | DS | 281 | 140:141 | 47  (-) | 2 | 1 | - | - | - | - | - | - | - | - | - | - | - | - | - | - | - | - | - | - | - | - | 2 | 2 | 22  (63) | 0.58 |
| Dudoignon et al., (2017) | DS | 57 | 31:26 | 6.2 ± 5.9 (-) | 1 | 0 | 2 | 3 | 72 (41) | 0.50 | - | - | - | - | - | - | - | - | - | - | - | - | - | - | - | - | - | - | - | - |
| Durhan et al., (2017) | DS | 18 | 8:10 | 10.4 ± 3.4  (-) | 1 | 0 | 2 | 3 | 61  (11) | 0.50 | - | - | - | - | - | - | - | - | - | - | - | - | - | - | - | - | - | - | - | - |
| Dyken et al., (2003) | DS | 19 | 9:10 | 8.8  (3-18) | 1 | 3 | 3 | 3 | 25  (15) | 0.83 | - | - | - | - | - | - | - | - | - | - | - | - | - | - | - | - | - | - | - | - |
| Edgin et al., (2015) | DS | 31 | 22:9 | 42 ± 10.3m  (27 – 64) | 0 | 3 | - | - | - | - | - | - | - | - | - | - | - | - | - | - | - | - | - | - | - | - | 1 | 1 | 68  (19)^[[16]](#footnote-16)^ | 0.42 |
| Elsharkawi et al., (2017) | DS | 47 | 29:18 | 9.1 ± 4.0 (-) | 0 | 0 | 3 | 3 | 49  (23) | 0.50 | - | - | - | - | - | - | - | - | - | - | - | - | - | - | - | - | - | - | - | - |
| Esbensen (2016) | DS | 75 | 49:26 | 51.1  (37-65) | 3 | 0 | 1 | 1 | 13  (10) | 0.42 | 2 | 3 | 23  (17) | 0.67 | - | - | - | - | - | - | - | - | - | - | - | - | - | - | - | - |
| Esbensen et al., (2016) | DS | 954 | 525:429 | 12.6  (5-21) | 1 | 1 | 0 | 0 | 27  (258) | 0.17 | - | - | - | - | - | - | - | - | - | - | - | - | - | - | - | - | - | - | - | - |
| Esbensen et al., (2018) | DS | 47 | 29:18 | 10.9 ± 3.1  (6-17) | 0 | 0 | 1 | 1 | 32  (15) | 0.17 | 1 | 3 | 34  (16) | 0.33 | 1 | 2 | 21  (10) | 0.25 | - | - | - | - | - | - | - | - | 3 | 3 | 85  (40) | 0.50 |
| Esbensen & Hoffman (2017) | DS | 30 | 18:12 | 11.68  (6-17) | 1 | 0 | 0 | 1 | 21  (6) | 0.17 | 1 | 3 | 20  (6) | 0.42 | 0 | 1 | 7  (2) | 0.17 | - | - | - | - | - | - | - | - | 3 | 3 | 17  (5) | 0.58 |
| Fan et al., (2017) | DS | 144 | - | 7.6 ± 8.5 | 1 | 1 | 2 | 3 | 78  (113) | 0.58 | - | - | - | - | - | - | - | - | - | - | - | - | - | - | - | - | - | - | - | - |
| Ferri et al., (1997) | DS | 10 | 7:3 | 16.9  (8.6 - 32.3) | 0 | 1 | 2 | 2 | 90  (9) | 0.42 | - | - | - | - | - | - | - | - | - | - | - | - | - | - | - | - | - | - | - | - |
| Friedman et al., (2018) | DS | 113 | 53:60 | 5.89  (-) | 1 | 1 | 2 | 1 | 66 (75) | 0.42 | - | - | - | - | - | - | - | - | - | - | - | - | - | - | - | - | - | - | - | - |
| Gimenez et al., (2018) | DS | 47 | 29:18 | 39.6 ± 12.4 | 2 | 0 | 2 | 3 | 72 (34) | 0.58 | - | - | - | - | 1 | 2 | 20  (8)^[[17]](#footnote-17)^ | 0.42 | - | - | - | - | - | - | - | - | 3 | 3 | 23  (9)^[[18]](#footnote-18)^ | 0.67 |
| Hayes et al., (2017) | DS | 193 | 97:96 | Median 31 (21.1-60.5) | 1 | 3 | 1 | 2 | 24  (46)^[[19]](#footnote-19)^ | 0.58 | - | - | - | - | - | - | - | - | - | - | - | - | - | - | - | - | - | - | - | - |
| Hill et al., (2016) | DS | 202 | 110:92 | 36m  (6-71 m) | 2 | 1 | 3 | 3 | 14  (28) | 0.75 | - | - | - | - | - | - | - | - | - | - | - | - | - | - | - | - | - | - | - | - |
| Hill et al., (2018) | DS | 161 | 86:75 | ^[[20]](#footnote-20)^ | 2 | 0 | 2 | 3 | 16 (25) | 0.58 | - | - | - | - | - | - | - | - | - | - | - | - | - | - | - | - | - | - | - | - |
| Hoffmire et al., (2014) | DS | 107 | 57:50 | -^[[21]](#footnote-21)^ | 1 | 0 | 0 | 1 | 30  (32) | 0.17 | 0 | 1 | 2  (2) | 0.17 | - | - | - | - | - | - | - | - | - | - | - | - | 3 | 3 | 65  (70) | 0.58 |
| Ikizoglu et al., (2017) | DS | 19 | 12:7 | 11.3 (10.3-14.3) | 2 | 1 | 3 | 3 | 32  (6) | 0.75 | - | - | - | - | - | - | - | - | - | - | - | - | - | - | - | - | - | - | - | - |
| Jayaratne et al., (2017) | DS | 63 | 35:28 | 7.49 ± 4.86 | 1 | 0 | 3 | 3 | 44 (23)^[[22]](#footnote-22)^ | 0.58 | - | - | - | - | - | - | - | - | - | - | - | - | - | - | - | - | - | - | - | - |
| Jensen et al., (2013) | DS | 62 | 39:23 | Median 33  (23-39) | 1 | 1 | 0 | 1 | 24  (15) | 0.25 | - | - | - | - | - | - | - | - | - | - | - | - | - | - | - | - | - | - | - | - |
| Jensen et al., (2015) | DS | 6869 | 3946:2923 | -^[[23]](#footnote-23)^ | 3 | 1 | 0 | 1 | 15  (1005) | 0.42 | - | - | - | - | - | - | - | - | - | - | - | - | - | - | - | - | - | - | - | - |
| Kavanagh et al., (1986) | DS | 74 | 23:51 | - | 1 | 0 | 1 | 1 | 29  (6) ^[[24]](#footnote-24)^ | 0.25 | - | - | - | - | - | - | - | - | - | - | - | - | - | - | - | - | - | - | - | - |
| Knollman et al., (2019) | DS | 766 | 432:334 | 4.1  (0.6 –6.2) | 1 | 1 | 3 | 3 | 48 (366) | 0.67 | - | - | - | - | - | - | - | - | - | - | - | - | - | - | - | - | - | - | - | - |
| Konstantinopolou et al., (2016) | DS | 27 | 14:13 | -  (8-21)^[[25]](#footnote-25)^ | 2 | 0 | 2 | 3 | 74  (20) | 0.58 | - | - | - | - | 1 | 2 | 44  (12) | 0.42 | - | - | - | - | - | - | - | - | - | - | - | - |
| Kuroda et al., (2017) | DS | 1149 | 650:499 | Median 14 (IQR:7.5 – 2.1) | 2 | 0 | 1 | 1 | 27 (312) | 0.33 | 1 | 2 | 58 (669) | 0.58 | 1 | 2 | 11 (131) | 0.42 | - | - | - | - | - | - | - | - | - | - | - | - |
| Lin et al., (2014) | DS | 208 | - | - | 3 | 0 | - | - | - | - | - | - | - | - | - | - | - | - | - | - | - | - | - | - | - | - | 0 | 2 | 15  (31) | 0.42 |
| Maas et al., (2009a) | DS | 30 | 22:8 | 17y4m ± 13y2m  (1y-55y8m) | 2 | 0 | - | - | - | - | 2 | 3 | 15  (3) | 0.58 | - | - | - | - | - | - | - | - | - | - | - | - | 1 | 1 | 24  (7) | 0.33 |
| Maas et al., (2012) | DS | 25 | 19:6 | 16.4  (4.2-41.5) | 2 | 0 | - | - | - | - | - | - | - | - | - | - | - | - | - | - | - | - | - | - | - | - | 3 | 3 | 4  (1) | 0.67 |
| Maris et al., (2016a) | DS | 52 | 70:52^[[26]](#footnote-26)^ | Median 5  (2.8 – 10.5)^[[27]](#footnote-27)^,^[[28]](#footnote-28)^ | 1 | 0 | 2 | 3 | 54  (28) | 0.50 | - | - | - | - | - | - | - | - | - | - | - | - | - | - | - | - | - | - | - | - |
| Maris et al., (2016b) | DS | 54 | 30:24 | Median 7.5  (5.4-11.6) | 1 | 0 | 2 | 3 | 57  (24) ^[[29]](#footnote-29)^ | 0.50 | 1 | 3 | 31  (17) | 0.42 | 1 | 1 | 39  (21) | 0.25 | 1 | 2 | 46  (25) | 0.67 | 0 | 1 | 31  (17) | 0.17 | 3 | 3 | 74  (40) | 0.58 |
| Mengoni & Redman (2019) | DS | 24 | 13:11 | 32m ± 15m  (10-65m) | 2 | 0 | 1 | 1 | 33  (8) | 0.33 | 0.42 | - | - | - | - | - | - | - | - | - | - | - | - | - | - | - | - | - | - | - |
| Miamoto et al., (2011) | DS | 60 | 37:23 | -^[[30]](#footnote-30)^ | 2 | 1 | - | - | - | - | - | - | - | - | - | - | - | - | - | - | - | - | 2 | 3 | 23  (14) | 0.67 | - | - | - | - |
| Nao et al., (2019) | DS | 36 | 19:17 | -^[[31]](#footnote-31)^ | 1 | 0 | 1 | 1 | 8  (3) | 0.25 |  |  |  |  |  |  |  |  |  |  |  |  |  |  |  |  |  |  |  |  |
| Ng et al., (2006) | DS | 22 | 15:7 | 20.82 ± 5.93  (-) | 2 | 3 | 2 | 3 | 59  (13) | 0.83 | - | - | - | - | - | - | - | - | 0 | 1 | 9  (2) | 0.33 | 0 | 1 | 23  (5) | 0.50 | - | - | - | - |
| Niemczyk et al., (2017) | DS | 317 | 189:128 | 19.2 ± 8.7  (4 – 51) | 3 | 0 | - | - | - | - | - | - | - | - | - | - | - | - | 3 | 3 | 17( 52)^[[32]](#footnote-32)^ | 0.25 | - | - | - | - | - | - | - | - |
| Nisbet et al., (2015) | DS | 130 | 75:55 | Median 5.8  (0.1 – 17.8) | 1 | 0 | 2 | 2 | 95  (124) | 0.42 | - | - | - | - | - | - | - | - | - | - | - | - | - | - | - | - | - | - | - | - |
| Ono et al., (2015) | DS | 90 | 47:43 | 16.6 ± 11.1  (-) | 1 | 0 | 1 | 1 | 25  (22) | 0.25 | - | - | - | - | - | - | - | - | - | - | - | - | - | - | - | - | - | - | - | - |
| Patti & Tsiouris (2006) | DS | 206 | 115:91 | -  (20-71) | 1 | 3 | - | - | - | - | - | - | - | - | - | - | - | - | - | - | - | - | - | - | - | - | 1 | 1 | 60  (124) | 0.50 |
| Posada et al., (2019) | DS | 53 | 27:26 | Median 3.4 (IQR: 1.6-8.8) | 1 | 0 | 2 | 3 | 91 (48) | 0.5 | - | - | - | - | - | - | - | - | - | - | - | - | - | - | - | - | - | - | - | - |
| Poskanzer et al., (2020) | DS | 73 | 31:42 | - | 1 | 0 | 3 | 3 | 12 (9) | 0.58 | - | - | - | - | - | - | - | - | - | - | - | - | - | - | - | - | - | - | - | - |
| Prasher & Filer (1995) | DS | 40 | 17:23 | 51.4 ± 9.4 | 0 | 3 | - | - | - | - | - | - | - | - | - | - | - | - | - | - | - | - | - | - | - | - | 2 | 2 | 0  (0) | 0.58 |
| Rahmawati et al., (2015) | DS | 32 | 14:18 | 18 ± 10  (4 – 40) | 1 | 0 | 2 | 2 | 25  (8) | 0.42 | 1 | 2 | 56  (18) | 0.33 | 1 | 2 | 56  (18) | 0.33 | - | - | - | - | - | - | - | - | - | - | - | - |
| Resta et al., (2003) | DS | 6 | - | 38.66  (28-53) | 0 | 3 | 2 | 3 | 83  (5) | 0.67 | - | - | - | - | - | - | - | - | - | - | - | - | - | - | - | - | - | - | - | - |
| Rosen et al., (2011) | DS | 250 | - | 5y8m  (-) | 1 | 0 | 1 | 1 | 52  (128) | 0.25 | 2 | 1 | 60  (153) | 0.33 | 1 | 1 | 54  (135) | 0.25 | - | - | - | - | - | - | - | - | - | - | - | - |
| Sawatari et al., (2015) | DS | 1,222 | 678:510^[[33]](#footnote-33)^ | 14.5 ± 9.9  (-) | 3 | 0 | 1 | 1 | 27  (325) | 0.42 | - | - | - | - | - | - | - | - | - | - | - | - | - | - | - | - | - | - | - | - |
| Shires et al., (2010) | DS | 52 | 28:24 | -^[[34]](#footnote-34)^ | 1 | 1 | 3 | 3 | 63  (33) | 0.67 | - | - | - | - | - | - | - | - | - | - | - | - | - | - | - | - | - | - | - | - |
| Shott et al., (2006) | DS | 56 | - | 42m  (20-63 m) | 3 | 0 | 3 | 3 | 38  (21) | 0.75 | - | - | - | - | - | - | - | - | - | - | - | - | - | - | - | - | - | - | - | - |
| Skotko et al., (2017) | DS | 102 | 61:41 | Median 5.6  (3-24.4) | 1 | 0 | 3 | 3 | 44  (45) | 0.58 | - | - | - | - | - | - | - | - | - | - | - | - | - | - | - | - | - | - | - | - |
| Sobey et al., (2015) | DS | 4081 | 2155:1926 | 18.5  (0-89) | 3 | 0 | 0 | 1 | 7  (270) | 0.33 | - | - | - | - | - | - | - | - | - | - | - | - | - | - | - | - | - | - | - | - |
| Stores (2019) | DS | 100 | 55:45 | 28 ± 11.4 (16-61) | 2 | 0 | 0 | 1 | 25 (24)^[[35]](#footnote-35)^ | 0.25 | 3 | 3 | 37 (37) | 0.67 | 1 | 2 | 38 (38) | 0.42 | 2 | 2 | 3  (3) | 0.5 | 1 | 2 | 39  (39) | 0.42 | - | - | - | - |
| Stores & Stores (2004) | DS | 46 | 22:24 | 2y8m  (7m – 4y 9m) | 2 | 0 | 2 | 1 | 15  (7) | 0.42 | 2 | 3 | 39  (18) | 0.58 | - | - | - | - | - | - | - | - | - | - | - | - | - | - | - | - |
| Stores & Stores (2014) | DS | 31 | 15:16 | 8.7  (2.3-16.3) | 1 | 0 | 2 | 1 | 13  (4) | 0.33 | 2 | 3 | 19  (6) | 0.50 | 0 | 1 | 6  (2) | 0.17 | 1 | 2 | 13  (4) | 0.33 | 1 | 2 | 23  (7) | 0.33 | - | - | - | - |
| Stores et al., (1996) | DS | 91 | 51:40 | 10.5  (4-19) | 1 | 0 | 2 | 1 | 12  (11) | 0.33 | 2 | 3 | 23  (21) | 0.50 | 0 | 1 | 8  (7) | 0.17 | 1 | 2 | 15  (14) | 0.33 | 1 | 2 | 16  (15) | 0.33 | - | - | - | - |
| Telakivi et al., (1984) | DS | 9 | 6:3 | Median 41  (25-65) | 0 | 0 | 1 | 1 | 78  (7) | 0.17 | - | - | - | - | - | - | - | - | - | - | - | - | - | - | - | - | - | - | - | - |
| Trois et al., (2009) | DS | 16 | 8:8 | Median 33  (19-56) | 2 | 1 | 2 | 2 | 94  (15) | 0.58 | - | - | - | - | - | - | - | - | - | - | - | - | - | - | - | - | - | - | - | - |
| Turner & Sloper (1996) | DS | 91 | 55:36 | 9y2m  (7-14) | 3 | 0 | - | - | - | - | 2 | 2 | 32  (29) | 0.58 | - | - | - | - | 2 | 2 | 19  (17) | 0.50 | - | - | - | - | 2 | 1 | 44  (40) | 0.50 |
| Urv et al., (2008) | DS | 161 | 35:126 | 51.6 ± 5.7  (-) | 3 | 3 | - | - | - | - | - | - | - | - | - | - | - | - | - | - | - | - | - | - | - | - | 1 | 1 | 17  (27) | 0.75 |
| Virji-Babul et al., (2007) | DS | 223 | 120:103 | -^[[36]](#footnote-36)^ | 2 | 0 | - | - | - | - | - | - | - | - | - | - | - | - | - | - | - | - | - | - | - | - | 2 | 2 | 4  (9) | 0.33 |
| Waters et al., (2020) | DS | 152 | 89:63 | 5.5 ± 4.5 years (1 week-17.8 years) | 2 | 1 | 2 | 3 | 86 (130) | 0.67 | - | - | - | - | - | - | - | - | - | - | - | - | - | - | - | - | - | - | - | - |
| Wong (2011) | DS | 107 | 432: 379^[[37]](#footnote-37)^ | 44  (18-79)^[[38]](#footnote-38)^ | 2 | 0 | 0 | 1 | 0.9  (1) | 0.25 | - | - | - | - | - | - | - | - | - | - | - | - | - | - | - | - | - | - | - | - |
| Yam et al., (2008) | DS | 407 | 232: 175 | 5.44  (0.06-17.16) | 3 | 0 | 0 | 1 | 7  (27) | 0.33 | - | - | - | - | - | - | - | - | - | - | - | - | - | - | - | - | 1 | 1 | 5  (200) | 0.42 |
| Yau et al., (2019) | DS | 104 | 52:52 | -^[[39]](#footnote-39)^ | 2 | 0 | - | - | - | - | 2 | 3 | 59 (61) | 0.58 | - | - | - | - | - | - | - | - | - | - | - | - | 2 | 3 | 45 (46)^[[40]](#footnote-40)^ | 0.58 |
| Kaufmann et al., (2017) | FXS | 328 | 256: 72 | -^[[41]](#footnote-41)^ | 3 | 0 | - | - | - | - | - | - | - | - | - | - | - | - | - | - | - | - | - | - | - | - | 1 | 1 | 35 (114) | 0.50 |
| Kronk et al., (2009) | FXS | 90 | 73:17 | -^[[42]](#footnote-42)^ | 2 | 0 | 1 | 1 | 33  (30) | 0.33 | 2 | 3 | 33  (30) | 0.58 | 1 | 1 | 36  (32) | 0.33 | 1 | 2 | 38  (34) | 0.42 | 1 | 2 | 33  (30) | 0.42 | 3 | 3 | 47  (42) | 0.67 |
| Kronk et al., (2010) | FXS | 1295 | 1013:282 | Males:15± 10.5  Females:16 ± 10.4  (-) |  |  | - | - | - | - | 3 | 3 | 10  (125) | 0.83 | 1 | 1 | 12  (49)^[[43]](#footnote-43)^ | 0.50 | - | - | - | - | - | - | - | - | 2 | 1 | 32  (413) | 0.58 |
| Richdale (2003) | FXS | 13 | 10:3 | 9.3  (3-19) | 1 | 0 | 2 | 1 | 0  (0) | 0.33 | 2 | 3 | 54  (7) | 0.50 | 1 | 2 | 8  (1) | 0.33 | 1 | 2 | 38  (5) | 0.33 | 1 | 2 | 0  (0) | 0.33 | 1 | 1 | 31  (4) | 0.25 |
| Symons et al., (2010) | FXS | 487 | 436: 51 | 15.9y ±  10.9  (-) | 3 | 1 | - | - | - | - | - | - | - | - | - | - | - | - | - | - | - | - | - | - | - | - | 1 | 1 | 39  (192) | 0.50 |
| Tawfik et al., (2009) | FXS | 16 | 16:0 | 10.81  (6-18) | 1 | 3 | - | - | - | - | - | - | - | - | 1 | 2 | 100  (16) | 0.58 | - | - | - | - | - | - | - | - | - | - | - | - |
| Tirosh & Borochowitz (1992) | FXS | 7 | 7:0 | 10  (6-21) | 1 | 3 | 2 | 1 | 29  (2) | 0.58 | - | - | - | - | - | - | - | - | - | - | - | - | - | - | - | - | - | - | - | - |
| Ahmed et al., (2014) | Hurler | 12 | 8:4 | -^[[44]](#footnote-44)^ | 3 | 3 | - | - | - | - | - | - | - | - | - | - | - | - | - | - | - | - | - | - | - | - | 1 | 0 | 50  (6) | 0.58 |
| Bax & Colville (1995) | Hurler | 49 | 39:24^[[45]](#footnote-45)^ | 6.5 ± 7  (0-41)^[[46]](#footnote-46)^ | 2 | 0 | - | - | - | - | - | - | - | - | - | - | - | - | - | - | - | - | - | - | - | - | 1 | 1 | 59  (29) | 0.33 |
| Dualibi et al., (2016) | Hurler | 9 | 5:4 | Median 8  (3 – 20) | 0 | 0 | 3 | 3 | 83  (5)^[[47]](#footnote-47)^ | 0.50 | - | - | - | - | - | - | - | - | - | - | - | - | - | - | - | - | - | - | - | - |
| Koehne et al., (2019) | Hurler | 9 | 3:6 | 7.56 (4-12) | 1 | 0 | 3 | 1 | 55  (5) | 0.42 | - | - | - | - | - | - | - | - | - | - | - | - | - | - | - | - | - | - | - | - |
| Lehtonen et al.,(2017) | Hurler | 21 | 10:21 | 9y1m ± 4y6m | 2 | 0 | 1 | 0 | 24 (5) | 0.25 | - | - | - | - | - | - | - | - | - | - | - | - | - | - | - | - | 1 | 3 | 57 (12) | 0.50 |
| Moreau et al., (2015) | Hurler | 10 | 6:4 | 6.55  (-) | 0 | 3 | 2 | 3 | 40  (4) | 0.67 | - | - | - | - | - | - | - | - | - | - | - | - | - | - | - | - | - | - | - | - |
| Muñoz -Rojas et al., (2011) | Hurler | 845 | - | - | 3 | 1 | - | - | - | - | - | - | - | - | - | - | - | - | - | - | - | - | - | - | - | - | 1 | 1 | 67  (570) | 0.50 |
| Pal et al., (2015) | Hurler | 61 | 38:23 | Median 82m  (0.3 – 420m) | 2 | 3 | 2 | 2 | 68  (36)^[[48]](#footnote-48)^ | 0.75 | - | - | - | - | - | - | - | - | - | - | - | - | - | - | - | - | - | - | - | - |
| Soni-Jaiswal et al., (2016) | Hurler | 11 | 4:7 | 7  (6m– 16y) | 1 | 0 | 1 | 1 | 73  (8) | 0.25 | - | - | - | - | - | - | - | - | - | - | - | - | - | - | - | - | - | - | - | - |
| Wraith et al., (2004) | Hurler | 45 | 22:23 | 15.5  (-) | 2 | 1 | 2 | 3 | 42  (19) | 0.67 | - | - | - | - | - | - | - | - | - | - | - | - | - | - | - | - | - | - | - | - |
| Wraith et al., (2007) | Hurler | 20 | 12:8 | 2.9  (0.5 – 5.1) | 2 | 3 | 2 | 3 | 45  (9) | 0.83 | - | - | - | - | - | - | - | - | - | - | - | - | - | - | - | - | - | - | - | - |
| Maas et al., (2008) | JS | 43 | 15:27 | 9y 10m  (1y7m – 25y 10m) | 0 | 0 | 1 | 1 | 2  (1) | 0.17 | - | - | - | - | 1 | 1 | 9  (4) | 0.17 | 1 | 2 | 44  (19) | 0.25 | 1 | 2 | 28  (12) | 0.25 | 0 | 1 | 28  (12) | 0.08 |
| Maas et al., (2012) | JS | 25 | 9:16 | 12.9 ± 5.5  (3.9-25.10) | 2 | 0 | - | - | - | - | - | - | - | - | - | - | - | - | - | - | - | - | - | - | - | - | 3 | 3 | 20  (5) | 0.67 |
| Kirveskari et al., (2000) | JNCL | 28 | - | 13  (6 – 27) | 0 | 3 | - | - | - | - | 2 | 3 | 46  (13) | 0.67 | 2 | 3 | 36  (10) | 0.67 | - | - | - | - | - | - | - | - | - | - | - | - |
| Malcolm et al., (2012) | JNCL | 9 | 4:5 | Median 5  (2.5 – 18.0) | 2 | 0 | - | - | - | - | - | - | - | - | - | - | - | - | - | - | - | - | - | - | - | - | 2 | 2 | 100  (8)^[[49]](#footnote-49)^ | 0.50 |
| Bax & Colville (1995) | MPSII | 48 | 48:0 | 8.6 ± 5.9  (0-30)^[[50]](#footnote-50)^ | 2 | 0 | - | - | - | - | - | - | - | - | - | - | - | - | - | - | - | - | - | - | - | - | 1 | 1 | 63  (30) | 0.33 |
| Eisengart et al., (2020) | MPSII | 9 | Not reported | -  (4-9) | 2 | 1 | - | - | - | - | - | - | - | - | - | - | - | - | - | - | - | - | - | - | - | - | 1 | 1 | 100  (9) | 0.42 |
| Gönüldaş et al., (2014) | MPSII | 5 | 43:33^[[51]](#footnote-51)^ | 10  (2-36)^[[52]](#footnote-52)^ | 0 | 0 | 2 | 3 | 100  (5) | 0.42 | - | - | - | - | - | - | - | - | - | - | - | - | - | - | - | - | - | - | - | - |
| Jimenez-Arredondo et al., (2017) | MPSII | 9 | 9:0 | 7y3m (3y2m-13y9m) | 1 | 3 | 0 | 1 | 67  (6) | 0.42 | - | - | - | - | - | - | - | - | - | - | - | - | - | - | - | - | - | - | - | - |
| Lin et al., (2010) | MPSII | 15 | 15:0 | 11.74  (3.7 – 23.7) | 1 | 3 | 2 | 3 | 100  (15) | 0.75 | - | - | - | - | - | - | - | - | - | - | - | - | - | - | - | - | - | - | - | - |
| Namazova-Baranova et al., (2013) | MPSII | 17 | 17:0 | 12.5 ± 3.9  (-) | 1 | 0 | 2 | 3 | 59  (10) | 0.50 | - | - | - | - | - | - | - | - | - | - | - | - | - | - | - | - | - | - | - | - |
| Okuyama et al., (2010) | MPSII | 9 | 9:0 | 30.1  (21.1 – 53.9) | 2 | 3 | 2 | 3 | 67  (6) | 0.83 | - | - | - | - | - | - | - | - | - | - | - | - | - | - | - | - | - | - | - | - |
| Suzuki et al., (2020) | MPSII | 109 | 108:1 | -^[[53]](#footnote-53)^ | 2 | 1 | 0 | 1 | 43 (47) | 0.33 | - | - | - | - | - | - | - | - | - | - | - | - | - | - | - | - | - | - | - | - |
| Wooten et al.,  (2013) | MPSII | 30 | 30:0 | Median 9  (3 – 25) | 3 | 3 | 2 | 3 | 90  (27) | 0.92 | - | - | - | - | - | - | - | - | - | - | - | - | - | - | - | - | - | - | - | - |
| Bax & Colville (1995) | MPSIIIB | 96 | 50:56^[[54]](#footnote-54)^ | 8.4 ± 4.2  (2-24)^[[55]](#footnote-55)^ | 2 | 0 | - | - | - | - | - | - | - | - | - | - | - | - | - | - | - | - | - | - | - | - | 1 | 1 | 86  (83) | 0.33 |
| Colville et al., (1996) | MPSIIIB | 80 | 40:40 | 10y2m ± 5.9  (4.4 – 25.6) | 2 | 0 | - | - | - | - | 2 | 3 | 48  (38) | 0.58 | - | - | - | - | - | - | - | - | - | - | - | - | 1 | 1 | 78  (62) | 0.33 |
| Fraser et al., (2005) | MPSIIIB | 141 | - | 13.5  (0-40) | 2 | 0 | - | - | - | - | - | - | - | - | - | - | - | - | - | - | - | - | - | - | - | - | 2 | 2 | 91  (129) | 0.50 |
| Gönüldaş et al., (2014) | MPSIIIB | 8 | 43:33^[[56]](#footnote-56)^ | 10  (2-36)^[[57]](#footnote-57)^ | 0 | 0 | 2 | 3 | 88  (7) | 0.42 | - | - | - | - | - | - | - | - | - | - | - | - | - | - | - | - | - | - | - | - |
| Lin et al., (2018) | MPSIIIB | 28 | 15:13 | 10.1 ± 5.7 | 2 | 3 | 2 | 3 | 11  (3) | 0.83 | - | - | - | - | - | - | - | - | - | - | - | - | - | - | - | - | 1 | 1 | 61 (17) | 0.58 |
| Mahon et al., (2014) | MPSIIIB | 8 | 5:3 | 9y3m  (2-15) | 2 | 3 | - | - | - | - | 2 | 3 | 63  (5) | 0.83 | - | - | - | - | - | - | - | - | - | - | - | - | - | - | - | - |
| Malcolm et al., (2012) | MPSIIIB | 15 | 8:7 | Median 9 (5-21) | 2 | 0 | - | - | - | - | - | - | - | - | - | - | - | - | - | - | - | - | - | - | - | - | 2 | 2 | 100 (13)^[[58]](#footnote-58)^ | 0.50 |
| Ruijter et al., (2008) | MPSIIIB | 12 | 5:7 | (3y 9m – 48y 8m) | 2 | 3 | - | - | - | - | 1 | 3 | 50  (6) | 0.75 | - | - | - | - | - | - | - | - | - | - | - | - | - | - | - | - |
| Bax & Colville (1995) | MPSIVA | 25 | 15:20^[[59]](#footnote-59)^ | 10.3 ± 7  (0-28)^[[60]](#footnote-60)^ | 2 | 0 | - | - | - | - | - | - | - | - | - | - | - | - | - | - | - | - | - | - | - | - | 1 | 1 | 44  (11) | 0.33 |
| Facchina et al., (2018) | MPSIVA | 16 | 7:9 | 10.5 ± 4.2 | 1 | 3 | 3 | 3 | 73 (11)^[[61]](#footnote-61)^ | 0.83 | - | - | - | - | - | - | - | - | - | - | - | - | - | - | - | - | - | - | - | - |
| Gönüldaş et al., (2014) | MPSIVA | 7 | 43:33^[[62]](#footnote-62)^ | 10  (2-36)^[[63]](#footnote-63)^ | 0 | 0 | 2 | 3 | 86  (6) | 0.42 | - | - | - | - | - | - | - | - | - | - | - | - | - | - | - | - | - | - | - | - |
| Kenth et al., (2019) | MPSIVA | 16 | 7:9 | 152.25m ± 40.73 | 1 | 3 | 2 | 2 | 69 (11) | 0.67 | - | - | - | - | - | - | - | - | - | - | - | - | - | - | - | - | - | - | - | - |
| Fjermestad et al., (2018) | NF | 142 | 64:88 | 50.3 ± 12.0 | 2 | 1 | - | - | - | - | 2 | 3 | 41  (58) | 0.75 | 1 | 1 | 46  (65) | 0.42 | - | - | - | - | - | - | - | - | - | - | - | - |
| Johnson et al., (2005) | NF | 64 | 39:25 | 10y7m ± 4y1m  (3-18) | 1 | 0 | - | - | - | - | - | - | - | - |  | - | - | - | 1 | 2 | 2  (1) | 0.33 | 1 | 2 | 3  (2) | 0.33 | 2 | 2 | 64  (41) | 0.42 |
| Leschnizer et al., (2013) | NF | 100 | 43:57 | 36.9  (16-69) | 2 | 1 | 1 | 1 | 7  (7) | 0.42 | 2 | 3 | 88  (88) | 0.67 | 1 | 2 | 20  (20) | 0.50 | - | - | - | - | - | - | - | - | - | - | - | - |
| Licis et al., (2013) | NF | 129 | 64:65 | 8.58  (2-7) | 2 | 1 | - | - | - | - | - | - | - | - | - | - | - | - | - | - | - | - | - | - | - | - | 3 | 3 | 53  (68) | 0.75 |
| Madubata et al., (2015) | NF | 8579 | 4093:4486 | 24.4 ± 19.3  (0-64)^[[64]](#footnote-64)^ | 3 | 1 | - | - | - | - | - | - | - | - | - | - | - | - | - | - | - | - | - | - | - | - | 1 | 1 | 4  (311) | 0.50 |
| Marana-Pérez et al., (2015) | NF | 95 | 51:44 | -  (3y10 m – 17y 10m) | 1 | 1 | 1 | 1 | 3  (3) | 0.33 | 1 | 3 | 11  (10) | 0.50 | 1 | 1 | 9  (9) | 0.33 | - | - | - | - | - | - | - | - | - | - | - | - |
| Abel & Tonnsen (2017) | PWS | 19 | 8:11 | Median 22m  (10-44) | 3 | 1 |  |  | - | - | - | - | - | - | - | - | - | - | - | - | - | - | - | - | - | - | 1 | 2 | 16  (3) | 0.58 |
| Abel et al., (2018) | PWS | 48 | 20:28 | Median 2.3 (0.2-14.1) | 1 | 0 | 3 | 3 | 42 (20) | 0.58 | - | - | - | - | - | - | - | - | - | - | - | - | - | - | - | - | - | - | - | - |
| Al-Saleh et al., (2013) | PWS | 15 | 11:4 | 3.7  (0.8-15.4) | 1 | 3 | 2 | 3 | 33  (5) | 0.75 | - | - | - | - | - | - | - | - | - | - | - | - | - | - | - | - | - | - | - | - |
| Arens et al., (1994) | PWS | 13 | 6:7 | 23.4 ± 3.7  (-) | 0 | 3 | 2 | 2 | 0  (0) | 0.58 | - | - | - | - | - | - | - | - | - | - | - | - | - | - | - | - | - | - | - | - |
| Arens et al., (1996) | PWS | 17 | 7:10 | -^[[65]](#footnote-65)^ | 0 | 3 | 2 | 2 | 7  (1)^[[66]](#footnote-66)^ | 0.58 | - | - | - | - | - | - | - | - | - | - | - | - | - | - | - | - | - | - | - | - |
| Beauloye et al., (2015) | PWS | 20 | 9:11 | 5.9  (0.8-14.44) | 2 | 3 | 3 | 3 | 27  (3)^[[67]](#footnote-67)^ | 0.92 | - | - | - | - | - | - | - | - | - | - | - | - | - | - | - | - | - | - | - | - |
| Berini et al., (2013) | PWS | 50 | 24:26 | Median 1.9  (-) | 2 | 3 | 3 | 3 | 6  (3) | 0.92 | - | - | - | - | - | - | - | - | - | - | - | - | - | - | - | - | - | - | - | - |
| Boer & Clarke (1999) | PWS | 203 | - | Males: 17.1 (3-51)  Females: 16.4 (3 – 49) | 2 | 1 | - | - | - | - | 1 | 3 | 31  (62) | 0.58 | 0 | 1 | 2  (5) | 0.33 | - | - | - | - | - | - | - | - | - | - | - | - |
| Butler et al., (2002) | PWS | 66 | 40:26 | 19  (0-46) | 3 | 3 | 0 | 1 | 18  (12) | 0.58 | 1 | 1 | 63%  (40) | 0.67 | 0 | 1 | 73  (47) | 0.58 | - | - | - | - | - | - | - | - | - | - | - | - |
| Canora et al., (2018) | PWS | 14 | 11:3 | 7.1 ± 6.1 | 0 | 3 | 2 | 3 | 93 (13) | 0.67 | - | - | - | - | - | - | - | - | - | - | - | - | - | - | - | - | - | - | - | - |
| Clarke et al., (1989) | PWS | 60 | 31:29 | 23  (16-43) | 2 | 3 | - | - | - | - | - | - | - | - | 0 | 1 | 63  (38) | 0.50 | - | - | - | - | - | - | - | - | - | - | - | - |
| Clift et al., (1994) | PWS | 17 | 9:8 | 21  (11-39) | 2 | 3 | 1 | 1 | 94  (16) | 0.58 | - | - | - | - | 2 | 2 | 50  (7) | 0.75 | 0 | 1 | 12  (2) | 0.50 | - | - | - | - | - | - | - | - |
| Cohen et al., (2014) | PWS | 44 | - | Median 1.9  (0.3-15.6) | 1 | 0 | 2 | 3 | 57  (25) | 0.50 | - | - | - | - | - | - | - | - | - | - | - | - | - | - | - | - | - | - | - | - |
| Cotton & Richdale (2006) | PWS | 16 | 13:4 | 11.59 (3-18) | 1 | 0 | - | - | - | - | 1 | 3 | 19  (3) | 0.42 | 1 | 1 | 31  (5) | 0.25 | 1 | 2 | 0  (0) | 0.33 | - | - | - | - | 1 | 1 | 44  (7) | 0.25 |
| Donze et al., (2019) | PWS | 28 | 8:19 | 17.2 ± 1.8 | 0 | 3 | 3 | 3 | 0  (0) | 0.75 |  |  |  |  |  |  |  |  |  |  |  |  |  |  |  |  |  |  |  |  |
| Festen et al., (2006) | PWS | 53 | 30:23 | Median 5.4 (2.1-7.2)^[[68]](#footnote-68)^ | 2 | 3 | 3 | 3 | 15  (8) | 0.92 | - | - | - | - | - | - | - | - | - | - | - | - | - | - | - | - | - | - | - | - |
| Festen et al., (2007) | PWS | 22 | 15:7 | Median 1.8  (1.1-34)^[[69]](#footnote-69)^ | 2 | 3 | 3 | 3 | 100  (22) | 0.92 | - | - | - | - | - | - | - | - | - | - | - | - | - | - | - | - | - | - | - | - |
| Festen et al., (2008) | PWS | 31 | 14:16 | Median 6.4  (6.0-9.1)^[[70]](#footnote-70)^ | 2 | 3 | 3 | 3 | 100  (31) | 0.92 | - | - | - | - | 2 | 1 | 16  (5) | 0.67 | - | - | - | - | - | - | - | - | - | - | - | - |
| Fillion et al., (2009) | PWS | 10 | 5:5 | 8.6  (1.3-13.5) | 1 | 3 | 0 | 1 | 10  (1) | 0.42 | - | - | - | - | - | - | - | - | - | - | - | - | - | - | - | - | - | - | - | - |
| Ghergan et al., (2017) | PWS | 60 | 26:34 | 25 ± 10 | 2 | 3 | 3 | 3 | 23  (14) | 0.92 | - | - | - | - | 2 | 2 | 20 (12) | 0.75 | 2 | 2 | 10  (6) | 0.75 | - | - | - | - | - | - | - | - |
| Gunay-Aygun et al., (2001) | PWS | 101 | - | Median 14.5  (5m – 60y) | 1 | 3 | - | - | - | - | - | - | - | - | - | - | - | - | - | - | - | - | - | - | - | - | 1 | 1 | 37  (25)^[[71]](#footnote-71)^ | 0.50 |
| Harris & Allen (1996) | PWS | 8 | 5:3 | 15.18  (5.5-21) | 0 | 3 | 2 | 2 | 88  (7) | 0.58 | - | - | - | - | - | - | - | - | - | - | - | - | - | - | - | - | - | - | - | - |
| Hedgeman et al., (2017) | PWS | 155 | 71:84 | 18 ± 17 | 3 | 1 | 1 | 1 | 0  (0) | 0.58 | - | - | - | - | - | - | - | - | - | - | - | - | - | - | - | - | - | - | - | - |
| Helbing-Zwanenburg et al., (1993) | PWS | 21 | 13:8 | 30  (12-54) | 0 | 1 | 2 | 2 | 0  (0)^[[72]](#footnote-72)^ | 0.42 | - | - | - | - | 1 | 1 | 86  (18) | 0.25 | - | - | - | - | - | - | - | - | - | - | - | - |
| Khayat et al., (2017) | PWS | 28 | 12:14 | Median 0.9  (0.2-2.0) | 1 | 3 | 2 | 3 | 11  (3) | 0.75 | - | - | - | - | - | - | - | - | - | - | - | - | - | - | - | - | - | - | - | - |
| Lan et al., (2016) | PWS | 9 | 4:5 | 8.72 ± 4.21  (3-15) | 0 | 0 | 3 | 3 | 100  (9) | 0.50 | - | - | - | - | - | - | - | - | - | - | - | - | - | - | - | - | - | - | - | - |
| Laurier et al., (2015) | PWS | 153 | 68:86 | 28.4  (16-54) | 2 | 3 | 0 | 2 | 35  (53) | 0.58 | - | - | - | - | - | - | - | - | - | - | - | - | - | - | - | - | - | - | - | - |
| Lecka-Ambroziak et al., (2017) | PWS | 11 | 5:6 | 3 | 0 | 3 | 2 | 3 | 82  (9) | 0.67 | - | - | - | - | - | - | - | - | - | - | - | - | - | - | - | - | - | - | - | - |
| Lin et al., (2007) | PWS | 30 | 16:14 | 7.4 ± 4.1  (1-19) | 2 | 3 | 2 | 3 | 93  (28) | 0.83 | - | - | - | - | - | - | - | - | - | - | - | - | - | - | - | - | - | - | - | - |
| Maas et al., (2009b) | PWS | 7 | 4:3 | 39.86  (33-49) | 2 | 3 | - | - | - | - | - | - | - | - | 2 | 2 | 100  (7) | 0.75 | - | - | - | - | - | - | - | - | - | - | - | - |
| Maas et al., (2010) | PWS | 79 | 34:45 | 34.4  (18-65) | 2 | 3 | 0 | 2 | 4  (3) | 0.58 | 2 | 3 | 14  (11) | 0.83 | 1 | 1 | 33  (26) | 0.42 | - | - | - | - | - | - | - | - | - | - | - | - |
| Manni et al., (2001) | PWS | 14 | 6:8 | 17  (8-37) | 2 | 3 | 2 | 3 | 29  (4) | 0.83 | - | - | - | - | 2 | 2 | 44  (8) | 0.75 | - | - | - | - | - | - | - | - | - | - | - | - |
| Meyer et al., (2012) | PWS | 13 | 7:6 | 3  (6m– 11y) | 1 | 3 | 2 | 3 | 69  (9) | 0.75 | - | - | - | - | - | - | - | - | - | - | - | - | - | - | - | - | - | - | - | - |
| Miller et al., (2006) | PWS | 25 | 15:10 | 13  (6m – 39y) | 0 | 3 | 2 | 2 | 100  (25) | 0.58 | - | - | - | - | - | - | - | - | - | - | - | - | - | - | - | - | - | - | - | - |
| Miller et al., (2009) | PWS | 20 | 12:8 | 8.05  (2-21m) | 0 | 3 | 2 | 2 | 70  (14) | 0.58 | - | - | - | - | - | - | - | - | - | - | - | - | - | - | - | - | - | - | - | - |
| O’Donoghue et al., (2005) | PWS | 13 | 5:8 | 14.6  (1.5 - 28) | 3 | 1 | 2 | 2 | 69  (9) | 0.67 | - | - | - | - | - | - | - | - | - | - | - | - | - | - | - | - | - | - | - | - |
| Partsch et al., (2000) | PWS | 19 | 7:12 | 23  (18-34) | 0 | 3 | 0 | 1 | 58  (11) | 0.33 | - | - | - | - | - | - | - | - | - | - | - | - | - | - | - | - | - | - | - | - |
| Pavone et al., (2015) | PWS | 88 | 44:44 | Median 5.1  (0.3–44.3) | 1 | 3 | 3 | 3 | 50  (44) | 0.83 | - | - | - | - | - | - | - | - | - | - | - | - | - | - | - | - | - | - | - | - |
| Priano et al., (2006) | PWS | 18 | 11:7 | 27.5 ± 5.5  (18.1 – 40.1) | 0 | 3 | 2 | 2 | 33  (6) | 0.58 | - | - | - | - | 2 | 2 | 80  (8) | 0.58 | - | - | - | - | - | - | - | - | - | - | - | - |
| Proffit etal., (2018) | PWS | 2029 | 934:1000^[[73]](#footnote-73)^ | 22.4  (0-84) | 2 | 1 | 1 | 1 | 45 (905) | 0.42 | - | - | - | - | - | - | - | - | - | - | - | - | - | - | - | - | - | - | - | - |
| -Richards et al., (1994) | PWS | 14 | 9:5 | 21  (16-39) | 0 | 0 | 2 | 2 | 86  (12) | 0.33 | - | - | - | - | 1 | 2 | 22  (4) | 0.25 | - | - | - | - | - | - | - | - | - | - | - | - |
| Richdale et al., (1999) | PWS | 29 | 19:10 | 14.4 ± 11.4  (6m – 46y) | 2 | 0 | - | - | - | - | 2 | 3 | 17  (5) | 0.58 | 1 | 2 | 34  (10) | 0.42 | 1 | 2 | 3  (1) | 0.33 | - | - | - | - | - | - | - | - |
| Saeves et al., (2018) | PWS | 29 | 12:17 | -^[[74]](#footnote-74)^ | 1 | 3 | 1 | 1 | 31  (9) | 0.50 | - | - | - | - | - | - | - | - | - | - | - | - | - | - | - | - | - | - | - | - |
| Salehi et al., (2017) | PWS | 10 | 6:4 | 9.7 ± 8.4m | 1 | 3 | 0 | 2 | 70  (7) | 0.50 | - | - | - | - | - | - | - | - | - | - | - | - | - | - | - | - | - | - | - | - |
| Sanjeeva et al., (2017) | PWS | 34 | 23:11 | Median 8  (1-24) | 1 | 3 | 1 | 1 | 68  (23) | 0.50 | - | - | - | - | 0 | 1 | (20) | 0.42 | - | - | - | - | - | - | - | - | - | - | - | - |
| Scheermeyer et al., (2017) | PWS | 73 | 37:36 | -^[[75]](#footnote-75)^ | 2 | 3 | 2 | 2 | 40  (29) | 0.75 | - | - | - | - | - | - | - | - | - | - | - | - | - | - | - | - | - | - | - | - |
| Schlüter et al., (1996) | PWS | 8 | 5:3 | -  (44 days – 12.5 y) | 1 | 33 | 2 | 2 | 63  (5) | 0.67 | - | - | - | - | - | - | - | - | - | - | - | - | - | - | - | - | - | - | - | - |
| Schrander-Stumpel et al., (2007) | PWS | 14 | 37:37 | 31.7  (18-63) | 2 | 3 | 0 | 1 | 29  (4)^[[76]](#footnote-76)^ | 0.50 | - | - | - | - | - | - | - | - | - | - | - | - | - | - | - | - | - | - | - | - |
| Torrado et al., (2007) | PWS | 101 | 49:42 | 4.09  (12 days – 17 y) | 1 | 3 | 3 | 3 | 29  (10)^[[77]](#footnote-77)^ | 0.83 | - | - | - | - | - | - | - | - | - | - | - | - | - | - | - | - | 1 | 1 | 37  (29)^[[78]](#footnote-78)^ | 0.50 |
| van Wijngaarden et al., (2009) | PWS | 20 | 11:9 | Median 6  (4.2 – 9.2) | 0 | 3 | 3 | 3 | 100  (20) | 0.75 | - | - | - | - | - | - | - | - | - | - | - | - | - | - | - | - | - | - | - | - |
| Vandeleur et al., (2013) | PWS | 34 | 17:17 | 7.3  (3m – 16.3y) | 1 | 3 | 3 | 3 | 44  (15) | 0.83 | - | - | - | - | - | - | - | - | - | - | - | - | - | - | - | - | - | - | - | - |
| Verrillo et al., (2009) | PWS | 30 | 17:13 | 6.1  (-) | 2 | 3 | 2 | 2 | 50  (15) | 0.75 | - | - | - | - | - | - | - | - | - | - | - | - | - | - | - | - | - | - | - | - |
| Vgontzas et al., (1996) | PWS | 8 | 1:7 | 21  (6-40) | 1 | 2 | 2 | 2 | 0  (0) | 0.58 | - | - | - | - | 1 | 1 | 38  (3) | 0.42 | - | - | - | - | - | - | - | - | - | - | - | - |
| Viardot et al., (2010) | PWS | 12 | 8:4 | 27.9  (-) | 3 | 3 | 1 | 1 | 42  (5) | 0.67 | - | - | - | - | - | - | - | - | - | - | - | - | - | - | - | - | - | - | - | - |
| Anderson et al., (2014) | Rett | 423 | 0:423 | Median 25  (18-54) | 3 | 1 | - | - | - | - | - | - | - | - | - | - | - | - | - | - | - | - | - | - | - | - | 1 | 1 | 63  (246) | 0.50 |
| Boban et al., (2016) | Rett | 364 | 0:364 | Median 14.5  (2.1-57.2) | 1 | 3 | - | - | - | - | 2 | 2 | 39  (141)^[[79]](#footnote-79)^ | 0.67 | 1 | 1 | 13  (46) | 0.50 | - | - | - | - | 1 | 1 | 28  (101)^[[80]](#footnote-80)^ | 0.50 | - | - | - | - |
| Boban et al., (2018) | Rett | 364 | 2:362 | Median 14y4m (2-57y) | 2 | 3 | - | - | - | - | 3 | 3 | 93  (340) | 0.92 | - | - | - | - | - | - | - | - | - | - | - | - | - | - | - | - |
| Fehr et al., (2013) | Rett | 766 | 0:766 | 10.5  (1.3 – 54.2) | 1 | 3 | - | - | - | - | - | - | - | - | - | - | - | - | - | - | - | - | - | - | - | - | 1 | 1 | 67  (513) | 0.50 |
| Glaze et al., (1987) | Rett | 11 | 0:11 | -  (2-15) | 0 | 1 | 2 | 3 | 9  (1) | 0.50 | - | - | - | - | - | - | - | - | - | - | - | - | - | - | - | - | - | - | - | - |
| Hagebeuk et al., (2012) | Rett | 12 | 0:12 | 9.83  (3-33) | 1 | 1 | 3 | 3 | 50  (6) | 0.67 | - | - | - | - | - | - | - | - | - | - | - | - | - | - | - | - | - | - | - | - |
| Halbach et al., (2008) | Rett | 53 | 0:53 | 26.9  (16-53) | 1 | 2 | - | - | - | - | 2 | 3 | 51  (27) | 0.67 | 1 | 1 | 85  (45) | 0.42 | - | - | - | - | - | - | - | - | - | - | - | - |
| Hara et al., (2014) | Rett | 22 | 0:22 | 16.44 ± 8.56  (4 – 37.5) | 0 | 3 | - | - | - | - | - | - | - | - | - | - | - | - | - | - | - | - | - | - | - | - | 1 | 1 | 82  (18) | 0.42 |
| Killian et al., (2018) | Rett | 113 | 0:113 | 19 aged ≥13 | 2 | 3 | - | - | - | - | - | - | - | - | - | - | - | - | - | - | - | - | - | - | - | - | 0 | 0 | 50  (57) | 0.42 |
| Mangatt et al., (2016) | Rett | 591 | 0:591 | Median 13  (2.0-24.6)^[[81]](#footnote-81)^ | 2 | 3 | - | - | - | - | 2 | 1 | 71  (422) | 0.67 | - | - | - | - | - | - | - | - | - | - | - | - | 1 | 1 | 87  (122)^[[82]](#footnote-82)^ | 0.58 |
| Marcus et al., (1994) | Rett | 30 | 0:30 | Median 7  (1 -32 years) | 0 | 1 | 2 | 2 | 3  (1) | 0.42 | - | - | - | - | - | - | - | - | - | - | - | - | - | - | - | - | - | - | - | - |
| Marschik et al., (2018) | Rett | 6 | 0:6 | 19.4m | 3 | 1 | - | - | - | - | - | - | - | - | - | - | - | - | - | - | - | - | - | - | - | - | 1 | 1 | 0  (0) | 0.5 |
| Merbler et al., (2018) | Rett | 13 | 0:13 | 9y5m (1y8m-17y1m) | 1 | 3 | - | - | - | - | - | - | - | - | - | - | - | - | - | - | - | - | 1 | 2 | 44  (4)^[[83]](#footnote-83)^ | 0.58 | 3 | 3 | 55  (5)^[[84]](#footnote-84)^ | 0.83 |
| Mori et al., (2019) | Rett | 168 | 0:168 | -^[[85]](#footnote-85)^ | 3 | 0 | - | - | - | - | 2 | 1 | 86  (144) | 0.50 | - | - | - | - | - | - | - | - | - | - | - | - | - | - | - | - |
| Percy et al., (1987) | Rett | 18 | 0:18 | - | 0 | 1 | 0 | 1 | 6  (1) | 0.17 | - | - | - | - | - | - | - | - | - | - | - | - | - | - | - | - | - | - | - | - |
| Piazza et al., (1990) | Rett | 20 | 0:20 | 9  (1-32) | 1 | 1 | - | - | - | - | 2 | 1 | 85  (17) | 0.42 | - | - | - | - | - | - | - | - | - | - | - | - | - | - | - | - |
| Wong et al., (2015) | Rett | 217 | 0:320 | Median 13  (8.3 – 17.5)^[[86]](#footnote-86)^ | 2 | 1 | - | - | - | - | 2 | 1 | 54  (117) | 0.50 | - | - | - | - | - | - | - | - | - | - | - | - | 1 | 1 | 85  (170)^[[87]](#footnote-87)^ | 0.42 |
| Young et al., (2007) | Rett | 237 | 0:237 | -  (2-29) | 2 | 1 | - | - | - | - | 2 | 1 | 46  (34)^[[88]](#footnote-88)^ | 0.50 | 1 | 1 | 77  (156)^[[89]](#footnote-89)^ | 0.42 | - | - | - | - | 1 | 1 | 55 (111)^[[90]](#footnote-90)^ | 0.42 | 1 | 1 | 81  (152)^[[91]](#footnote-91)^ | 0.42 |
| Zappella et al., (1990) | Rett | 12 | 0:12 | -  (3-14) | 0 | 1 | - | - | - | - | 2 | 1 | 42  (5) | 0.30 | - | - | - | - | - | - | - | - | - | - | - | - | - | - | - | - |
| Freeman et al., (2016) | SLOS | 20 | 9:11 | 8.6  (1-18) | 0 | 3 | - | - | - | - | - | - | - | - | - | - | - | - | - | - | - | - | - | - | - | - | 3 | 3 | 70  (14) | 0.75 |
| Zarowski et al., (2011) | SLOS | 18 | 7:11 | Median 10.7 ± 8.5 years  (-) | 1 | 1 | 1 | 1 | 50  (9) | 0.33 | 1 | 3 | 56  (10) | 0.50 | 1 | 1 | 44  (8) | 0.33 | 1 | 2 | 78  (14) | 0.42 | 1 | 2 | 39  (7) | 0.42 | - | - | - | - |
| Barboni et al., (2008) | SMS | 5 | 2:3 | 13 ± 8 | 1 | 3 | - | - | - | - | - | - | - | - | - | - | - | - | - | - | - | - | - | - | - | - | 1 | 1 | 60  (3) | 0.50 |
| Boddaert et al., (2004) | SMS | 5 | 5:0 | 13.3  (11.5 – 16.5) | 0 | 3 | - | - | - | - | - | - | - | - | - | - | - | - | - | - | - | - | - | - | - | - | 1 | 0 | 100  (5) | 0.33 |
| De Leersnyder et al., (2001) | SMS | 20 | 9:11 | 9.5  (4-17) | 0 | 3 | - | - | - | - | - | - | - | - | - | - | - | - | - | - | - | - | - | - | - | - | 2 | 1 | 100  (20) | 0.50 |
| Greenberg et al., (1991) | SMS | 23 | 14:18 | 15  (1m - 72 y) | 2 | 3 | - | - | - | - | 1 | 1 | 65  (15) | 0.58 | - | - | - | - | - | - | - | - | - | - | - | - | - | - | - | - |
| Loviglio et al., (2016) | SMS | 10 | - | - | 2 | 3 | - | - | - | - | - | - | - | - | - | - | - | - | - | - | - | - | - | - | - | - | 1 | 1 | 80  (8) | 0.58 |
| Potocki et al., (2000) | SMS | 26 | 18:21 | 10.5  (1.6-32) | 3 | 3 | - | - | - | - | - | - | - | - | 1 | 2 | 50  (13) | 0.75 | - | - | - | - | - | - | - | - | - | - | - | - |
| Smith et al., (1998b) | SMS | 39 | 18:21 | 10.5  (1.6-32) | 2 | 1 | 1 | 1 | 23  (9) | 0.42 | 1 | 3 | 31  (12) | 0.58 | 1 | 1 | 64  (25) | 0.42 | 1 | 2 | 79  (31) | 0.50 | 1 | 2 | 44  (17) | 0.50 | - | - | - | - |
| Trickett et al., (2018) | SMS | 26 | 16:10 | 8.54 ± 3.08 | 2 | 1 | - | - | - | - | 2 | 3 | 81 (21) | 0.67 | - | - | - | - | - | - | - | - | - | - | - | - | - | - | - | - |
| Bruni et al., (1995) | TSC | 10 | 1:9 | 11  (2-17.1) | 1 | 2 | - | - | - | - | 1 | 1 | 80  (8) | 0.42 | 1 | 1 | 60  (6) | 0.42 | - | - | - | - | - | - | - | - | - | - | - | - |
| De Vries et al., (2018) | TSC | 2216 | 1062:1154 | Median 13 (1-71) | 3 | 3 | - | - | - | - | - | - | - | - | - | - | - | - | - | - | - | - | - | - | - | - | 1 | 1 | 44  (331)^[[92]](#footnote-92)^ | 0.67 |
| Ho et al., (2018) | TSC | 150 | 78:64^[[93]](#footnote-93)^ | Median 13.5  (7-23) | 2 | 1 | - | - | - | - | - | - | - | - | - | - | - | - | - | - | - | - | - | - | - | - | 1 | 1 | 60 (88)^[[94]](#footnote-94)^ | 0.42 |
| Hunt (1993) | TSC | 232 | 162:70 | - ^[[95]](#footnote-95)^  (6m – 74y) | 2 | 0 | - | - | - | - | 1 | 1 | 62  (143) | 0.33 | - | - | - | - | - | - | - | - | - | - | - | - | - | - | - | - |
| Hunt & Stores (1994) | TSC | 40 | 22:18 | -  (2 – 15) | 2 | 0 | - | - | - | - | 3 | 3 | 53  (21) | 0.67 | - | - | - | - | - | - | - | - | - | - | - | - | - | - | - | - |
| Trickett et al., (2018) | TSC | 20 | 11:9 | 7.20 ± 4.11 | 2 | 1 | - | - | - | - | 2 | 3 | 45  (9) | 0.67 | - | - | - | - | - | - | - | - | - | - | - | - | - | - | - | - |
| Vignoli et al., (2015) | TSC | 42 | 18:24 | Median 19.3  (4-44) | 1 | 3 | - | - | - | - | - | - | - | - | - | - | - | - | - | - | - | - | - | - | - | - | 1 | 0 | 21  (9) | 0.42 |
| Van Eeghen et al., (2011) | TSC | 36 | 10:26 | 33.5  (-) | 1 | 1 | 2 | 1 | 6  (2) | 0.42 | 2 | 3 | 22  (8) | 0.58 | 2 | 1 | 42  (15) | 0.42 | - | - | - | - | - | - | - | - | - | - | - | - |
| Abel & Tonnsen (2017) | WS | 19 | 11:8 | 20m  (6-46) | 3 | 1 | - | - | - | - | - | - | - | - | - | - | - | - | - | - | - | - | - | - | - | - | 1 | 2 | 58 (11) | 0.58 |
| Annaz et al., (2011) | WS | 64 | 28:36 | 8.3 ± 2.07  (6.02 – 12.06) | 2 | 3 | - | - | - | - | 2 | 3 | 97  (62) | 0.83 | 1 | 1 | 61  (39) | 0.58 | 0 | 2 | 52  (33) | 0.58 | 0 | 2 | 11  (7) | 0.58 | - | - | - | - |
| Arens et al., (1998) | WS | 28 | - | 4.7  (1.5 – 10) | 1 | 2 | 2 | 1 | 93  (26) | 0.50 | - | - | - | - | - | - | - | - | - | - | - | - | - | - | - | - | - | - | - | - |
| Ashworth et al., (2013) | WS | 24 | 12:12 | 9.55  (6.08-12.58) | 2 | 3 | 1 | 1 | 29  (7) | 0.58 | 1 | 3 | 54  (13) | 0.75 | 1 | 1 | 58  (14) | 0.58 | 1 | 2 | 46  (11) | 0.67 | 1 | 2 | 8  (2) | 0.67 | 1 | 1 | 17  (4) | 0.58 |
| Axelsson et al., (2013) | WS | 18 | 4:14 | 30.43m  (15.40 – 4.20) | 2 | 3 | - | - | - | - | - | - | - | - | - | - | - | - | - | - | - | - | - | - | - | - | 3 | 3 | 29  (4)^[[96]](#footnote-96)^ | 0.92 |
| Einfeld et al., (1997) | WS | 70 | 36:34 | 9.2  (-) | 2 | 2 | - | - | - | - | - | - | - | - | - | - | - | - | - | - | - | - | - | - | - | - | 1 | 2 | 31  (22) | 0.58 |
| Goldman et al., (2009) | WS | 23 | 12:11 | 25.5  (17-35) | 1 | 0 | 1 | 2 | 22  (5) | 0.33 | - | - | - | - | 1 | 2 | 35  (8) | 0.33 | - | - | - | - | - | - | - | - | 1 | 1 | 35  (8) | 0.25 |
| Kirchner et al., (2016) | WS | 16 | 5:11 | 28.7m ± 19.3  (3m – 5y) | 1 | 0 | - | - | - | - | - | - | - | - | - | - | - | - | - | - | - | - | - | - | - | - | 1 | 1 | 31  (5) | 0.25 |
| Mason et al., (2011) | WS | 35 | 15:20 | 9.34 ± 4.89  (-) | 1 | 3 | 2 | 2 | 26  (9) | 0.67 | 2 | 3 | 43  (15) | 0.75 | 1 | 1 | 14  (5) | 0.50 | - | - | - | - | - | - | - | - | - | - | - | - |
| Sammour et al., (2017) | WS | 87 | 46:41 | 9 ± 4.2  (3-19) | 2 | 3 | - | - | - | - | - | - | - | - | - | - | - | - | 0 | 2 | 56  (49) | 0.58 | - | - | - | - | - | - | - | - |
| Santoro et al., (2016) | WS | 25 | 15:10 | 12.1  (6-17) | 2 | 3 | 2 | 1 | 40  (10) | 0.67 | 2 | 3 | 20  (5) | 0.83 | 1 | 1 | 0  (0) | 0.58 | - | - | - | - | - | - | - | - | - | - | - | - |
| Sniecinska-Cooper et al., (2015) | WS | 25 | 12:13 | 7.31  (4-11) | 2 | 3 | - | - | - | - | - | - | - | - | - | - | - | - | - | - | - | - | - | - | - | - | 2 | 2 | 64  (16) | 0.75 |
| Von Gontard et al., (2016) | WS | 231 | 120:111 | 19.4 ± 11.61 (4.1-59.9) | 1 | 0 | - | - | - | - | - | - | - | - | - | - | - | - | 2 | 3 | 18  (39)^[[97]](#footnote-97)^ | 0.50 | - | - | - | - | - | - | - | - |

1. Only 62 participants of the total sample (n=91) completed the measure of ‘general’ sleep difficulties. [↑](#footnote-ref-1)
2. Only 72 pa rticipants of the total sample (n=73) completed the measure of ‘general’ sleep difficulties. [↑](#footnote-ref-2)
3. The authors report 50% of the sample were male, so the gender of one participant is presumed not to have been reported. [↑](#footnote-ref-3)
4. Mean age in each group - Deletion: 5.07, IC defects: 5.39, UPD: 5.83. [↑](#footnote-ref-4)
5. Only 21 participants of the total sample (n=27) completed the measure ‘general’ sleep difficulties. [↑](#footnote-ref-5)
6. Gender was not reported for two participants. [↑](#footnote-ref-6)
7. Only 142 participants of the total sample (n=153) completed the measure of sleep enuresis. [↑](#footnote-ref-7)
8. Only 44 participants of the total sample (n=45) completed the measure of sleep-related breathing difficulties. [↑](#footnote-ref-8)
9. Only available for a subset of the sample (2926 individuals on 1^st^ January 2013). [↑](#footnote-ref-9)
10. Only available for a subset of the sample (2926 individuals on 1^st^ January 2013). [↑](#footnote-ref-10)
11. Only available for a wider dataset which includes individuals who did not undergo sleep studies (n=303). [↑](#footnote-ref-11)
12. Only available for a wider dataset which includes individuals who did not undergo sleep studies (n=303). [↑](#footnote-ref-12)
13. Majority of participants were 2-4 years (57.5%). 20% were 6 to 24 months,15% 4-6 years and 7.5% participants were above 6 years. [↑](#footnote-ref-13)
14. Only 31 participants of the total sample (n=38) completed the measure of sleep-related breathing difficulties. [↑](#footnote-ref-14)
15. Mean age in the OSA group = 28.8 years (18.1-62.4), mean age in the no-OSA group = 23.2 years (18.5 – 35.8). [↑](#footnote-ref-15)
16. Only 28 participants of the total sample (n=31) completed the measure of ‘general’ sleep difficulties. [↑](#footnote-ref-16)
17. Only 40 participants of the total sample (n=47) completed the measure of excessive daytime sleepiness. [↑](#footnote-ref-17)
18. Only 40 participants of the total sample (n=47) completed the measure of ‘general’ sleep difficulties. [↑](#footnote-ref-18)
19. Only 94 participants of the total sample (n=193) completed the measure of sleep-related breathing difficulties. [↑](#footnote-ref-19)
20. Mean age of OSA group = 33.9 ± 22.3 months, mean age of non-OSA group = 36.3 ± 20.8 months. [↑](#footnote-ref-20)
21. 55 participants aged 7-11, 52 participants aged 12-18. [↑](#footnote-ref-21)
22. Only 52 participants of the total sample (n=63) completed the measure of sleep-related breathing difficulties. [↑](#footnote-ref-22)
23. 2682 participants aged 2-4, 4188 participants aged 5-20. [↑](#footnote-ref-23)
24. Only 21 participants of the total sample (n=74) completed the measure of sleep-related breathing difficulties. [↑](#footnote-ref-24)
25. Based on recruitment criteria. [↑](#footnote-ref-25)
26. Only available for a wider dataset which includes individuals who were studied because of symptom history (n=122). [↑](#footnote-ref-26)
27. 25^th^-75^th^ percentile. [↑](#footnote-ref-27)
28. Only available for a wider dataset which includes individuals who were studied because of symptom history (n=122). [↑](#footnote-ref-28)
29. Only 42 participants of the total sample (n=54) completed the measure of sleep related breathing difficulties. [↑](#footnote-ref-29)
30. 32 participants aged under 10, 28 participants aged 10 and over. [↑](#footnote-ref-30)
31. 8 participants aged 4-5 years, 17 participants aged 6-10 years, 11 participants aged 11-15 years. [↑](#footnote-ref-31)
32. Only 303 participants of the total sample (n=317) completed the measure of sleep enuresis. [↑](#footnote-ref-32)
33. An additional 34 participants did not respond to this question. [↑](#footnote-ref-33)
34. Mean age of OSA group 10.2 years, mean age of non-OSA group 7.8 years. [↑](#footnote-ref-34)
35. Only 97 participants of the total sample (n=100) completed the measure of sleep-related breathing difficulties. [↑](#footnote-ref-35)
36. 3 participants aged 1–12 months, 24 participants aged 1–4 years, 93 participants aged 5–12 years, 44 participants aged 13–18 years, 31 participants aged 19–30 years, 13 participants aged 31–40 years, 15 participants aged >40 years. [↑](#footnote-ref-36)
37. Only available for a wider dataset which includes individuals with intellectual disability who did not have Down syndrome (n=811). [↑](#footnote-ref-37)
38. Only available for a wider dataset which includes individuals with intellectual disability who did not have Down syndrome (n=811). [↑](#footnote-ref-38)
39. 28 participants aged 6-11 months, 18 participants aged 12-17 months, 21 participants aged 18-23 months, 37 participants aged 24-36 months. [↑](#footnote-ref-39)
40. Only 103 participants of the total sample (n=104) completed the measure of ‘general’ sleep difficulties. [↑](#footnote-ref-40)
41. 216 participants aged 3-11years, 112 participants aged 12-21. [↑](#footnote-ref-41)
42. 37 participants aged 3-6 years, 38 participants aged 7-11 years, and 15 participants aged 12-17 years. [↑](#footnote-ref-42)
43. Only 413 participants of the total sample (n=1295) completed the measure of excessive daytime sleepiness. [↑](#footnote-ref-43)
44. Mean age of 6 participants with mutation: 17.95, mean age of 6 participants without mutation: 19.25. [↑](#footnote-ref-44)
45. Only available for a wider dataset which includes individuals over the age of 14, whose sleep data was not considered (n=63). [↑](#footnote-ref-45)
46. Only available for a wider dataset which includes individuals over the age of 14, whose sleep data was not considered (n=63). [↑](#footnote-ref-46)
47. Only 6 of the total sample (n=9) completed the measure of sleep-related breathing difficulties. [↑](#footnote-ref-47)
48. Only 53 of the total sample (n=61) completed the measure of sleep-related breathing difficulties. [↑](#footnote-ref-48)
49. Only 8 participants of the total sample (n=9) completed the measure of ‘general’ sleep difficulties. [↑](#footnote-ref-49)
50. Only available for a wider dataset which includes individuals over the age of 14, whose sleep data was not considered (n=54). [↑](#footnote-ref-50)
51. Only available for a wider dataset which includes individuals who had other mucopolysaccharide disorders (n=76). See later reference to Gönüldaş et al., (2014). [↑](#footnote-ref-51)
52. Only available for a wider dataset which includes individuals who had other mucopolysaccharide disorders (n=76). See later reference to Gönüldaş et al., (2014). [↑](#footnote-ref-52)
53. Mean age in the severe group 10.85 ± 5.86, mean age in the attenuated group 16.69 ± 9.02. [↑](#footnote-ref-53)
54. Only available for a wider dataset which includes individuals over the age of 14, whose sleep data was not considered (n=106). [↑](#footnote-ref-54)
55. Only available for a wider dataset which includes individuals over the age of 14, whose sleep data was not considered (n=106). [↑](#footnote-ref-55)
56. Only available for a wider dataset which includes individuals who had other mucopolysaccharide disorders (n=76). See earlier reference to Gönüldaş et al., (2014). [↑](#footnote-ref-56)
57. Only available for a wider dataset which includes individuals who had other mucopolysaccharide disorders (n=76). See earlier reference to Gönüldaş et al., (2014). [↑](#footnote-ref-57)
58. Only 13 participants of the total sample (n=15) completed the measure of ‘general’ sleep difficulties. [↑](#footnote-ref-58)
59. Only available for a wider dataset which includes individuals over the age of 14, whose sleep data was not considered (n=35). [↑](#footnote-ref-59)
60. Only available for a wider dataset which includes individuals over the age of 14, whose sleep data was not considered (n=35). [↑](#footnote-ref-60)
61. Only 15 participants of the total sample (n=16) completed the measure of sleep-related breathing difficulties. [↑](#footnote-ref-61)
62. Only available for a wider dataset which includes individuals who had other mucopolysaccharide disorders (n=76). See earlier reference to Gönüldaş et al., (2014). [↑](#footnote-ref-62)
63. Only available for a wider dataset which includes individuals who had other mucopolysaccharide disorders (n=76). See earlier reference to Gönüldaş et al., (2014). [↑](#footnote-ref-63)
64. Only available for a wider dataset which includes age-matched comparison group of individuals who did not have NF1 (n=94369). [↑](#footnote-ref-64)
65. Obese group 28.1, Non-obese group 25.7 [↑](#footnote-ref-65)
66. Only 14 participants of the total sample (n=17) completed the measure of sleep-related breathing difficulties. [↑](#footnote-ref-66)
67. Only 11 participants of the total sample (n=20) completed the measure of sleep-related breathing difficulties. [↑](#footnote-ref-67)
68. Interquartile range. [↑](#footnote-ref-68)
69. Interquartile range. [↑](#footnote-ref-69)
70. Interquartile range. [↑](#footnote-ref-70)
71. Only 68 participants of the total sample (n=101) completed the measure of ‘general’ sleep difficulties. [↑](#footnote-ref-71)
72. Only 13 participants of the total sample (n=21) completed the measure of sleep-related breathing difficulties. [↑](#footnote-ref-72)
73. Gender was not reported for 95 participants [↑](#footnote-ref-73)
74. 12 participants aged 3-17 years (mean = 8.8), 17 participants aged 18-48 (mean = 32.6). [↑](#footnote-ref-74)
75. 31 participants aged <12 onths, 42 aged 13-24 months [↑](#footnote-ref-75)
76. Only 14 participants of the total sample (n=74) completed the measure of sleep-related breathing difficulties. [↑](#footnote-ref-76)
77. Only 35 participants of the total sample (n=101) completed the measure of sleep-related breathing difficulties. [↑](#footnote-ref-77)
78. Only 79 participants of the total sample (n=101) completed the measure of ‘general’ sleep difficulties. [↑](#footnote-ref-78)
79. Only 358 participants of the total sample (n=364) completed the measure of insomnia. [↑](#footnote-ref-79)
80. Only 360 participants of the total sample (n=364) completed the measure of sleep bruxism. [↑](#footnote-ref-80)
81. Age at first follow-up (2000). [↑](#footnote-ref-81)
82. Only 141 participants of the total sample (n=591) completed the measure of ‘general’ sleep difficulties. [↑](#footnote-ref-82)
83. Only 9 participants of the total sample (n=13) completed the measure of sleep bruxism. [↑](#footnote-ref-83)
84. Only 9 participants of the total sample (n=13) completed the measure of ‘general’ sleep difficulties. [↑](#footnote-ref-84)
85. 44 participants younger than 7 years, 51 aged 7-11 years, 53 aged 11-17 years, 50 aged ≥17 [↑](#footnote-ref-85)
86. Interquartile range [↑](#footnote-ref-86)
87. Only 200 participants of the total sample (n=217) completed the measure of ‘general’ sleep difficulties. [↑](#footnote-ref-87)
88. Only 74 participants of the total sample (n=237) completed the measure of insomnia. [↑](#footnote-ref-88)
89. Only 202 participants of the total sample (n=237) completed the measure of excessive daytime sleepiness. [↑](#footnote-ref-89)
90. Only 202 participants of the total sample (n=237) completed the measure of sleep bruxism. [↑](#footnote-ref-90)
91. Only 187 participants of the total sample (n=237) completed the measure of ‘general’ sleep difficulties. [↑](#footnote-ref-91)
92. Only 754 participants of the total sample (n=2216) completed the measure of ‘genera’ sleep difficulties. [↑](#footnote-ref-92)
93. Gender was unknown in8 participants. [↑](#footnote-ref-93)
94. Only 146 participants of the total sample (n=150) completed the measure of ‘general’ sleep difficulties. [↑](#footnote-ref-94)
95. 73 participants aged under 5, 108 participants aged 5-15, and 79 participants aged 15-25. [↑](#footnote-ref-95)
96. Only 14 participants of the total sample (n=28) completed the measure of ‘general’ sleep difficulties. [↑](#footnote-ref-96)
97. Only 219 participants of the total sample (n=231) completed the measure of sleep enuresis. [↑](#footnote-ref-97)
