# Supplementary material for: Sleep disorders in rare genetic syndromes: a meta-analysis of prevalence and profile
Source: Mol Autism. 2021 Feb 25;12:18. doi: 10.1186/s13229-021-00426-w (PMC7908701; doi:10.1186/s13229-021-00426-w)
Supplement: Supplementary file 6 — Additional file 6. Included references. [file 13229_2021_426_MOESM6_ESM.docx]

Additional File 6

1. Larson AM, Shinnick JE, Shaaya EA, Thiele EA, Thibert RL. Angelman syndrome in adulthood. Am J Med Genet A. 2015;167A:331–44.
2. Tan W-H, Bacino CA, Skinner SA, Anselm I, Barbieri-Welge R, Bauer-Carlin A, et al. Angelman syndrome: Mutations influence features in early childhood. Am J Med Genet A. 2011;155A:81–90.
3. Berry RJ, Leitner RP, Clarke AR, Einfeld SL. Behavioral aspects of Angelman syndrome: A case control study. Am J Med Genet. 2005;132A:8–12.
4. Summers JA, Allison DB, Lynch PS, Sandler L. Behaviour problems in Angelman syndrome. J Intellect Disabil Res. 1995;39 ( Pt 2):97–106.
5. Smith A, Wiles C, Haan E, McGill J, Wallace G, Dixon J, et al. Clinical features in 27 patients with Angelman syndrome resulting from DNA deletion. J Med Genet. 1996;33:107–12.
6. Radstaake M, Didden R, Peters-Scheffer N, Sigafoos J, Korzilius H, Curfs LMG. Educational Priorities for Individuals with Angelman Syndrome: A Study of Parents’ Perspectives. J Dev Phys Disabil. 2014;26:299–316.
7. Conant KD, Thibert RL, Thiele EA. Epilepsy and the sleep-wake patterns found in Angelman syndrome. Epilepsia. 2009;50:2497–500.
8. Wagner C, Niemczyk J, Equit M, Curfs L, von Gontard A. Incontinence in persons with Angelman syndrome. Eur J Pediatr. 2017;176:225–32.
9. Miodrag N, Peters S. Parent stress across molecular subtypes of children with Angelman syndrome. J Intellect Disabil Res. 2015;59:816–26.
10. Clarke DJ, Marston G. Problem behaviors associated with 15q- Angelman syndrome. Am J Ment Retard. 2000;105:25–31.
11. Bruni O, Ferri R, D’Agostino G, Miano S, Roccella M, Elia M. Sleep disturbances in Angelman syndrome: a questionnaire study. Brain Dev. 2004;26:233–40.
12. Goldman SE, Bichell TJ, Surdyka K, Malow BA. Sleep in children and adolescents with Angelman syndrome: association with parent sleep and stress. J Intellect Disabil Res. 2012;56:600–8.
13. Walz NC, Beebe D, Byars K. Sleep in individuals with Angelman syndrome: parent perceptions of patterns and problems. Am J Ment Retard. 2005;110:243–52.
14. Didden R, Korzilius H, Smits MG, Curfs LMG. Sleep problems in individuals with Angelman syndrome. Am J Ment Retard. 2004;109:275–84.
15. Stavinoha RC, Kline AD, Levy HP, Kimball A, Mettel TL, Ishman SL. Characterization of sleep disturbance in Cornelia de Lange Syndrome. Int J Pediatr Otorhinolaryngol. 2011;75:215–8.
16. Hall SS, Arron K, Sloneem J, Oliver C. Health and sleep problems in Cornelia de Lange Syndrome: a case control study. J Intellect Disabil Res. 2008;52:458–68.
17. Rajan R, Benke JR, Kline AD, Levy HP, Kimball A, Mettel TL, et al. Insomnia in Cornelia de Lange syndrome. Int J Pediatr Otorhinolaryngol. 2012;76:972–5.
18. Zambrelli E, Fossati C, Turner K, Taiana M, Vignoli A, Gervasini C, et al. Sleep disorders in Cornelia de Lange syndrome. Am J Med Genet C Semin Med Genet. 2016;172:214–21.
19. Deuce G, Howard S, Rose S, Fuggle C. A study of CHARGE Syndrome in the UK. British Journal of Visual Impairment. 2012;30:91–100.
20. Hartshorne N, Hudson A, MacCuspie J, Kennert B, Nacarato T, Hartshorne T, et al. Quality of life in adolescents and adults with CHARGE syndrome. Am J Med Genet A. 2016;170:2012–21.
21. Hartshorne TS, Heussler HS, Dailor AN, Williams GL, Papadopoulos D, Brandt KK. Sleep disturbances in CHARGE syndrome: types and relationships with behavior and caregiver well-being. Dev Med Child Neurol. 2009;51:143–50.
22. Roger G, Morisseau-Durand MP, Van Den Abbeele T, Nicollas R, Triglia JM, Narcy P, et al. The CHARGE association: the role of tracheotomy. Arch Otolaryngol Head Neck Surg. 1999;125:33–8.
23. Trider C-L, Corsten G, Morrison D, Hefner M, Davenport S, Blake K. Understanding obstructive sleep apnea in children with CHARGE syndrome. Int J Pediatr Otorhinolaryngol. 2012;76:947–53.
24. Maas APHM, Didden R, Korzilius H, Curfs LMG. Exploration of differences in types of sleep disturbance and severity of sleep problems between individuals with Cri du Chat syndrome, Down’s syndrome, and Jacobsen syndrome: a case control study. Res Dev Disabil. 2012;33:1773–9.
25. Maas APHM, Didden R, Korzilius H, Braam W, Smits MG, Curfs LMG. Sleep in individuals with Cri du Chat syndrome: a comparative study. J Intellect Disabil Res. 2009;53:704–15.
26. Sawatari H, Chishaki A, Nishizaka M, Matsuoka F, Yoshimura C, Kuroda H, et al. A Nationwide Cross-sectional Study on Congenital Heart Diseases and Symptoms of Sleep-disordered Breathing among Japanese Down’s Syndrome People. Intern Med. 2015;54:1003–8.
27. Skotko BG, Macklin EA, Muselli M, Voelz L, McDonough ME, Davidson E, et al. A predictive model for obstructive sleep apnea and Down syndrome. Am J Med Genet. 2017;173:889–96.
28. Cocchi R. A strange posture adopted by Down’s syndrome individuals when sleeping: An epidemiological survey on a cohort of 432 subjects. Italian Journal of Intellective Impairment. 1989;2:21–14.
29. Capone GT, Aidikoff JM, Taylor K, Rykiel N. Adolescents and young adults with down syndrome presenting to a medical clinic with depression: Co-morbid obstructive sleep apnea. Am J Med Genet. 2013;161:2188–96.
30. Wong CW. Adults With Intellectual Disabilities Living in Hong Kong’s Residential Care Facilities: A Descriptive Analysis of Health and Disease Patterns by Sex, Age, and Presence of Down Syndrome: Adults with Intellectual Disability in Hong Kong. Journal of Policy and Practice in Intellectual Disabilities. 2011;8:231–8.
31. Lin J-D, Lin L-P, Hsu S-W, Chen W-X, Lin F-G, Wu J-L, et al. Are early onset aging conditions correlated to daily activity functions in youth and adults with Down syndrome? Research in Developmental Disabilities. 2015;36:532–6.
32. Bhatia MS, Kabra M, Sapra S. Behavioral Problems in Children with Down Syndrome. INDIAN PEDIATRICS. 2005;42:6.
33. Turner S, Sloper P. Behaviour Problems Among Children with Down’s Syndrome: Prevalence, Persistence and Parental Appraisal. Journal of Applied Research in Intellectual Disabilities. 1996;9:129–44.
34. Prasher, V P, Filer, A. Behavioural disturbance in people with Down’s syndrome and dementia. Journal of Intellectual Disability Research. 1995;39:432–6.
35. Shires CB, Anold SL, Schoumacher RA, Dehoff GW, Donepudi SK, Stocks RM. Body mass index as an indicator of obstructive sleep apnea in pediatric Down syndrome. International Journal of Pediatric Otorhinolaryngology. 2010;74:768–72.
36. Cotton S, Richdale A. Brief report: Parental descriptions of sleep problems in children with autism, Down syndrome, and Prader–Willi syndrome. Research in Developmental Disabilities. 2006;27:151–61.
37. Areias CM, Sampaio-Maia B, Guimaraes H, Melo P, Andrade D. Caries in Portuguese children with Down syndrome. Clinics. 2011;66:1183–6.
38. Nisbet LC, Phillips NN, Hoban TF, O’Brien LM. Characterization of a sleep architectural phenotype in children with Down syndrome. Sleep Breath. 2015;19:1065–71.
39. Andreou G, Galanopoulou C, Gourgoulianis K, Karapetsas A, Molyvdas P. Cognitive status in Down syndrome individuals with sleep disordered breathing deficits (SDB). Brain and Cognition. 2002;50:145–9.
40. Convergent validity of actigraphy with polysomnography and parent-reports when measuring sleep in children with Down syndrome [Internet]. [cited 2020 Dec 8]. Available from: <https://www.ncbi.nlm.nih.gov/pmc/articles/PMC5847446/>
41. Ashworth A, Hill CM, Karmiloff-Smith A, Dimitriou D. Cross syndrome comparison of sleep problems in children with Down syndrome and Williams syndrome. Research in Developmental Disabilities. 2013;34:1572–80.
42. Stores R, Stores G. Evaluation of a Brief Group-Administered Instruction for Parents to Prevent or Minimize Sleep Problems in Young Children with Down Syndrome. Journal of Applied Research in Intellectual Disabilities. 2004;17:61–70.
43. Maas APHM, Didden R, Korzilius H, Curfs LMG. Exploration of differences in types of sleep disturbance and severity of sleep problems between individuals with Cri du Chat syndrome, Down’s syndrome, and Jacobsen syndrome: A case control study. Research in Developmental Disabilities. 2012;33:1773–9.
44. Jensen KM, Sevick CJ, Seewald LAS, Halbower AC, Davis MM, McCabe ERB, et al. Greater Risk of Hospitalization in Children With Down Syndrome and OSA at Higher Elevation. Chest. 2015;147:1344–51.
45. Hoffmire CA, Magyar CI, Connolly HV, Fernandez ID, van Wijngaarden E. High Prevalence of Sleep Disorders and Associated Comorbidities in a Community Sample of Children with Down Syndrome. JCSM [Internet]. 2014 [cited 2019 Jul 18]; Available from: <http://jcsm.aasm.org/ViewAbstract.aspx?pid=29434>
46. Niemczyk J, von Gontard A, Equit M, Medoff D, Wagner C, Curfs L. Incontinence in persons with Down Syndrome. Neurourology and Urodynamics. 2017;36:1550–6.
47. Urv TK, Zigman WB, Silverman W. Maladaptive Behaviors Related to Dementia Status in Adults With Down Syndrome. Am J Ment Retard. 2008;113:73.
48. Cooper S-A, Prasher VP. Maladaptive behaviours and symptoms of dementia in adults with Down’s syndrome compared with adults with intellectual disability of other aetiologies. Journal of Intellectual Disability Research. 2002;42:293–300.
49. Yam W, Tse P, Yu C, Chow C, But W, Li K, et al. Medical issues among children and teenagers with Down syndrome in Hong Kong. Downs Syndr Res Pract. 2008;12:138–40.
50. Alexander M, Petri H, Ding Y, Wandel C, Khwaja O, Foskett N. Morbidity and medication in a large population of individuals with Down syndrome compared to the general population. Dev Med Child Neurol. 2016;58:246–54.
51. Cocchi R. Mosaic forms in Down’s syndrome: A survey on sixteen cases. Italian Journal of Intellective Impairment. 1996;9:45–54.
52. Chen MA, Lander TR, Murphy C. Nasal health in Down syndrome: A cross-sectional study. Otolaryngol Head Neck Surg. 2006;134:741–5.
53. Telakivi T, Partinen M, Salmi T. NOCTURNAL PERIODIC BREATHING IN PATIENTS WITH MENTAL RETARDATION. Acta Neurologica Scandinavica. 2009;69:407–8.
54. Trois MS, Marcus CL, Ch MBB. Obstructive Sleep Apnea in Adults with Down Syndrome. Journal of Clinical Sleep Medicine. 2009;8.
55. Austeng ME, Øverland B, Kværner KJ, Andersson E-M, Axelsson S, Abdelnoor M, et al. Obstructive sleep apnea in younger school children with Down syndrome. International Journal of Pediatric Otorhinolaryngology. 2014;78:1026–9.
56. Breslin J, Spanò G, Bootzin R, Anand P, Nadel L, Edgin J. Obstructive sleep apnea syndrome and cognition in Down syndrome. Dev Med Child Neurol. 2014;56:657–64.
57. Ono J, Chishaki A, Ohkusa T, Sawatari H, Nishizaka M, Ando S. Obstructive sleep apnea-related symptoms in Japanese people with Down syndrome: Obstructive sleep apnea in down syndrome. Nurs Health Sci. 2015;17:420–5.
58. Shott SR, Amin R, Chini B, Heubi C, Hotze S, Akers R. Obstructive Sleep ApneaShould All Children With Down Syndrome Be Tested? ARCH OTOLARYNGOL HEAD NECK SURG. 2006;132:5.
59. Ng DK, Hui, H N, Chan, C H, Chow, P Y, Cheung, J M, Leung, S Y. Obstructive sleep apnoea in children with Down syndrome. Singapore Medical Journal. 2006;47:774–9.
60. Rosen D, Lombardo A, Skotko B, Davidson EJ. Parental Perceptions of Sleep Disturbances and Sleep-Disordered Breathing in Children With Down Syndrome. Clin Pediatr (Phila). 2011;50:121–5.
61. Breslin JH, Edgin JO, Bootzin RR, Goodwin JL, Nadel L. Parental report of sleep problems in Down syndrome: Parental report of sleep problems in Down syndrome. Journal of Intellectual Disability Research. 2011;55:1086–91.
62. Miamoto CB, Pereira LJ, Ramos-Jorge ML, Marques LS. Prevalence and predictive factors of sleep bruxism in children with and without cognitive impairment. Braz oral res. 2011;25:439–45.
63. Hill CM, Evans HJ, Elphick H, Farquhar M, Pickering RM, Kingshott R, et al. Prevalence and predictors of obstructive sleep apnoea in young children with Down syndrome. Sleep Medicine. 2016;27–28:99–106.
64. Maris M, Verhulst S, Wojciechowski M, Van de Heyning P, Boudewyns A. Prevalence of Obstructive Sleep Apnea in Children with Down Syndrome. Sleep. 2016;39:699–704.
65. de Miguel-Díez J, Villa-Asensi JR, Álvarez-Sala JL. Prevalence of Sleep-Disordered Breathing in Children with Down Syndrome: Polygraphic Findings in 108 Children. Sleep. 2003;26:1006–9.
66. Jensen KM, Taylor LC, Davis MM. Primary care for adults with Down syndrome: adherence to preventive healthcare recommendations: Primary care for adults with Down syndrome. J Intellect Disabil Res. 2013;57:409–21.
67. Dyken ME, Lin-Dyken DC, Poulton S, Zimmerman MB, Sedars E. Prospective Polysomnographic Analysis of Obstructive Sleep Apnea in Down Syndrome. Arch Pediatr Adolesc Med. 2003;157:655.
68. Patti PJ, Tsiouris JA. Psychopathology in adults with Down syndrome: Clinical findings from an outpatient clinic. International Journal on Disability and Human Development [Internet]. 2006 [cited 2019 Jul 18];5. Available from: <https://www.degruyter.com/view/j/ijdhd.2006.5.4/ijdhd.2006.5.4.357/ijdhd.2006.5.4.357.xml>
69. Konstantinopoulou S, Tapia IE, Kim JY, Xanthopoulos MS, Radcliffe J, Cohen MS, et al. Relationship between obstructive sleep apnea cardiac complications and sleepiness in children with Down syndrome. Sleep Medicine. 2016;17:18–24.
70. Churchill SS, Kieckhefer GM, Bjornson KF, Herting JR. Relationship between Sleep Disturbance and Functional Outcomes in Daily Life Habits of Children with Down Syndrome. Sleep. 2015;38:61–71.
71. Rahmawati A, Chishaki A, Ohkusa T, Sawatari H, Hashiguchi N, Ono J, et al. Relationship between sleep postures and sleep-disordered breathing parameters in people with Down syndrome in Japan: Sleep postures and SDB in Down syndrome. Sleep and Biological Rhythms. 2015;13:323–31.
72. Brooks LJ, Olsen MN, Bacevice AM, Beebe A, Konstantinopoulou S, Taylor HG. Relationship between sleep, sleep apnea, and neuropsychological function in children with Down syndrome. Sleep Breath. 2015;19:197–204.
73. Esbensen AJ, Hoffman EK. Reliability of parent report measures of sleep in children with Down syndrome: Reliability sleep measures in Down syndrome. Journal of Intellectual Disability Research. 2017;61:210–20.
74. Ferri R, Curzi‐Dascalova L, Del Gracco S, Elia M, Musumeci S, Stefanini M. Respiratory patterns during sleep in Down’s syndrome: importance of central apnoeas. Journal of Sleep Research. 1997;6:134–41.
75. Basil JS, Santoro SL, Martin LJ, Healy KW, Chini BA, Saal HM. Retrospective Study of Obesity in Children with Down Syndrome. The Journal of Pediatrics. 2016;173:143–8.
76. Sobey CG, Judkins CP, Sundararajan V, Phan TG, Drummond GR, Srikanth VK. Risk of Major Cardiovascular Events in People with Down Syndrome. Taniyama Y, editor. PLoS ONE. 2015;10:e0137093.
77. Kavanagh K, Kahane J, Kordan B. Risks and benefits of adenotonsillectomy for children with Down syndrome. American Journal of Mental Deficiency. 1986;91:22–9.
78. Capone GT, Goyal P, Grados M, Smith B, Kammann H. Risperidone Use in Children with Down Syndrome, Severe Intellectual Disability, and Comorbid Autistic Spectrum Disorders: A Naturalistic Study: Journal of Developmental & Behavioral Pediatrics. 2008;29:106–16.
79. Dahlqvist Å, Rask E, Rosenqvist C-J, Sahlin C, Franklin KA. Sleep Apnea and Down’s Syndrome. Acta Oto-Laryngologica. 2003;123:1094–7.
80. Edgin JO, Tooley U, Demara B, Nyhuis C, Anand P, Spanò G. Sleep Disturbance and Expressive Language Development in Preschool-Age Children With Down Syndrome. Child Dev. 2015;86:1984–98.
81. Maas APHM, Didden R, Korzilius H, Braam W, Smits MG, Curfs LMG. Sleep in individuals with Cri du Chat syndrome: a comparative study. Journal of Intellectual Disability Research. 2009;53:704–15.
82. Esbensen AJ. Sleep problems and associated comorbidities among adults with Down syndrome: Sleep problems in adults with Down syndrome. Journal of Intellectual Disability Research. 2016;60:68–79.
83. Maris M, Verhulst S, Wojciechowski M, Van de Heyning P, Boudewyns A. Sleep problems and obstructive sleep apnea in children with down syndrome, an overview. International Journal of Pediatric Otorhinolaryngology. 2016;82:12–5.
84. Carter M, McCaughey E, Annaz D, Hill CM. Sleep problems in a Down syndrome population. Archives of Disease in Childhood. 2009;94:308–10.
85. Bassell JL, Phan H, Leu R, Kronk R, Visootsak J. Sleep profiles in children with down syndrome. Am J Med Genet. 2015;167:1830–5.
86. Resta O, Foschino Barbaro M, Giliberti T, Caratozzolo G, Cagnazzo M, Scarpelli F, et al. Sleep related breathing disorders in adults with Down syndrome. Downs Syndr Res Pract. 2003;8:115–20.
87. Carskadon MA, Pueschel SM, Millman RP. Sleep-disordered breathing and behavior in three risk groups: preliminary findings from parental reports. Child’s Nerv Syst. 1993;9:452–7.
88. Chen C-C (Jj), Spanò G, Edgin JO. The impact of sleep disruption on executive function in Down syndrome. Research in Developmental Disabilities. 2013;34:2033–9.
89. Stores R, Stores G, Buckley S. The Pattern of Sleep Problems in Children with Down’s Syndrome and Other Intellectual Disabilities. Journal of Applied Research in Intellectual Disabilities. 1996;9:145–59.
90. Stores RJ, Stores G. The significance of aspects of screening for obstructive sleep apnoea in children with Down syndrome: Obstructive sleep apnoea in children with Down syndrome. J Intellect Disabil Res. 2014;58:381–92.
91. Virji-Babul N, Eichmann A, Kisly D, Down J, Haslam RH. Use of health care guidelines in patients with Down syndrome by family physicians across Canada. Paediatrics and Child Health [Internet]. 2007 [cited 2019 Jul 18]; Available from: <https://academic.oup.com/pch/article/12/3/179/2647890/Use-of-health-care-guidelines-in-patients-with>
92. Esbensen AJ, Beebe DW, Byars KC, Hoffman EK. Use of Sleep Evaluations and Treatments in Children with Down Syndrome: Journal of Developmental & Behavioral Pediatrics. 2016;37:629–36.
93. Richdale AL. A descriptive analysis of sleep behaviour in children with Fragile X. Journal of Intellectual & Developmental Disability. 2003;28:135–44.
94. Kronk R, Dahl R, Noll R. Caregiver Reports of Sleep Problems on a Convenience Sample of Children With Fragile X Syndrome. American Journal on Intellectual and Developmental Disabilities. 2009;114:383–92.
95. Kronk R, Bishop EE, Raspa M, Bickel JO, Mandel DA, Bailey DB. Prevalence, nature, and correlates of sleep problems among children with fragile X syndrome based on a large scale parent survey. Sleep. 2010;33:679–87.
96. Symons FJ, Byiers BJ, Raspa M, Bishop E, Bailey DB. Self-Injurious Behavior and Fragile X Syndrome: Findings From the National Fragile X Survey. American Journal on Intellectual and Developmental Disabilities. 2010;115:473–81.
97. Tirosh E, Borochowitz Z. Sleep apnea in fragile X syndrome. American Journal of Medical Genetics. 1992;43:124–7.
98. Tawfik TZ, Hashem S, Zaki MA, El-shazly N, Hegazy MM, El-Meguid NA, et al. Sleep Disorders in Fragile X Syndrome. 2009.
99. Bax MCO, Colville GA. Behaviour in mucopolysaccharide disorders. Archives of disease in childhood. 1995;73:77–81.
100. Muñoz-Rojas MV, Bay L, Sanchez L, van Kuijck M, Ospina S, Cabello JF, et al. Clinical manifestations and treatment of mucopolysaccharidosis type I patients in Latin America as compared with the rest of the world. J Inherit Metab Dis. 2011;34:1029–37.
101. Wraith JE, Clarke LA, Beck M, Kolodny EH, Pastores GM, Muenzer J, et al. Enzyme replacement therapy for mucopolysaccharidosis I: a randomized, double-blinded, placebo-controlled, multinational study of recombinant human α-L-iduronidase (laronidase). The Journal of Pediatrics. 2004;144:581–8.
102. Wraith JE, Beck M, Lane R, van der Ploeg A, Shapiro E, Xue Y, et al. Enzyme Replacement Therapy in Patients Who Have Mucopolysaccharidosis I and Are Younger Than 5 Years: Results of a Multinational Study of Recombinant Human -L-Iduronidase (Laronidase). PEDIATRICS. 2007;120:e37–46.
103. Soni-Jaiswal A, Mercer J, Jones SA, Bruce IA, Callery P. Mucopolysaccharidosis I; Parental beliefs about the impact of disease on the quality of life of their children. Orphanet J Rare Dis. 2016;11:96.
104. Ahmed A, Whitley CB, Cooksley R, Rudser K, Cagle S, Ali N, et al. Neurocognitive and neuropsychiatric phenotypes associated with the mutation L238Q of the α-L-iduronidase gene in Hurler–Scheie syndrome. Molecular Genetics and Metabolism. 2014;111:123–7.
105. Moreau J, Brassier A, Amaddeo A, Neven B, Caillaud C, Chabli A, et al. Obstructive sleep apnea syndrome after hematopoietic stem cell transplantation in children with mucopolysaccharidosis type I. Mol Genet Metab. 2015;116:275–80.
106. Pal AR, Langereis EJ, Saif MA, Mercer J, Church HJ, Tylee KL, et al. Sleep disordered breathing in mucopolysaccharidosis I: a multivariate analysis of patient, therapeutic and metabolic correlators modifying long term clinical outcome. Orphanet J Rare Dis. 2015;10:42.
107. Dualibi APFF, Martins AM, Moreira GA, de Azevedo MF, Fujita RR, Pignatari SSN. The impact of laronidase treatment in otolaryngological manifestations of patients with mucopolysaccharidosis. Brazilian Journal of Otorhinolaryngology. 2016;82:522–8.
108. Maas APHM, Didden R, Korzilius H, Curfs LMG. Exploration of differences in types of sleep disturbance and severity of sleep problems between individuals with Cri du Chat syndrome, Down’s syndrome, and Jacobsen syndrome: A case control study. Research in Developmental Disabilities. 2012;33:1773–9.
109. Maas APHM, Grossfeld PD, Didden R, Korzilius H, Braam WJ, Smits MG, et al. Sleep problems in individuals with 11q terminal deletion disorder (Jacobsen syndrome). Genet Couns. 2008;19:225–35.
110. Malcolm C, Hain R, Gibson F, Adams S, Anderson G, Forbat L. Challenging symptoms in children with rare life-limiting conditions: findings from a prospective diary and interview study with families: Challenging symptoms in rare LLCs. Acta Paediatrica. 2012;101:985–92.
111. Kirveskari E, Partinen M, Salmi T, Sainio K, Telakivi T, Hämäläinen M, et al. Sleep alterations in juvenile neuronal ceroid-lipofuscinosis. Pediatr Neurol. 2000;22:347–54.
112. Bax MCO, Colville GA. Behaviour in mucopolysaccharide disorders. Archives of disease in childhood. 1995;73:77–81.
113. Okuyama T, Tanaka A, Suzuki Y, Ida H, Tanaka T, Cox GF, et al. Japan Elaprase® Treatment (JET) study: Idursulfase enzyme replacement therapy in adult patients with attenuated Hunter syndrome (Mucopolysaccharidosis II, MPS II). Molecular Genetics and Metabolism. 2010;99:18–25.
114. Gönüldaş B, Yılmaz T, Sivri HS, Güçer KŞ, Kılınç K, Genç GA, et al. Mucopolysaccharidosis: Otolaryngologic findings, obstructive sleep apnea and accumulation of glucosaminoglycans in lymphatic tissue of the upper airway. Int J Pediatr Otorhinolaryngol. 2014;78:944–9.
115. Gönüldaş B, Yılmaz T, Sivri HS, Güçer KŞ, Kılınç K, Genç GA, et al. Mucopolysaccharidosis: Otolaryngologic findings, obstructive sleep apnea and accumulation of glucosaminoglycans in lymphatic tissue of the upper airway. Int J Pediatr Otorhinolaryngol. 2014;78:944–9.
116. Namazova-Baranova LS, Vashakmadze ND, Gevorkyan AK, Altunin VV, Kuzenkova LM, Chernavina EG, et al. OBSTRUCTIVE SLEEP APNEA SYNDROME IN CHILDREN WITH TYPE II MUCOPOLYSACCHARIDOSIS (HUNTER SYNDROME) [Internet]. Pediatric pharmacology. 2013 [cited 2019 Jul 18]. Available from: <https://pf.spr-journal.ru/jour/article/view/161>
117. Lin H-Y, Chen M-R, Lin C-C, Chen C-P, Lin D-S, Chuang C-K, et al. Polysomnographic characteristics in patients with mucopolysaccharidoses. Pediatr Pulmonol. 2010;45:1205–12.
118. Wooten WI, Muenzer J, Vaughn BV, Muhlebach MS. Relationship of Sleep to Pulmonary Function in Mucopolysaccharidosis II. The Journal of Pediatrics. 2013;162:1210–5.
119. Mahon LV, Lomax M, Grant S, Cross E, Hare DJ, Wraith JE, et al. Assessment of Sleep in Children with Mucopolysaccharidosis Type III. Ginsberg SD, editor. PLoS ONE. 2014;9:e84128.
120. Bax MCO, Colville GA. Behaviour in mucopolysaccharide disorders. Archives of disease in childhood. 1995;73:77–81.
121. Malcolm C, Hain R, Gibson F, Adams S, Anderson G, Forbat L. Challenging symptoms in children with rare life-limiting conditions: findings from a prospective diary and interview study with families: Challenging symptoms in rare LLCs. Acta Paediatrica. 2012;101:985–92.
122. Ruijter GJG, Valstar MJ, van de Kamp JM, van der Helm RM, Durand S, van Diggelen OP, et al. Clinical and genetic spectrum of Sanfilippo type C (MPS IIIC) disease in The Netherlands. Molecular Genetics and Metabolism. 2008;93:104–11.
123. Gönüldaş B, Yılmaz T, Sivri HS, Güçer KŞ, Kılınç K, Genç GA, et al. Mucopolysaccharidosis: Otolaryngologic findings, obstructive sleep apnea and accumulation of glucosaminoglycans in lymphatic tissue of the upper airway. Int J Pediatr Otorhinolaryngol. 2014;78:944–9.
124. Fraser J. Sleep disturbance in Sanfilippo syndrome: a parental questionnaire study. Archives of Disease in Childhood. 2005;90:1239–42.
125. Colville GA, Walters JP, Yule W, Bax M. SLEEP PROBLEMS IN CHILDREN WITH SANFILIPPO SYNDROME. Developmental Medicine & Child Neurology. 2008;38:538–44.
126. Bax MCO, Colville GA. Behaviour in mucopolysaccharide disorders. Archives of disease in childhood. 1995;73:77–81.
127. Gönüldaş B, Yılmaz T, Sivri HS, Güçer KŞ, Kılınç K, Genç GA, et al. Mucopolysaccharidosis: Otolaryngologic findings, obstructive sleep apnea and accumulation of glucosaminoglycans in lymphatic tissue of the upper airway. Int J Pediatr Otorhinolaryngol. 2014;78:944–9.
128. Madubata CC, Olsen MA, Stwalley DL, Gutmann DH, Johnson KJ. Neurofibromatosis type 1 and chronic neurological conditions in the United States: an administrative claims analysis. Genet Med. 2015;17:36–42.
129. Licis AK, Vallorani A, Gao F, Chen C, Lenox J, Yamada KA, et al. Prevalence of Sleep Disturbances in Children With Neurofibromatosis Type 1. J Child Neurol. 2013;28:1400–5.
130. Maraña Pérez AI, Duat Rodríguez A, Soto Insuga V, Domínguez Carral J, Puertas Martín V, González Gutiérrez Solana L. Prevalencia de trastornos del sueño en pacientes con neurofibromatosis tipo 1. Neurología. 2015;30:561–5.
131. Johnson H, Wiggs L, Stores G, Huson SM. Psychological disturbance and sleep disorders in children with neurofibromatosis type 1. Dev Med Child Neurol. 2005;47:237–42.
132. Leschziner GD, Golding JF, Ferner RE. Sleep disturbance as part of the neurofibromatosis type 1 phenotype in adults. Am J Med Genet. 2013;161:1319–22.
133. Partsch CA, Lämmer C, Gillessen-Kaesbach G, Pankau R. Adult patients with Prader-Willi syndrome: Clinical characteristics, life circumstances and growth hormone secretion. Growth Hormone & IGF Research. 2000;10:S81–5.
134. Clarke DJ, Waters J, Corbett JA. Adults with Prader-Willi Syndrome: Abnormalities of Sleep and Behaviour. J R Soc Med. 1989;82:21–4.
135. Verrillo E, Bruni O, Franco P, Ferri R, Thiriez G, Pavone M, et al. Analysis of NREM sleep in children with Prader–Willi syndrome and the effect of growth hormone treatment. Sleep Medicine. 2009;10:646–50.
136. Vandeleur M, Davey MJ, Nixon GM. Are sleep studies helpful in children with Prader-Willi syndrome prior to commencement of growth hormone therapy?: Sleep studies in Prader-Willi syndrome. J Paediatr Child Health. 2013;49:238–41.
137. Arens R, Gozal D, Burrell BC, Bailey SL, Bautista DB, Keens TG, et al. Arousal and cardiorespiratory responses to hypoxia in Prader-Willi syndrome. Am J Respir Crit Care Med. 1996;153:283–7.
138. Cotton S, Richdale A. Brief report: Parental descriptions of sleep problems in children with autism, Down syndrome, and Prader–Willi syndrome. Research in Developmental Disabilities. 2006;27:151–61.
139. Torrado M, Araoz V, Baialardo E, Abraldes K, Mazza C, Krochik G, et al. Clinical-etiologic correlation in children with Prader-Willi syndrome (PWS): An interdisciplinary study. Am J Med Genet. 2007;143A:460–8.
140. Cohen M, Hamilton J, Narang I. Clinically Important Age-Related Differences in Sleep Related Disordered Breathing in Infants and Children with Prader-Willi Syndrome. Baumert M, editor. PLoS ONE. 2014;9:e101012.
141. Festen DAM, Wevers M, de Weerd AW, van den Bossche RAS, Duivenvoorden HJ, Hokken-Koelega ACS. Cognition and behavior in pre-pubertal children with Prader-Willi syndrome and associations with sleep-related breathing disorders. Am J Med Genet. 2008;146A:3018–25.
142. Vgontzas AN, Bixler EO, Kales A, Centurione A, Rogan PK, Mascari M, et al. Daytime Sleepines and Rem Abrormalities in Prader-Willi Syndrome: Evidence of Generalized Hypoarousal. International Journal of Neuroscience. 1996;87:127–39.
143. Boer H, Clarke D. Development and Behaviour in Genetic Syndromes: Prader-Willi Syndrome. Journal of Applied Research in Intellectual Disabilities. 1999;12:294–301.
144. Lan M-C, Hsu Y-B, Lan M-Y, Chiu T-J, Huang T-T, Wong S-B, et al. Drug-induced sleep endoscopy in children with Prader-Willi syndrome. Sleep Breath. 2016;20:1029–34.
145. Beauloye V, Dhondt K, Buysse W, Nyakasane A, Zech F, De Schepper J, et al. Evaluation of the hypothalamic-pituitary-adrenal axis and its relationship with central respiratory dysfunction in children with Prader-Willi syndrome. Orphanet J Rare Dis. 2015;10:106.
146. Berini J, Spica Russotto V, Castelnuovo P, Di Candia S, Gargantini L, Grugni G, et al. Growth Hormone Therapy and Respiratory Disorders: Long-Term Follow-up in PWS Children. The Journal of Clinical Endocrinology & Metabolism. 2013;98:E1516–23.
147. Schrander-Stumpel CTRM, Sinnema M, van den Hout L, Maaskant MA, van Schrojenstein Lantman-de Valk HMJ, Wagemans A, et al. Healthcare transition in persons with intellectual disabilities: General issues, the Maastricht model, and Prader–Willi syndrome. Am J Med Genet. 2007;145C:241–7.
148. Manni R, Politini L, Nobili L, Ferrillo F, Livieri C, Veneselli E, et al. Hypersomnia in the Prader Willi syndrome: clinical-electrophysiological features and underlying factors. Clinical Neurophysiology. 2001;112:800–5.
149. Arens R, Gozal D, Omlin KJ, Livingston FR, Liu J, Keens TG, et al. Hypoxic and hypercapnic ventilatory responses in Prader-Willi syndrome. Journal of Applied Physiology. 1994;77:2224–30.
150. Harris JC. Is Excessive Daytime Sleepiness Characteristic of Prader-Willi Syndrome?: The Effects of Weight Change. Arch Pediatr Adolesc Med. 1996;150:1288.
151. Khayat A, Narang I, Bin-Hasan S, Amin R, Al-Saleh S. Longitudinal evaluation of sleep disordered breathing in infants with Prader-Willi syndrome. Arch Dis Child. 2017;102:634–8.
152. Al-Saleh S, Al-Naimi A, Hamilton J, Zweerink A, Iaboni A, Narang I. Longitudinal Evaluation of Sleep-Disordered Breathing in Children with Prader-Willi Syndrome during 2 Years of Growth Hormone Therapy. The Journal of Pediatrics. 2013;162:263-268.e1.
153. Laurier V, Lapeyrade A, Copet P, Demeer G, Silvie M, Bieth E, et al. Medical, psychological and social features in a large cohort of adults with Prader-Willi syndrome: experience from a dedicated centre in France: Medical, psychological and social features of adults with PWS. J Intellect Disabil Res. 2015;59:411–21.
154. Meyer SL, Splaingard M, Repaske DR, Zipf W, Atkins J, Jatana K. Outcomes of Adenotonsillectomy in Patients With Prader-Willi Syndrome. Arch Otolaryngol Head Neck Surg. 2012;138:1047.
155. Lin H-Y, Lin S-P, Lin C-C, Tsai L-P, Chen M-R, Chuang C-K, et al. Polysomnographic characteristics in patients with Prader–Willi syndrome. Pediatr Pulmonol. 2007;42:881–7.
156. Viardot A, Sze L, Purtell L, Sainsbury A, Loughnan G, Smith E, et al. Prader-Willi Syndrome Is Associated with Activation of the Innate Immune System Independently of Central Adiposity and Insulin Resistance. The Journal of Clinical Endocrinology & Metabolism. 2010;95:3392–9.
157. Butler JV, Whittington JE, Holland AJ, Boer H, Clarke D, Webb T. Prevalence of, and risk factors for, physical ill-health in people with Prader-Willi syndrome: a population-based study. Dev Med Child Neurol. 2002;44:248.
158. Festen DAM, Wevers M, de Weerd AW, van den Bossche RAS, Duivenvoorden HJ, Otten BJ, et al. Psychomotor Development in Infants with Prader-Willi Syndrome and Associations with Sleep-Related Breathing Disorders. Pediatr Res. 2007;62:221–4.
159. Schlüter B, Buschatz D, Trowitzsch E, Aksu F, Andler W. Respiratory control in children with Prader-Willi syndrome. Eur J Pediatr. 1996;156:65–8.
160. Fillion M, Deal C, Van Vliet G. Retrospective Study of the Potential Benefits and Adverse Events during Growth Hormone Treatment in Children with Prader-Willi Syndrome. The Journal of Pediatrics. 2009;154:230-233.e1.
161. Maas APHM, Didden R, Bouts L, Smits MG, Curfs LMG. Scatter plot analysis of excessive daytime sleepiness and severe disruptive behavior in adults with Prader-Willi syndrome: A pilot study. Research in Developmental Disabilities. 2009;30:529–37.
162. Miller J, Silverstein J, Shuster J, Driscoll DJ, Wagner M. Short-Term Effects of Growth Hormone on Sleep Abnormalities in Prader-Willi Syndrome. The Journal of Clinical Endocrinology & Metabolism. 2006;91:413–7.
163. Richdale AL, Cotton S, Hibbit K. Sleep and behaviour disturbance in Prader-Willi syndrome: a questionnaire study: Journal of Intellectual Disability Research VOLUME 43 PART 5 OCTOBER 1999. Journal of Intellectual Disability Research. 1999;43:380–92.
164. Clift S, Dahlitz M, Parkes JD. Sleep Apnea in the Prader-Willi syndrome. Journal of Sleep Research. 1994;3:121–6.
165. Priano L, Grugni G, Miscio G, Guastamacchia G, Toffolet L, Sartorio A, et al. Sleep cycling alternating pattern (CAP) expression is associated with hypersomnia and GH secretory pattern in Prader–Willi syndrome. Sleep Medicine. 2006;7:627–33.
166. Miller JL. Sleep Disordered Breathing in Infants with Prader-Willi Syndrome During the First 6 Weeks of Growth Hormone Therapy: A Pilot Study. Journal of Clinical Sleep Medicine. 2009;7.
167. Pavone M, Caldarelli V, Khirani S, Colella M, Ramirez A, Aubertin G, et al. Sleep disordered breathing in patients with Prader-Willi syndrome: A multicenter study: Sleep in Prader-Willi Syndrome. Pediatr Pulmonol. 2015;50:1354–9.
168. Maas APHM, Sinnema M, Didden R, Maaskant MA, Smits MG, Schrander-Stumpel CTRM, et al. Sleep disturbances and behavioural problems in adults with Prader-Willi syndrome: Sleep and behaviour in adults with Prader-Willi syndrome. Journal of Intellectual Disability Research. 2010;54:906–17.
169. O’Donoghue FJ, Camfferman D, Kennedy JD, Martin AJ, Couper T, Lack LD, et al. Sleep-Disordered Breathing in Prader-Willi Syndrome and its Association with Neurobehavioral Abnormalities. The Journal of Pediatrics. 2005;147:823–9.
170. Festen DAM, de Weerd AW, van den Bossche RAS, Joosten K, Hoeve H, Hokken-Koelega ACS. Sleep-Related Breathing Disorders in Prepubertal Children with Prader-Willi Syndrome and Effects of Growth Hormone Treatment. The Journal of Clinical Endocrinology & Metabolism. 2006;91:4911–5.
171. Gunay-Aygun M, Schwartz S, Heeger S, O’Riordan MA, Cassidy SB. The Changing Purpose of Prader-Willi Syndrome Clinical Diagnostic Criteria and Proposed Revised Criteria. PEDIATRICS. 2001;108:e92–e92.
172. Helbing-Zwanenburg B, Kamphuisen HAC, Mourtazaev MS. The origin of excessive daytime sleepiness in the Prader-Willi syndrome. Journal of Intellectual Disability Research. 1993;37:533–41.
173. van Wijngaarden RFA de L, Joosten KFM, van den Berg S, Otten BJ, de Jong FH, Sweep CGJ (Fred), et al. The Relationship between Central Adrenal Insufficiency and Sleep-Related Breathing Disorders in Children with Prader-Willi Syndrome. The Journal of Clinical Endocrinology & Metabolism. 2009;94:2387–93.
174. Richards A, Quaghebeur G, Clift S, Holland A, Dahlitz M, Parkes D. The upper airway and sleep apnoea in the Prader-Willi syndrome. Clin Otolaryngol. 1994;19:193–7.
175. Piazza CC, Fisher W, Kiesewetter K, Bowman L, Moser H. Aberrant sleep patterns in children with the rett syndrome. Brain and Development. 1990;12:488–93.
176. Halbach NSJ, Smeets EEJ, Schrander-Stumpel CTRM, van Schrojenstein Lantman de Valk HHJ, Maaskant MA, Curfs LMG. Aging in people with specific genetic syndromes: Rett syndrome. Am J Med Genet. 2008;146A:1925–32.
177. Zappella M, Genazzani A, Facchinetti F, Hayek G. Bromocriptine in the rett syndrome. Brain and Development. 1990;12:221–5.
178. Boban S, Wong K, Epstein A, Anderson B, Murphy N, Downs J, et al. Determinants of sleep disturbances in Rett syndrome: Novel findings in relation to genotype. Am J Med Genet. 2016;170:2292–300.
179. Marcus CL, Carroll JL, McColley SA, Loughlin GM, Curtis S, Pyzik P, et al. Polysomnographic characteristics of patients with Rett syndrome. The Journal of Pediatrics. 1994;125:218–24.
180. Mangatt M, Wong K, Anderson B, Epstein A, Hodgetts S, Leonard H, et al. Prevalence and onset of comorbidities in the CDKL5 disorder differ from Rett syndrome. Orphanet J Rare Dis. 2016;11:39.
181. Hara M, Nishi Y, Yamashita Y, Hirata R, Takahashi S, Nagamitsu S, et al. Relation between circulating levels of GH, IGF-1, ghrelin and somatic growth in Rett syndrome. Brain and Development. 2014;36:794–800.
182. Hagebeuk EEO, Bijlmer RPGM, Koelman JHTM, Poll-The BT. Respiratory Disturbances in Rett Syndrome: Don’t Forget to Evaluate Upper Airway Obstruction. J Child Neurol. 2012;27:888–92.
183. Ho, H. H., Wong, P. K., Robertson, R. Rett syndrome: clinical profile and EEG abnormalities. Clinical & Investigative Medicine. 1988;11:234–41.
184. Percy AK, Zoghbi HY, Glaze DG. Rett syndrome: Discrimination of typical and variant forms. Brain and Development. 1987;9:458–61.
185. Glaze DG, Frost JD, Zoghbi HY, Percy AK. Rett’s syndrome: Characterization of respiratory patterns and sleep. Ann Neurol. 1987;21:377–82.
186. Young D, Nagarajan L, de Klerk N, Jacoby P, Ellaway C, Leonard H. Sleep problems in Rett syndrome. Brain and Development. 2007;29:609–16.
187. Fehr S, Wilson M, Downs J, Williams S, Murgia A, Sartori S, et al. The CDKL5 disorder is an independent clinical entity associated with early-onset encephalopathy. Eur J Hum Genet. 2013;21:266–73.
188. Wong K, Leonard H, Jacoby P, Ellaway C, Downs J. The trajectories of sleep disturbances in Rett syndrome. J Sleep Res. 2015;24:223–33.
189. Anderson A, Wong K, Jacoby P, Downs J, Leonard H. Twenty years of surveillance in Rett syndrome: what does this tell us? Orphanet J Rare Dis. 2014;9:87.
190. Freeman KA, Olufs E, Tudor M, Roullet J-B, Steiner RD. A Pilot Study of the Association of Markers of Cholesterol Synthesis with Disturbed Sleep in Smith-Lemli-Opitz Syndrome: Journal of Developmental & Behavioral Pediatrics. 2016;37:424–30.
191. Zarowski M, Vendrame M, Irons M, Kothare SV. Prevalence of sleep problems in Smith-Lemli-Opitz syndrome. Am J Med Genet. 2011;155:1558–62.
192. Boddaert N, De Leersnyder H, Bourgeois M, Munnich A, Brunelle F, Zilbovicius M. Anatomical and functional brain imaging evidence of lenticulo-insular anomalies in Smith Magenis syndrome. NeuroImage. 2004;21:1021–5.
193. Potocki L. Circadian rhythm abnormalities of melatonin in Smith-Magenis syndrome. Journal of Medical Genetics. 2000;37:428–33.
194. Loviglio MN, Beck CR, White JJ, Leleu M, Harel T, Guex N, et al. Identification of a RAI1-associated disease network through integration of exome sequencing, transcriptomics, and 3D genomics. Genome Med. 2016;8:105.
195. De Leersnyder H, de Blois M-C, Claustrat B, Romana S, Albrecht U, von Kleist-Retzow J-C, et al. Inversion of the circadian rhythm of melatonin in the Smith-Magenis syndrome. The Journal of Pediatrics. 2001;139:111–6.
196. Greenberg F, Guzzetta V, Montes de Oca-Luna R, Magenis RE, Smith AC, Richter SF, et al. Molecular analysis of the Smith-Magenis syndrome: a possible contiguous-gene syndrome associated with del(17)(p11.2). Am J Hum Genet. 1991;49:1207–18.
197. Smith ACM, Dykens E, Greenberg F. Sleep disturbance in smith-magenis syndrome (del 17 p11.2). American Journal of Medical Genetics. 1998;81:186–91.
198. Vignoli A, La Briola F, Peron A, Turner K, Vannicola C, Saccani M, et al. Autism spectrum disorder in tuberous sclerosis complex: searching for risk markers. Orphanet Journal of Rare Diseases. 2015;10:154.
199. van Eeghen AM, Numis AI, Staley BA, Therrien SE, Thibert RL, Thiele EA. Characterizing sleep disorders of adults with tuberous sclerosis complex: A questionnaire-based study and review. Epilepsy & Behavior. 2011;20:68–74.
200. Hunt A. Development, behaviour and seizures in 300 cases of tuberous sclerosis. Journal of Intellectual Disability Research. 1993;37:41–51.
201. Hunt A, Stores G. SLEEP DISORDER AND EPILEPSY IN CHILDREN WITH TUBEROUS SCLEROSIS: A QUESTIONNAIRE-BASED STUDY. Developmental Medicine & Child Neurology. 1994;36:108–15.
202. Bruni O, Cortesi F, Giannotti F, Curatolo P. Sleep disorders in tuberous sclerosis: a polysomnographic study. Brain Dev. 1995;17:52–6.
203. Sniecinska-Cooper AM, Iles RK, Butler SA, Jones H, Bayford R, Dimitriou D. Abnormal secretion of melatonin and cortisol in relation to sleep disturbances in children with Williams syndrome. Sleep Medicine. 2015;16:94–100.
204. Kirchner RM, Martens MA, Andridge RR. Adaptive Behavior and Development of Infants and Toddlers with Williams Syndrome. Front Psychol. 2016;7:598.
205. Einfeld SL, Tonge BJ, Florio T. Behavioral and emotional disturbance in individuals with Williams syndrome. Am J Ment Retard. 1997;102:45–53.
206. Annaz D, Hill CM, Ashworth A, Holley S, Karmiloff-Smith A. Characterisation of sleep problems in children with Williams syndrome. Research in Developmental Disabilities. 2011;32:164–9.
207. Santoro SD, Giacheti CM, Rossi NF, Campos LMG, Pinato L. Correlations between behavior, memory, sleep-wake and melatonin in Williams-Beuren syndrome. Physiology & Behavior. 2016;159:14–9.
208. Ashworth A, Hill CM, Karmiloff-Smith A, Dimitriou D. Cross syndrome comparison of sleep problems in children with Down syndrome and Williams syndrome. Research in Developmental Disabilities. 2013;34:1572–80.
209. von Gontard A, Niemczyk J, Borggrefe-Moussavian S, Wagner C, Curfs L, Equit M. Incontinence in children, adolescents and adults with Williams syndrome: Incontinence and Williams Syndrome. Neurourol Urodynam. 2016;35:1000–5.
210. Sammour ZM, de Bessa Jr J, Hisano M, Bruschini H, Kim CA, Srougi M, et al. Lower urinary tract symptoms in children and adolescents with Williams-Beuren syndrome. Journal of Pediatric Urology. 2016;S1477513116303242.
211. Arens R, Wright B, Elliott J, Zhao H, Wang PP, Brown LW, et al. Periodic limb movement in sleep in children with Williams syndrome. The Journal of Pediatrics. 1998;133:670–4.
212. Mason TBA, Arens R, Sharman J, Bintliff-Janisak B, Schultz B, Walters AS, et al. Sleep in children with Williams Syndrome. Sleep Medicine. 2011;12:892–7.
213. Goldman SE, Malow BA, Newman KD, Roof E, Dykens EM. Sleep patterns and daytime sleepiness in adolescents and young adults with Williams syndrome. Journal of Intellectual Disability Research. 2009;53:182–8.
214. Axelsson EL, Hill CM, Sadeh A, Dimitriou D. Sleep problems and language development in toddlers with Williams syndrome. Research in Developmental Disabilities. 2013;34:3988–96.
215. Trickett J, Heald M, Oliver C, Richards C. A cross-syndrome cohort comparison of sleep disturbance in children with Smith-Magenis syndrome, Angelman syndrome, autism spectrum disorder and tuberous sclerosis complex. J Neurodev Disord. 2018;10:9.
216. Kuroda H, Sawatari H, Ando S, Ohkusa T, Rahmawati A, Ono J, et al. A nationwide, cross-sectional survey on unusual sleep postures and sleep-disordered breathing-related symptoms in people with Down syndrome. J Intellect Disabil Res. 2017;61:656–67.
217. Facchina G, Amaddeo A, Baujat G, Breton S, Michot C, Thierry B, et al. A retrospective study on sleep-disordered breathing in Morquio-A syndrome. Am J Med Genet A. 2018;176:2595–603.
218. Friedman NR, Ruiz AG, Gao D, Ingram DG. Accuracy of Parental Perception of Nighttime Breathing in Children with Down Syndrome. Otolaryngol Head Neck Surg. 2018;158:364–7.
219. Knollman PD, Heubi CH, Meinzen-Derr J, Smith DF, Shott SR, Wiley S, et al. Adherence to Guidelines for Screening Polysomnography in Children with Down Syndrome. Otolaryngol Head Neck Surg. 2019;161:157–63.
220. Prasad A, Grocott O, Parkin K, Larson A, Thibert RL. Angelman syndrome in adolescence and adulthood: A retrospective chart review of 53 cases. Am J Med Genet A. 2018;176:1327–34.
221. Ikizoglu NB, Kiyan E, Polat B, Ay P, Karadag B, Ersu R. Are home sleep studies useful in diagnosing obstructive sleep apnea in children with Down syndrome? European Respiratory Journal [Internet]. European Respiratory Society; 2017 [cited 2020 Dec 8];50. Available from: <https://erj.ersjournals.com/content/50/suppl_61/PA1298>
222. Suzuki Y, Taylor M, Orii K, Fukao T, Orii T, Tomatsu S. Assessment of Activity of Daily Life in Mucopolysaccharidosis Type II Patients with Hematopoietic Stem Cell Transplantation. Diagnostics (Basel). 2020;10.
223. Jiménez-Arredondo RE, Brambila-Tapia AJL, Mercado-Silva FM, Ortiz-Aranda M, Benites-Godinez V, Olmos-García-de-Alba G, et al. Association between brain structural anomalies, electroencephalogram and history of seizures in Mucopolysaccharidosis type II (Hunter syndrome). Neurol Sci. 2017;38:445–50.
224. Kaufmann WE, Kidd SA, Andrews HF, Budimirovic DB, Esler A, Haas-Givler B, et al. Autism Spectrum Disorder in Fragile X Syndrome: Cooccurring Conditions and Current Treatment. Pediatrics. American Academy of Pediatrics; 2017;139:S194–206.
225. Hayes SA, Kutty S, Thomas J, Johnson JT, Yetman AT. Cardiovascular and general health status of adults with Trisomy 21. Int J Cardiol. 2017;241:173–6.
226. Sanjeeva GN, Maganthi M, Kodishala H, Marol RKR, Kulshreshtha PS, Lorenzetto E, et al. Clinical and Molecular Characterization of Prader-Willi Syndrome. Indian J Pediatr. 2017;84:815–21.
227. Sanjeeva GN, Maganthi M, Kodishala H, Marol RKR, Kulshreshtha PS, Lorenzetto E, et al. Clinical and Molecular Characterization of Prader-Willi Syndrome. Indian J Pediatr. 2017;84:815–21.
228. Ho NT, Kroner B, Grinspan Z, Fureman B, Farrell K, Zhang J, et al. Comorbidities of Rare Epilepsies: Results from the Rare Epilepsy Network. J Pediatr. 2018;203:249-258.e5.
229. Proffitt J, Osann K, McManus B, Kimonis VE, Heinemann J, Butler MG, et al. Contributing Factors of Mortality in Prader-Willi Syndrome. Am J Med Genet A. 2019;179:196–205.
230. Esbensen AJ, Hoffman EK, Stansberry E, Shaffer R. Convergent validity of actigraphy with polysomnography and parent-reports when measuring sleep in children with Down syndrome. J Intellect Disabil Res. 2018;62:281–91.
231. Durhan MA, Agrali OB, Kiyan E, Ikizoglu NB, Ersu R, Tanboga I. Does obstructive sleep apnea affect oral and periodontal health in children with down syndrome? A preliminary study. Niger J Clin Pract. 2019;22:1175–9.
232. Marschik PB, Lemcke S, Einspieler C, Zhang D, Bölte S, Townend GS, et al. Early development in Rett syndrome - the benefits and difficulties of a birth cohort approach. Dev Neurorehabil. 2018;21:68–72.
233. Sueri C, Ferlazzo E, Elia M, Bonanni P, Randazzo G, Gasparini S, et al. Epilepsy and sleep disorders improve in adolescents and adults with Angelman syndrome: A multicenter study on 46 patients. Epilepsy Behav. 2017;75:225–9.
234. Poskanzer SA, Hobensack VL, Ciciora SL, Santoro SL. Feeding difficulty and gastrostomy tube placement in infants with Down syndrome. Eur J Pediatr. 2020;179:909–17.
235. Saeves R, Strøm F, Sandvik L, Nordgarden H. Gastro-oesophageal reflux - an important causative factor of severe tooth wear in Prader-Willi syndrome? Orphanet J Rare Dis. 2018;13:64.
236. Mengoni SE, Redman S. Health monitoring of young children with Down syndrome: A parent-report study. British Journal of Learning Disabilities. 2020;48:10–8.
237. Fjermestad KW, Nyhus L, Kanavin ØJ, Heiberg A, Hoxmark LB. Health Survey of Adults with Neurofibromatosis 1 Compared to Population Study Controls. J Genet Counsel. 2018;27:1102–10.
238. Khan N, Cabo R, Tan W, Tayag R, Bird LM. Healthcare burden among individuals with Angelman syndrome: Findings from the Angelman Syndrome Natural History Study. Mol Genet Genomic Med [Internet]. 2019 [cited 2020 Dec 8];7. Available from: <https://www.ncbi.nlm.nih.gov/pmc/articles/PMC6625091/>
239. Posada AM, Isaza N, Panqueva P, Rondon-Sepulveda MA, Hidalgo P. High Incidence of Sleep-Related Breathing Disorders in Children with Down Syndrome Referred to a High-Altitude Sleep Laboratory. High Altitude Medicine & Biology. 2019;20:231–5.
240. Hill CM, Elphick HE, Farquhar M, Gringras P, Pickering RM, Kingshott RN, et al. Home oximetry to screen for obstructive sleep apnoea in Down syndrome. Arch Dis Child. 2018;103:962–7.
241. Abel F, Tan H-L, Negro V, Bridges N, Carlisle T, Chan E, et al. Hypoventilation disproportionate to OSAS severity in children with Prader-Willi syndrome. Archives of Disease in Childhood. BMJ Publishing Group Ltd; 2019;104:166–71.
242. Long-term health outcomes in patients with Prader–Willi Syndrome: a nationwide cohort study in Denmark | International Journal of Obesity [Internet]. [cited 2020 Dec 8]. Available from: <https://www.nature.com/articles/ijo2017139>
243. Mori Y, Downs J, Wong K, Leonard H. Longitudinal effects of caregiving on parental well-being: the example of Rett syndrome, a severe neurological disorder. Eur Child Adolesc Psychiatry. 2019;28:505–20.
244. Scheermeyer E, Harris M, Hughes I, Crock PA, Ambler G, Verge CF, et al. Low dose growth hormone treatment in infants and toddlers with Prader-Willi syndrome is comparable to higher dosage regimens. Growth Horm IGF Res. 2017;34:1–7.
245. Barboni MTS, Bueno C, Nagy BV, Maia PL, Vidal KSM, Alves RC, et al. Melanopsin System Dysfunction in Smith-Magenis Syndrome Patients. Invest Ophthalmol Vis Sci. 2018;59:362–9.
246. Lin H-Y, Chuang C-K, Lee C-L, Tu R-Y, Lo Y-T, Chiu PC, et al. Mucopolysaccharidosis III in Taiwan: Natural history, clinical and molecular characteristics of 28 patients diagnosed during a 21-year period. Am J Med Genet A. 2018;176:1799–809.
247. Koehne T, Müller-Stöver S, Köhn A, Stumpfe K, Lezius S, Schmid C, et al. Obstructive sleep apnea and craniofacial appearance in MPS type I-Hurler children after hematopoietic stem cell transplantation. Sleep Breath. 2019;23:1315–21.
248. Dudoignon B, Amaddeo A, Frapin A, Thierry B, de Sanctis L, Arroyo JO, et al. Obstructive sleep apnea in Down syndrome: Benefits of surgery and noninvasive respiratory support. Am J Med Genet A. 2017;173:2074–80.
249. Ghergan A, Coupaye M, Leu-Semenescu S, Attali V, Oppert J-M, Arnulf I, et al. Prevalence and Phenotype of Sleep Disorders in 60 Adults With Prader-Willi Syndrome. Sleep. 2017;40.
250. Giménez S, Videla L, Romero S, Benejam B, Clos S, Fernández S, et al. Prevalence of Sleep Disorders in Adults With Down Syndrome: A Comparative Study of Self-Reported, Actigraphic, and Polysomnographic Findings. J Clin Sleep Med. 2018;14:1725–33.
251. Nao T, Rahmawati A, Nishizaka M, Sawatari H, Moriyama N, Chishaki A, et al. Prevalence of sleep-disordered breathing in Japanese children: efficiency of screening with nocturnal pulse oximetry. Sleep Biol Rhythms. 2020;18:37–47.
252. Killian JT, Lane JB, Lee H-S, Skinner SA, Kaufmann WE, Glaze DG, et al. Scoliosis in Rett Syndrome: Progression, Comorbidities, and Predictors. Pediatr Neurol. 2017;70:20–5.
253. Canora A, Franzese A, Mozzillo E, Fattorusso V, Bocchino M, Sanduzzi A. Severe obstructive sleep disorders in Prader-Willi syndrome patients in southern Italy. Eur J Pediatr. 2018;177:1367–70.
254. Salehi P, Stafford HJ, Glass RP, Leavitt A, Beck AE, McAfee A, et al. Silent aspiration in infants with Prader-Willi syndrome identified by videofluoroscopic swallow study. Medicine (Baltimore). 2017;96:e9256.
255. Fan Z, Ahn M, Roth HL, Li L, Vaughn BV. Sleep Apnea and Hypoventilation in Patients with Down Syndrome: Analysis of 144 Polysomnogram Studies. Children (Basel). 2017;4.
256. Boban S, Leonard H, Wong K, Wilson A, Downs J. Sleep disturbances in Rett syndrome: Impact and management including use of sleep hygiene practices. Am J Med Genet A. 2018;176:1569–77.
257. Trickett J, Heald M, Oliver C. Sleep in children with Angelman syndrome: Parental concerns and priorities. Res Dev Disabil. 2017;69:105–15.
258. Yau S, Pickering RM, Gringras P, Elphick H, Evans HJ, Farquhar M, et al. Sleep in infants and toddlers with Down syndrome compared to typically developing peers: looking beyond snoring. Sleep Med. 2019;63:88–97.
259. Abel EA, Tonnsen BL. Sleep phenotypes in infants and toddlers with neurogenetic syndromes. Sleep Med. 2017;38:130–4.
260. Stores RJ. Sleep problems in adults with Down syndrome and their family carers. J Appl Res Intellect Disabil. 2019;32:831–40.
261. Choi EK, Jung E, Riper MV, Lee YJ. Sleep problems in Korean children with Down syndrome and parental quality of life. Journal of Intellectual Disability Research. 2019;63:1346–58.
262. Lecka-Ambroziak A, Jędrzejczak M, Wysocka-Mincewicz M, Szalecki M. Sleep-related breathing disorders in patients with Prader-Willi syndrome depending on the period of growth hormone treatment. Endokrynol Pol. 2017;68:676–81.
263. Donze SH, de Weerd AW, van den Bossche RAS, Joosten KFM, Hokken-Koelega ACS. Sleep-related breathing disorders in young adults with Prader-Willi syndrome: a placebo-controlled, cross-over GH trial. J Clin Endocrinol Metab. 2019;
264. Lehtonen A, Rust S, Jones S, Brown R, Hare D. Social Functioning and Behaviour in Mucopolysaccharidosis IH [Hurlers Syndrome]. JIMD Rep. 2017;39:75–81.
265. Dekker AD, Sacco S, Carfi A, Benejam B, Vermeiren Y, Beugelsdijk G, et al. The Behavioral and Psychological Symptoms of Dementia in Down Syndrome (BPSD-DS) Scale: Comprehensive Assessment of Psychopathology in Down Syndrome. J Alzheimers Dis. 2018;63:797–819.
266. Kenth JJ, Thompson G, Fullwood C, Wilkinson S, Jones S, Bruce IA. The characterisation of pulmonary function in patients with mucopolysaccharidoses IVA: A longitudinal analysis. Mol Genet Metab Rep. 2019;20:100487.
267. Jayaratne YSN, Elsharkawi I, Macklin EA, Voelz L, Weintraub G, Rosen D, et al. The facial morphology in Down syndrome: A 3D comparison of patients with and without obstructive sleep apnea. Am J Med Genet A. 2017;173:3013–21.
268. Merbler AM, Byiers BJ, Garcia JJ, Feyma TJ, Symons FJ. The feasibility of using actigraphy to characterize sleep in Rett syndrome. Journal of Neurodevelopmental Disorders. 2018;10:8.
269. Eisengart JB, King KE, Shapiro EG, Whitley CB, Muenzer J. The nature and impact of neurobehavioral symptoms in neuronopathic Hunter syndrome. Mol Genet Metab Rep [Internet]. 2019 [cited 2020 Dec 8];22. Available from: <https://www.ncbi.nlm.nih.gov/pmc/articles/PMC6931227/>
270. Cornacchia M, Sethness J, Alapat P, Lin Y-H, Peacock C. The Prevalence of OSA Among an Adult Population With Down Syndrome Referred to a Medical Clinic. Am J Intellect Dev Disabil. 2019;124:4–10.
271. Waters KA, Castro C, Chawla J. The spectrum of obstructive sleep apnea in infants and children with Down Syndrome. Int J Pediatr Otorhinolaryngol. 2020;129:109763.
272. de Vries PJ, Belousova E, Benedik MP, Carter T, Cottin V, Curatolo P, et al. TSC-associated neuropsychiatric disorders (TAND): findings from the TOSCA natural history study. Orphanet J Rare Dis. 2018;13:157.
273. Elsharkawi I, Gozal D, Macklin EA, Voelz L, Weintraub G, Skotko BG. Urinary biomarkers and obstructive sleep apnea in patients with Down syndrome. Sleep Med. 2017;34:84–9.
