# Supplementary material for: Sleep disorders in rare genetic syndromes: a meta-analysis of prevalence and profile
Source: Mol Autism. 2021 Feb 25;12:18. doi: 10.1186/s13229-021-00426-w (PMC7908701; doi:10.1186/s13229-021-00426-w)
Supplement: Supplementary file 7 — Additional file 7. Summary of prevalence rates for each sleep disorder and ‘general’ sleep difficulties across nineteen genetic syndromes. [file 13229_2021_426_MOESM7_ESM.docx]

Additional File 7

| *Summary of prevalence rates for each sleep disorder and ‘general’ sleep difficulties across nineteen genetic syndromes.* *Confidence intervals are presented in parentheses.* | | | | | | | | | | | | | | |
| --- | --- | --- | --- | --- | --- | --- | --- | --- | --- | --- | --- | --- | --- | --- |
| Syndrome | Sleep-related Breathing Difficulties | | Insomnia | | Excessive Daytime Sleepiness | | Sleep Enuresis | | Sleep Bruxism | | ‘General’ | | OVERALL | |
|  | Random | Quality | Random | Quality | Random | Quality | Random | Quality | Random | Quality | Random | Quality | Random | Quality |
| DS | **42%**  **(35-49)** | **32%**  **(21-43)** | 36%  (27-45) | **42%**  **(29-56)** | 29%  (19-39) | 25%  (9-43) | 18%  (10-27) | 18%  (9-29) | 31%  (26-38) | **33%**  **(26-39)** | 36%  (23-50) | 19%  (0-47) | 36%  (32-40) | 28%  (20-36) |
| PWS | **46%**  **(36-55)** | **43%**  **(25-61)** | 29%  (13-47) | 29%  (11-50) | **50%**  **(31-69)** | 38%  (17-61) | 7%  (3-13) | 9%  (3-15) | - | - | 35%  (27-43) | 34%  (26-43) | 42%  (35-49) | 39%  (27-52) |
| AS | 4%  (0-13) | 2%  (0-9) | **54%**  **(43-64)** | **56%**  **(42-69)** | 22%  (9-36) | 19%  (6-34) | **71%**  **(35-99)** | **65%**  **(27-97)** | 16%  (6-28) | 17%  (6-29) | **68%**  **(54-80)** | **70%**  **(53-86)** | 46%  (35-57) | 42%  (30-55) |
| WS | **46%**  **(22-71)** | **47%**  **(21-73)** | **57%**  **(12-97)** | **69%**  **(20-100)** | 30%  (5-60) | 36%  (6-70) | **42%**  **(18-67)** | 36%  (11-63) | 11%  (5-18) | 11%  (5-18) | 33%  (24-43) | 33%  (23-43) | 38%  (28-49) | 40%  (28-53) |
| Rett | 15%  (1-34) | 18%  (1-40) | **59%**  **(44-73)** | **67%**  **(49-85)** | **58%**  **(0-100)** | 37%  (0-100) | - | - | 42%  (19-65) | 38%  (13-63) | **77%**  **(68-85)** | **73%**  **(62-83)** | 55%  (45-66) | 61%  (48-73) |
| FXS | 19%  (0-48) | **25%**  **(0-59)** | 28%  (5-56) | 11%  (0-47) | **40%**  **(7-76)** | 23%  (0-74) | **38%**  **(29-48)** | **38%**  **(29-48)** | 14%  (0-59) | **28%**  **(0-91)** | **37%**  **(32-42)** | **35%**  **(30-41)** | 31%  (23-40) | 24%  (12-38) |
| NF | 5%  (2-9) | 6%  (2-10) | **46%**  **(0-95)** | **49%**  **(2-98)** | **23%**  **(4-48)** | **27%**  **(5-52)** | 2%  (0-7) | 2%  (0-7) | 3%  (0-9) | 3%  (0-9) | **26%**  **(0-65)** | 10%  (0-61) | 21%  (8-37) | 11%  (0-44) |
| CdLs | 23%  (10-38) | **23%**  **(10-38)** | **31%**  **(4-65)** | **28%**  **(1-62)** | 22%  (5-43) | 20%  (3-40) | - | - | 2%  (0-9) | 2%  (0-9) | **32%**  **(0-73)** | **29%**  **(0-70)** | 23%  (13-36) | 21%  (11-34) |
| SMS | 23%  (11-38) | 23%  (11-38) | 59%  (26-89) | 56%  (23-88) | 62%  (48-75) | 60%  (46-74) | **79%**  **(65-91)** | **79%**  **(65-91)** | 44%  (28-60) | 44%  (28-60) | **92%**  **(64-100)** | **95%**  **(68-100)** | 65%  (49-80) | 61%  (44-77) |
| Hurler | 52%  (39-65) | 53%  (39-68) | - | - | - | - | - | - | - | - | **64%**  **(58-70)** | **65%**  **(59-72)** | 55%  (46-64) | 61%  (48-73) |
| CHARGE | **38%**  **(21-56)** | **40%**  **(23-58)** | **57%**  **(47-68)** | **57%**  **(47-68)** | 17%  (10-25) | 17%  (10-25) | - | - | - | - | 34%  (0-85) | 32%  (0-83) | 36%  (23-51) | 38%  (24-53) |
| MPS IIIB | 46%  (0-100) | 21%  (0-100) | 68%  (36-95) | 58%  (21-93) | - | - | - | - | - | - | **82%**  **(70-91)** | **83%**  **(72-93)** | 70%  (51-85) | 68%  (48-85) |
| TSC | 6%  (0-16) | 6%  (0-16) | **51%**  **(34-68)** | **52%**  **(33-70)** | **46%**  **(31-60)** | **46%**  **(31-60)** | - | - | - | - | 42%  (28-58) | 45%  (24-66) | 43%  (33-53) | 45%  (27-63) |
| CdC | 3%  (0-14) | 3%  (0-14) | **30%**  **(15-48)** | **30%**  **(15-48)** | 13%  (3-28) | 13%  (3-28) | **37%**  **(20-55)** | **37%**  **(20-55)** | 13%  (3-28) | 13%  (3-28) | **36%**  **(0-93)** | **35%**  **(0-92)** | 23%  (9-39) | 23%  (9-40) |
| MPS II | **77%**  **(53-95)** | **77%**  **(53-97)** | - | - | - | - | - | - | - | - | 84%  (34-100) | 74%  (17-100) | 78%  (60-92) | 79%  (60-93) |
| JS | 2%  (0-10) | 2%  (0-10) | - | - | 9%  (2-20) | 9%  (2-20) | **44%**  **(30-59)** | **44%**  **(30-59)** | **28%**  **(15-42)** | **28%**  **(15-42)** | **25%**  **(16-36)** | **22%**  **(9-37)** | 20%  (8-35) | 21%  (7-40) |
| SLOS | 50%  (27-73) | 50%  (27-73) | 56%  (32-78) | 56%  (32-78) | 44%  (22-68) | 44%  (22-68) | **78%**  **(55-94)** | **78%**  **(55-94)** | 39%  (17-63) | 39%  (17-63) | **70%**  **(48-88)** | **70%**  **(48-88)** | 56%  (44-68) | 58%  (46-70) |
| JNCL | - | - | 46%  (28-65) | 46%  (28-65) | 36%  (19-55) | 36%  (19-55) | - | - | - | - | **100%**  **(79-100)** | **100%**  **(79-100)** | 63%  (24-95) | 60%  (19-95) |
| MPS IV | **73%**  **(58-86)** | **72%**  **(57-86)** | - | - | - | - | - | - | - | - | 44%  (25-64) | 44%  (25-64) | 64%  (47-80) | 67%  (49-83) |
| OVERALL | 41%  (36-46) | 32%  (23-41) | 46%  (39-52) | 45%  (34-56) | 34%  (28-40) | 30%  (22-38) | 29%  (20-39) | 32%  (22-44) | 26%  (20-31) | 26%  (20-33) | 50%  (43-58) | 32%  (16-50) | - | - |

Notes: Syndromes are presented in order of the size of the evidence base. Syndrome-related prevalence rates above the overall pooled prevalence in each syndrome are in bold.
