# Supplementary material for: Sleep disorders in rare genetic syndromes: a meta-analysis of prevalence and profile
Source: Mol Autism. 2021 Feb 25;12:18. doi: 10.1186/s13229-021-00426-w (PMC7908701; doi:10.1186/s13229-021-00426-w)
Supplement: Supplementary file 8 — Additional file 8. Detailed sleep disorder forest plots. [file 13229_2021_426_MOESM8_ESM.docx]

Additional File 8

Sleep-related breathing difficulties

In total, 165 papers reported on sleep-related breathing difficulties (SRBD) in 18 genetic syndromes, though the majority of these focused on Down syndrome (n=69, 41.8%) and Prader-Willi syndrome (n=36, 28.8%). These papers gave varying definitions for SRBD, including ‘breath holding during sleep’, ‘central sleep apnoea’ and ‘obstructive sleep apnoea’. The majority of studies used polysomnography or oximetry (n=94, 57%) to diagnose SRBD, though many also relied on questionnaire measures or parent-report. Overall, the quality of these papers ranged from 0.17 (poor) to 0.92 (excellent), but only 20 (12.1%) papers received a score of 0.76 or above indicating that their quality was ‘excellent’.

The random effects model reveals that the overall prevalence of SRBD across genetic syndromes is reasonably high (41%, with confidence intervals of 36-46%), though the quality-effects model produced a more conservative estimate of 32% (CI 23-41%). The pooled prevalence of SRBD was highest in MPS II (77%, CI 53-97%) and lowest in Angelman syndrome (2%, CI 0-9%). The forest plot, based on the quality-effects model of these 165 papers, is presented in Figure 1 which is laid out over five pages to aid the reader. Importantly, the prevalence of SRBD was higher in eight genetic syndromes than estimates for typically developing (TD) adults diagnosed by Apnoea-Hypopnea Index alone (27%), and in fifteen syndromes if TD adults also had to demonstrate daytime impairment for diagnosis (4%; Sanders & Givelber, 2005). Estimates were also higher in these syndromes than in TD children (7.45%; Lumeng & Chervin, 2008).

**Additional Figure 1a** Quality-effects model of sleep related breathing difficulties across genetic syndromes

**Additional Figure 1b** Quality-effects model of sleep related breathing difficulties across genetic syndromes

**Additional Figure 1c** Quality-effects model of sleep related breathing difficulties across genetic syndromes

**Additional Figure 1d** Quality-effects model of sleep related breathing difficulties across genetic syndromes

**Additional Figure 1e** Quality-effects model of sleep related breathing difficulties across genetic syndromes

Insomnia

In total, 74 papers reported on insomnia in 15 genetic syndromes, with 23 of these on participants with Down syndrome (31.1%). A range of questionnaire measures were used, including the Children’s Sleep Habits Questionnaire (n=13, 17.6%), Sleep Disturbance Scale for Children (n=6, 8.1%), Behavioural Evaluation of Disorders of Sleep (n=3, 4.1%), and Paediatric Sleep Questionnaire (n=3, 4.1%). Ten papers (13.5%) used modified versions of the Simonds & Parraga Sleep Questionnaire and one utilised the Pittsburgh Sleep Quality Index. Overall, the quality of these papers ranged from 0.17 (poor) to 0.92 (excellent), but only seven (9.5%) received an ‘excellent’ quality rating.

The random-effects model produced an overall prevalence estimate of insomnia within genetic syndromes of 46% (CI 39-52%), marginally higher than the quality-effects estimate of 45% (CI 34-56%). The pooled prevalence was highest in Williams syndrome (69%, CI 20-100%), and lowest in fragile X syndrome (11%, CI 0-47%) but these estimates should be interpreted cautiously, given the wide range of confidence intervals. The forest plot, which displays pooled prevalence estimates for a quality-effects model of all 74 studies, is presented over three pages in Figure 2 to aid the reader. Crucially, the prevalence of insomnia in all 14 syndromes (except fragile X) was elevated compared to TD estimates in adults (9-10%; Brown, 2005) and children (25%; Owens, 2008).

**Additional Figure 2a** Quality-effects model of insomnia across genetic syndromes

**Additional Figure 2b** Quality-effects model of insomnia across genetic syndromes

**Additional Figure2c** Quality-effects model of insomnia across genetic syndromes

Excessive daytime sleepiness

In total, 68 papers reported on excessive daytime sleepiness (including napping, feeling drowsy and falling asleep in various daytime situations) in 15 genetic syndromes. Nineteen (27.9%) of these papers reported on Down syndrome and another 16 (23.5%) on Prader-Willi syndrome. A substantial proportion of studies used the standard or paediatric version of the Epworth Sleepiness Scale (n=19, 27.9%), though some utilised general sleep questionnaires or parent-report. Only six (8.8%) used the multiple sleep latency test to measure excessive daytime sleepiness. Overall, the quality of these papers ranged from 0.17 (poor) to 0.75 (good), though only 14 (20.6%) were rated as ‘good’. None of the papers received an ‘excellent’ quality rating.

The random-effects model produced an overall prevalence estimate of excessive daytime sleepiness within genetic syndromes of 34% (CI 28-40%), similar to the quality-effects estimate of 30% (CI 22-38%). The pooled prevalence of excessive daytime sleepiness was highest in Smith-Magenis syndrome (60%, CI 46-74%), and lowest in Angelman syndrome (19%, CI 6-34%) but elevated compared to TD estimates in 13 syndromes (13% Hayley et al., 2014). The quality-effects model of all 68 studies is presented in Figure 3, laid out over three pages to aid the reader.

**Additional Figure 3a** Quality-effects model of excessive daytime sleepiness across genetic syndromes

**Additional Figure 3b** Quality-effects model of excessive daytime sleepiness across genetic syndromes

**Additional Figure 3c** Quality-effects model of excessive daytime sleepiness across genetic syndromes

Sleep enuresis

Thirty papers reported on sleep enuresis (bedwetting) in 10 genetic syndromes. Once again, individuals with Down syndrome were the most common participant group (n=12, 40%). Only four studies (13.3%) utilised the Parental questionnaire: Enuresis/Urinary Incontinence, which resulted in the maximum quality score for sleep assessment. The remaining studies used general sleep questionnaires or asked parents about their child’s bedwetting. The total quality rating of these studies ranged from 0.25 (adequate) to 0.75 (good), with nine papers (30%) assigned a ‘good’ quality rating, but no papers reached criteria for an ‘excellent’ rating.

The random effects model produced an overall prevalence of sleep enuresis within genetic syndromes of 29% (CI 20-39%). The quality-effects model produced a slightly higher pooled prevalence estimate of 32% (CI 22-44%). The pooled prevalence of sleep enuresis was highest in Angelman syndrome (65%, CI 27-97%), and lowest in neurofibromatosis (2%, CI 0-7%) but the latter estimate should be interpreted cautiously, given the low confidence interval. In the remaining 9 syndromes, the overall prevalence of sleep enuresis was elevated above the prevalence for TD children aged 10 years or older (3-4%, Challamel & Cochat, 2005). The quality-effects forest plot, based on all 27 studies, is presented in Figure 4.

**Additional Figure 4** Quality-effects model of sleep enuresis across genetic syndromes

Sleep bruxism

Only 28 papers reported on sleep bruxism (teeth grinding) in 11 genetic syndromes. Thirteen papers (46.4%) reported on sleep bruxism in Down syndrome. Most studies received a ‘good’ quality rating of 0.5 or above, having used a general sleep questionnaire to consider sleep bruxism within a range of other sleep-related behaviours. Only two (7.1%) studies used a clinical or dental examination to assess for sleep bruxism. The total quality rating of these studies ranged from 0.16 (poor) to 0.75 (good), with eight papers (28.6%) assigned a ‘good’ rating.

The random-effects model produced an overall prevalence of sleep bruxism within genetic syndromes of 26% (CI 20-31%), as did the quality-effects model with slightly wider confidence intervals (20-33%). The forest plot incorporating all 28 studies in a quality-effects model is presented in Figure 5. The pooled prevalence of sleep bruxism was highest in Rett syndrome (38%, CI 13-63%), and lowest in Williams syndrome (11%, CI 5-18%) but the relative risk did not differ between these syndromes due to wide confidence intervals. With the exception of Cornelia de Lange syndrome and neurofibromatosis, both of which had a lower confidence interval of 0, all eight syndromes showed an elevated prevalence of sleep bruxism compared to TD estimates (5-8%; Bader, 2005).

**Additional Figure 5** Quality-effects model of sleep bruxism across genetic syndromes

‘General’ sleep difficulties

In addition to the papers outlined above considering specific sleep disorders, 98 studies reported the prevalence of ‘general’ sleep difficulties in 19 genetic syndromes. In many cases, little definition was given for these ‘general’ difficulties but in some the prevalence rate represented the number of participants who had a total score above a clinical cut-off, encompassing multiple aspects of sleep difficulty. These papers received the highest quality rating for their definition of ‘general’ sleep difficulty. The total quality rating of these studies ranged from 0.08 (poor) to 0.92 (excellent), though only two papers received an ‘excellent’ rating. Thirty-four papers were rated as ‘good’ (34.7%).

The random effects model produced an overall prevalence of ‘general’ sleep difficulties within genetic syndromes of 50% (CI 43-58%), but the quality-effects model produced a much more conservative estimate of 32% (16-50%), similar to the estimate for insomnia in TD children (25%, Owens, 2008). When considering each syndrome separately, prevalence estimates varied widely (e.g. from 10% in Neurofibromatosis to 95% in Smith-Magenis syndrome). The prevalence rates in Angelman and Rett syndromes and MPS IIIB were each higher than in four-five other syndromes. The quality effects model is presented in Figure 6, laid out over four pages to aid the reader.

**Additional Figure 6a** Quality-effects model of ‘general ‘sleep difficulties across genetic syndromes

**Additional Figure 6b** Quality-effects model of ‘general ‘sleep difficulties across genetic syndromes

**Additional Figure 6c** Quality-effects model of ‘general ‘sleep difficulties across genetic syndromes

**Additional Figure 6d** Quality-effects model of ‘general ‘sleep difficulties across genetic syndromes
