# Supplementary material for: Sleep disorders in rare genetic syndromes: a meta-analysis of prevalence and profile
Source: Mol Autism. 2021 Feb 25;12:18. doi: 10.1186/s13229-021-00426-w (PMC7908701; doi:10.1186/s13229-021-00426-w)
Supplement: Supplementary file 9 — Additional file 9. Detailed syndrome forest plots. [file 13229_2021_426_MOESM9_ESM.docx]

Additional File 9

Syndromes are presented in order of the size of the evidence base, from syndromes reported in the most papers to syndromes reported in the least.

*Down syndrome*

By far the most studied of the syndromes included in this meta-analysis, 164 separate prevalence estimates were reported for various types of sleep disorder/difficulty in Down syndrome, in 89 papers (see Figure 1). Sixty-nine prevalence estimates were reported for SRBD, 23 for insomnia, 19 for excessive daytime sleepiness, 13 for sleep bruxism and 12 for sleep enuresis. Twenty-eight papers reported on ‘general’ sleep difficulties. Twenty-four studies reported on multiple sleep disorders in Down syndrome.

The overall prevalence of any sleep disorder/difficulty in Down syndrome was 36% (CI 32-40%) according to the random effects model, but 28% (CI 20-36%) according to the quality-effects model. Insomnia was the most prevalent of all sleep disorders (42%), closely followed by sleep-related breathing difficulties (32%). Sleep enuresis was the least common according to the quality-effects model.

**Additional Figure 1a** Quality-effects model of sleep disorders and difficulties in Down syndrome

**Additional Figure 1b** Quality-effects model of sleep disorders and difficulties in Down syndrome

**Additional Figure 1c** Quality-effects model of sleep disorders and difficulties in Down syndrome

*Prader-Willi syndrome*

Participants with Prader-Willi syndrome were included in 54 separate studies of sleep disorders. These studies reported 76 prevalence estimates across the different categories of sleep disorder. As in Down syndrome, SRBD were considered in the majority of papers, producing 47 prevalence estimates. Sixteen papers reported on excessive daytime sleepiness, five on insomnia and four on sleep enuresis. None of the studies reported the prevalence of sleep bruxism in Prader-Willi syndrome. Only four studies descried ‘general’ sleep difficulties. Fourteen studies reported on multiple sleep disorders in Prader-Willi syndrome.

The overall prevalence of any sleep disorder/difficulty in Prader-Willi syndrome was 42% (CI 35-49%) according to the random effects model, and 39% (CI 27-52%) according to the quality-effects model. SRBD were the most prevalent (43%), followed by excessive daytime sleepiness (38%). Sleep enuresis was the least prevalent sleep disorder studied (9%).

**Additional Figure 2a** Quality-effects model of sleep disorders and difficulties in Prader-Willi syndrome

**Additional Figure 2b** Quality-effects model of sleep disorders and difficulties in Prader-Willi syndrome

*Angelman syndrome*

Thirty-seven prevalence estimates for each of the sleep disorders in Angelman syndrome were reported in 20 separate papers. Insomnia (10 studies) and ‘general’ sleep difficulties (12 studies) were the most often studied. Excessive daytime sleepiness was reported in six studies, SRBD in four studies and sleep bruxism in two. Three papers reported the prevalence of sleep enuresis in Angelman syndrome. Seven studies reported on more than one type of sleep disorder.

The overall prevalence of any sleep disorder/difficulty in Angelman syndrome was 46% (CI 35-57%) according to the random effects model, and 42% (CI 30-55%) according to the quality-effects model. SRBD were the least prevalent (2%), while ‘general’ difficulties were the most prevalent (70%).

**Additional Figure 3a** Quality-effects model of sleep disorders and difficulties in Angelman syndrome

**Additional Figure 3b** Quality-effects model of sleep disorders and difficulties in Angelman syndrome

*Rett syndrome*

Rett syndrome was the focus of 19 separate studies, producing a total of 28 prevalence estimates. Insomnia and ‘general’ sleep difficulties were the most commonly described, each reported in nine studies. Four papers reported on SRBD, three described excessive daytime sleepiness and three reported on sleep bruxism. Six studies reported the prevalence of more than one type of sleep disorder in individuals with Rett syndrome.

The overall prevalence of any sleep disorder/difficulty in Rett syndrome was 55% (CI 45-66%) according to the random effects model, and 61% (CI 48-73%) according to the quality-effects model. ‘General’ sleep difficulties were the most prevalent (73%), while SRBD were the least prevalent (18%).

**Additional Figure 4** Quality-effects model of sleep disorders and difficulties in Rett syndrome

*Williams syndrome*

Thirteen separate studies reported a total of 27 prevalence estimates for various types of sleep disorder in Williams syndrome. After ‘general’ sleep difficulties (reported on in seven studies), SRBD and excessive daytime sleepiness were the most common focus of these studies, each reported on in five papers. Four papers reported on insomnia, four on sleep enuresis and two on sleep bruxism. Five studies reported the prevalence of more than one type of sleep disorder in individuals with Williams syndrome.

The overall prevalence of any sleep disorder in Williams syndrome was 38% (CI 28-49%) according to the random effects model, and 40% (CI 28-53%) according to the quality-effects model. Insomnia was the most prevalent (69%), while bruxism was the least prevalent (11%). Excessive daytime sleepiness and sleep enuresis were reported in 36% participants.

**Additional Figure 5** Quality-effects model of sleep disorders and difficulties in Williams syndrome

*Fragile X syndrome*

Nineteen prevalence estimates, derived from seven individual studies, were available for poor sleep in fragile X syndrome. ‘General’ sleep difficulties were the most commonly studied, reported in five papers. SRBD and insomnia were each considered in three studies, and sleep enuresis and sleep bruxism were considered in two studies each. Three studies reported the prevalence of multiple sleep disorders in fragile X syndrome.

The overall prevalence of any sleep disorder/difficulty in fragile X syndrome was 31% (CI 23-40%) according to the random effects model, and 24% (CI 12-38%) according to the quality-effects model. Sleep enuresis was the most common problem reported (38% according to both models). Insomnia was the least prevalent (11%).

**Additional Figure 6** Quality-effects model of sleep disorders and difficulties in Fragile X syndrome

*Neurofibromatosis*

Six separate studies reported on various sleep disorders/difficulties in Neurofibromatosis. Four studies reported the prevalence of multiple sleep disorders. Excessive daytime sleepiness and insomnia were each reported on by three studies. One paper (Johnson et al., 2005) considered both sleep enuresis and sleep bruxism. ‘General’ sleep difficulties were the most commonly studied, reported by four papers.

The overall prevalence of any sleep disorder/difficulty in neurofibromatosis was 21% (CI 8-37%) according to the random effects model, but just 11% (CI 0-44%) according to the quality-effects model. Given the low confidence intervals, this model should be interpreted with caution. Insomnia was by far the most prevalent sleep disorder, experienced by 49% of participants. SRBD, sleep enuresis and sleep bruxism were reported at very low rates.

**Additional Figure 7** Quality-effects model of sleep disorders and difficulties in neurofibromatosis

*Smith-Magenis syndrome*

Twelve prevalence estimates for sleep disorders in Smith-Magenis syndrome were available from eight separate studies. One paper (Smith et al., 1998b) reported on all five specific sleep disorders. Three papers reported on insomnia, and two papers reported on excessive daytime sleepiness. Four studies reported the prevalence of ‘general’ sleep difficulties.

The overall prevalence of any sleep disorder/difficulty in Smith-Magenis syndrome was 65% (CI 49-80%) according to the random effects model and 61% (CI 44-77%) according to the quality-effects model. ‘General’ sleep difficulties were by far the most prevalent, reported in 95% individuals. Sleep enuresis was also very common (79%). SRBD were the least prevalent (23%).

**Additional Figure 8** Quality-effects model of sleep disorders and difficulties in Smith-Magenis syndrome

*Hurler syndrome*

Eleven studies presented twelve prevalence estimate for poor sleep in Hurler syndrome. Eight of these reported on SRBD and four on ‘general’ sleep difficulties.

The overall prevalence of any sleep disorder/difficulty in Hurler syndrome was 55% (CI 46-64%) according to the random effects model, but 61% (CI 48-73%) according to the quality-effects model. ‘General’ sleep difficulties (65%) were more prevalent than SRBD (53%).

**Additional Figure 9** Quality-effects model of sleep disorders and difficulties in Hurler syndrome

*Tuberous sclerosis complex*

Eight separate studies described sleep in tuberous sclerosis complex, producing eleven prevalence estimates. Five of these were for insomnia, two on excessive daytime sleepiness and one on SRBD. Three reported ‘general’ sleep difficulties, but no papers reported prevalence of sleep bruxism or sleep enuresis.

The overall prevalence of any sleep disorder/difficulty in tuberous sclerosis complex was 43% (CI 33-39%) according to the random effects model, and 45% (CI 27-63%) according to the quality-effects model. Insomnia was the most prevalent disorder (52%) and SRBD were least prevalent (6%), but as only one study reported on this represents one study’s estimate rather than a meta-analysis.

**Additional Figure 10** Quality-effects model of sleep disorders and difficulties in tuberous sclerosis complex

*Cornelia de Lange syndrome*

Ten prevalence estimates for sleep disorders were drawn from four separate studies in Cornelia de Lange syndrome. Three studies reported the prevalence of multiple sleep disorders. Excessive daytime sleepiness was reported in three studies. Insomnia, SRBD and ‘general’ sleep difficulties were each reported on by two studies. One study reported the prevalence of sleep bruxism, but there were no studies looking at the prevalence of sleep enuresis.

The overall prevalence of any sleep disorder/difficulty in Cornelia de Lange syndrome was 23% (CI 13-36%) according to the random effects model, or 21% (CI 11-34%) according to the quality-effects model. ‘General’ difficulties and insomnia were the most common problems (29% and 28% respectively). Sleep bruxism was the least prevalent (2%).

**Additional Figure 11** Quality-effects model of sleep disorders and difficulties in Cornelia de Lange syndrome

*Mucopolysaccharidosis Type IIIB*

Eight studies considered sleep in MPS IIIB, producing ten prevalence estimates. Five of these reported on ‘general’ sleep difficulties, three on insomnia and one on SRBD. Two papers reported on multiple sleep disorders.

The overall prevalence of any sleep disorder/difficulty in MPS IIIB was 70% (CI 51-85%) according to the random effects model, and 68% (CI 48-85%) according to the quality-effects model. In particular, ‘general’ sleep difficulties were highly prevalent (83%).

**Additional Figure 12** Quality-effects model of sleep disorders and difficulties in Mucopolysaccharidosis Type IIIB

*Mucopolysaccharidosis Type II*

Nine individual studies each reported a prevalence of poor sleep in MPS II. Seven of these described the prevalence of SRBD, and one (Bax & Colville, 1995) a ‘general’ difficulty.

The overall prevalence of any sleep disorder/difficulty in MPS II was 78% according to the random effects model (CI 60-92%) and 79% (CI 60-93%) according to the quality-effects model. This was largely driven by the extremely high prevalence of SRBD (77%).

**Additional Figure 13** Quality-effects model of sleep disorders and difficulties in Mucopolysaccharidosis Type II

*CHARGE syndrome*

Five studies presented eight prevalence estimates for a sleep disorder in CHARGE syndrome. Two studies by Hartshorne and colleagues (2009, 2016) reported on multiple sleep disorders. Four studies reported on SRBD, one on insomnia, one on excessive daytime sleepiness and two on ‘general’ sleep difficulties. No studies reported on the prevalence of sleep enuresis or sleep bruxism in CHARGE syndrome. The overall prevalence of any sleep disorder/difficulty in CHARGE syndrome was 36% (CI 23-51%) according to the random effects model, and 38% (CI 24-53%) according to the quality-effects model. Insomnia was the most prevalent sleep disorder (57% according to both models), while excessive daytime sleepiness only occurred in 17% of participants according to both models.

**Additional Figure 14** Quality-effects model of sleep disorders and difficulties in CHARGE syndrome

*Cri du Chat syndrome*

Only two papers reported the prevalence of poor sleep in Cri du Chat syndrome, both by Maas and colleagues (2009, 2012). The first used a modified version of the Simmonds and Parraga (1982) sleep questionnaire to ask about the presence or absence of excessive daytime sleepiness, insomnia, sleep enuresis, SRBD and sleep bruxism as well as ‘general’ sleep difficulties. Maas et al (2012) report the prevalence of a ‘general’ sleep difficulty only, so only this type of problem could be meta-analysed. The other studies are included in the random and quality-effects pooled prevalence models in order to calculate the prevalence of any sleep disorder/difficulty in Cri du Chat syndrome.

Overall, the prevalence of any sleep disorder/difficulty in Cri du Chat syndrome was 23% according to both models, though with slightly differing confidence intervals. ‘General’ difficulties were reported in 36% of the participants in the random-effects model and 35% of the participants in the quality-effects models.

**Additional Figure 15** Quality-effects model of sleep disorders and difficulties in Cri du Chat syndrome

*Jacobsen syndrome*

Only two papers, both by Maas and colleagues (2005, 2012), reported the prevalence of sleep disorders/difficulties in Jacobsen syndrome. Maas et al. (2005) used a modified version of the Simmonds and Parraga (1982) sleep questionnaire, reporting the presence or absence of SRBD, sleep enuresis, excessive daytime sleepiness and sleep bruxism as well as ‘general’ sleep problems. Neither paper reported on the prevalence of insomnia in Jacobsen syndrome. Maas et al. (2012) report the prevalence of a ‘general’ sleep difficulty only, so only this type of problem could be meta-analysed. The other studies are included in the random and quality-effects pooled prevalence) in order to calculate the overall prevalence of any sleep disorder/difficulty in Jacobsen syndrome, which was 20% (CI 8-35%) according to the random effects model or 21% (CI 7-39%) according to the quality effects model.

**Additional Figure 16** Quality-effects model of sleep disorders and difficulties in Jacobsen syndrome

*Smith-Lemli-Opitz syndrome*

Two papers reported the prevalence of poor sleep in Smith-Lemli-Opitz syndrome. Zarowski et al. (2011) describe the prevalence of each of the five ICSD sleep disorders. Freeman et al. (2016) report only the prevalence of ‘general’ sleep difficulties in this syndrome. Thus, the studies could not be meta-analysed to calculate a pooled prevalence estimate for any specific sleep disorder. The overall prevalence of any sleep disorder/difficulty in Smith-Lemli-Opitz syndrome was 56% (CI 44-68%) according to the random effects model and 58% according to the quality effects model (CI 46-70%).

**Additional Figure 17** Quality-effects model of sleep disorders and difficulties in Smith-Lemli-Opitz syndrome

*Mucopolysaccharidosis Type IV*

Four papers reported on poor sleep in MPS IV, three on SRBD and one on ‘general’ sleep difficulties. The random effects model gave an overall prevalence of any sleep disorder/difficulty in MPS IV as 64% (CI 47-80%), while the quality-effects model produced an estimate of 67% (CI 49-83%).

*Juvenile Neuronal Ceroid Lipofuscinosis*

**Additional Figure 18** Quality-effects model of sleep disorders and difficulties in Mucopolysaccharidosis Type IV

Two papers described the prevalence of three types of poor sleep in juvenile neuronal ceroid lipofuscinosis. Kirveskari et al. (2000) reported the prevalence of insomnia and excessive daytime sleepiness, and Malcolm et al. (2012) described ‘general’ sleep difficulties in this syndrome. Thus, the studies could not be meta-analysed to calculate a pooled prevalence estimate for any specific sleep disorder/difficulty in juvenile neuronal ceroid lipofuscinosis, but the overall prevalence of any sleep disorder/difficulty in juvenile neuronal ceroid lipofuscinois was 63% (CI 24-95%) according to the random effects model and 56% according to the quality effects model (CI 19-95%).

**Additional Figure 19** Quality-effects model of sleep disorders and difficulties in Juvenile Neuronal Ceroid Lipofuscinosis
